# MicroRNA from *Moringa oleifera*: Identification by High Throughput Sequencing and Their Potential Contribution to Plant Medicinal Value

**DOI:** 10.1371/journal.pone.0149495

**Published:** 2016-03-01

**Authors:** Stefano Pirrò, Letizia Zanella, Maurice Kenzo, Carla Montesano, Antonella Minutolo, Marina Potestà, Martin Sanou Sobze, Antonella Canini, Marco Cirilli, Rosario Muleo, Vittorio Colizzi, Andrea Galgani

**Affiliations:** 1 Department of Biology, University of Rome “Tor Vergata”, Rome, Italy; 2 SOCOPOMO S/C 46 P.A. de Lingang, Dschang, Cameroon; 3 University of Dschang, Dschang, Cameroon; 4 Centro di Servizi Interdipartimentale, Stazione per la Tecnologia Animale, University of Rome‘‘Tor Vergata”, Rome, Italy; 5 Mir-Nat s.r.l., Rome, Italy; 6 Department of Agricultural and Forest Science, University of Tuscia, Viterbo, Italy; Kunming University of Science and Technology, CHINA

## Abstract

*Moringa oleifera* is a widespread plant with substantial nutritional and medicinal value. We postulated that microRNAs (miRNAs), which are endogenous, noncoding small RNAs regulating gene expression at the post-transcriptional level, might contribute to the medicinal properties of plants of this species after ingestion into human body, regulating human gene expression. However, the knowledge is scarce about miRNA in *Moringa*. Furthermore, in order to test the hypothesis on the pharmacological potential properties of miRNA, we conducted a high-throughput sequencing analysis using the Illumina platform. A total of 31,290,964 raw reads were produced from a library of small RNA isolated from *M*. *oleifera* seeds. We identified 94 conserved and two novel miRNAs that were validated by qRT-PCR assays. Results from qRT-PCR trials conducted on the expression of 20 *Moringa* miRNA showed that are conserved across multiple plant species as determined by their detection in tissue of other common crop plants. *In silico* analyses predicted target genes for the conserved miRNA that in turn allowed to relate the miRNAs to the regulation of physiological processes. Some of the predicted plant miRNAs have functional homology to their mammalian counterparts and regulated human genes when they were transfected into cell lines. To our knowledge, this is the first report of discovering *M*. *oleifera* miRNAs based on high-throughput sequencing and bioinformatics analysis and we provided new insight into a potential cross-species control of human gene expression. The widespread cultivation and consumption of *M*. *oleifera*, for nutritional and medicinal purposes, brings humans into close contact with products and extracts of this plant species. The potential for miRNA transfer should be evaluated as one possible mechanism of action to account for beneficial properties of this valuable species.

## Introduction

*Moringa oleifera* Lam, a naturalized species from the monogenus family *Moringaceae*, is one of the best known, most widely distributed and most useful nutritional and medicinal plants [1–3]. Several organs of the *Moringa* tree are edible (e.g., pods, seeds, flowers and leaves) and are used in many countries (including many regions of Africa) for their high nutritional value [1–2]. Almost all tissues of this plant can be used in the treatment of inflammation or infectious diseases along with cardiovascular, gastrointestinal, haematological and neoplastic diseases [2–4]. The leaves are a source of natural antioxidants [5], vitamins A, B and C, minerals, proteins and essential amino acids [1–2]. The Italian Ministry of Health in compliance with the European Pharmaceutical Plant legislation has included *Moringa* seeds in the “List of Plant and Vegetal Integrators”.

In 2014, Jung IL reported a tumor suppressor activity in in mammalian cells treated with cold water-soluble extract of *M*. *oleifera* leaves [6]. The author found an abnormal ribosomal RNA (rRNA) pattern and down-regulation of many genes and proteins involved in cell transformation and proliferation in mammalian cells treated with this extract. He concluded that the cold water-soluble extract of *M*. *oleifera* induced rRNA degradation. In 2015, Tian and coworkers reported a high-quality draft genome sequence of *M*. *oleifera* and compared the genome to related woody plant genomes in order to clarify the derivation of this species [7].

Plant miRNAs are a class of 18–24 nucleotide (nt) small, non-coding RNA that negatively regulate specific messenger RNA (mRNA). MiRNAs operate in a sequence-specific manner and silence specific protein-coding genes at the post-transcriptional level by targeting the 3’ untranslated region (3’UTR) of mRNA [8]. This process causes mRNA cleavage and decreases protein translation [9–11]. In general, miRNAs are key regulators of development, stress response, growth and other important physiological processes [12–13].

In 2012, Zhang and collaborators demonstrated that *osa*-*miR-168a* and other exogenous miRNAs that are abundant in rice plants can be acquired by mice through food intake, as evidenced by their presence in sera or tissues of the mammalian. *In vitro* and *in vivo* functional studies showed that these exogenous miRNAs are able to inhibit mammalian gene expression in the liver, demonstrating the first case of cross-kingdom regulation [14]. More recently, oral administered cocktails of endogenous tumor suppressor miRNAs, exhibiting characteristics of plant miRNAs, reduced tumor burden in a mouse model of colon cancer [15]. These observations show that plant miRNAs are absorbed in the mammalian digestive tract and can target mammalian genes. Moreover, they suggest the hypothesis that engineered edible plants producing mammalian tumor suppressor miRNAs might be a new treatment modality for cancer. Such treatment might be an effective, nontoxic, and inexpensive chemo-preventive strategy for human.

Recently, Shu and collaborators presented an integrative study where comparative analysis and computational prediction have been applied to assess the cross-species transportation of miRNAs, particularly focusing on inferring the likelihood of exogenous miRNA in human circulation [16].

This work demonstrated the data-driven computational analysis is highly promising approach to study novel molecular characteristics of deliverable miRNAs allowing to bypass the complex mechanistic details.

Several miRNAs discovery methods including computational prediction, cloning strategies and others have been used [17–19], even though these methods demand an increased rigor in miRNA annotations [20]. High-throughput sequencing technologies have contributed markedly to the expansion of knowledge about the miRNA universe in eukaryotic cells. These technologies have revealed a number of newly evolved and species-specific miRNAs that were previously unknown [21–22].

With the effort to define medicinal factors found in this interesting plant species, we searched for understanding better genome expression and gene regulation by micro RNA (miRNA) in *M*. *oleifera* Lam. We recognize that seeds contain all information about the tissues that will develop in the adult plant; therefore, we have programmed to analyze the miRNA populations obtained from seeds. In this work, we have identified novel miRNAs and their potential target genes from *M*. *oleifera* seeds using Illumina platform technologies. We found 94 conserved miRNAs and two novel ones, and some of them were validated by qRT-PCR. Target genes were predicted by *in silico* and results indicate that *mol*-miRNAs are putatively involved in many physiological process. A selected number of miRNAs were compared to other crop species plants, resulting to be conserved across multiple species. By taking advantage of a recently developed web-application based on an algorithm that compares plant and mammalian miRNAs (http://160.80.35.140/MirCompare), we have identified a few *M*. *oleifera miRNAs* with functional homologies to mammalian ones. We conducted a preliminary analysis to investigate potential human gene regulation by the plant miRNA mimics. The reported analysis increases the information on plant miRNAs currently available and improves the knowledge on to the molecular mechanisms associated to nutritional and medicinal activities of this plant species.

## Materials and Methods

### Plant materials, sample collection and RNA extraction

Seeds collected from mature pods, before they split open and fall to the ground, harvested from *M*. *oleifera* trees grown in the District of Dschang in West Cameroon, without use of chemical fertilizer, by the Cooperative of Medical Plant Producers SOCOPOMO. Seeds were stored at -80°C until used. Seeds were germinated in a greenhouse at the Department of Biology, University of “Tor Vergata”, Rome, by placing them on paper soaked in sterile water. Leaves, stems and roots were collected at one month from the beginning of germination, immediately frozen in liquid nitrogen and subsequently stored at -80°C until used. Tissues from other species (*Solanum tuberosa*, *Olea europaea* and *Medicago sativa*) grown under natural field conditions, were also stored at -80°C until used. Total RNA was extracted from plant tissues using the mirVana kit (Ambion, USA) according to the manufacturer’s protocol. The assessment of RNA quality and quantity were evaluated by Agilent 2100 Bioanalyzer (Agilent Technologies, Waldbroon, Germany) and by spectrophotometry (SmartSpec Plus, Bio-Rad, USA), respectively.

### Constructing and sequencing small RNA libraries

Quality control for next generation sequencing experiments was performed by Genomix4life S.r.l. (Baronissi, Salerno, Italy). Individual indexed libraries were prepared from 1 μg of purified RNA using the TruSeq SmallRNA Sample Prep Kit (Illumina, USA) according to the manufacturer’s instructions. Libraries were quantified using the Agilent 2100 Bioanalyzer (Agilent Technologies, Waldbroon, Germany) and pooled so that each index-tagged sample was present in equimolar amounts with final concentrations of the pooled samples adjusted to 2 nM. The pooled samples were subject to cluster generation and sequencing using an Illumina HiSeq 2500 System (Illumina, USA) in a 1x50 single read format at a final concentration of 10 pmol. The raw sequence files generated underwent quality control analyses using FastQC [23]. This method offers a powerful means for quantitative and qualitative profiling small RNA populations in different plant species, for which limited genome information is available, such as *M*. *oleifera*.

### Sequence data analysis

Several bioinformatics tools have been developed to identify conserved miRNAs and discover new ones, starting from high-throughput sequencing [24–27]. The biggest limitation of these algorithms is that genome sequencing is required to execute the analysis. The only published algorithm able to discover novel miRNAs in species without sequenced genome is miReader [28]. Jha and coworkers also presented an approach for identifying novel miRNAs in *Miscanthus giganteus*, whose genome has not been sequenced yet [28]. Although the *M*. *oleifera* genome was already sequenced in 2015 [7], the raw data are not yet available, therefore we proceeded with a genome-independent strategy based on Jha’s work.

S1 Fig summarizes the analysis workflow performed with small RNA raw data. This analysis was performed to identify a pool of RNA fragments with lengths between 18 and 24 nucleotides that would be added to the subsets of known or uncharacterized miRNAs. These data have been deposited in NCBI/GEO public database under accession number GSE70423.

#### Raw data filtering

Starting from a total of 31,290,964 reads the Illumina small-RNA adapter was first clipped using FASTX-Toolkit (http://hannonlab.cshl.edu/fastx_toolkit). Sequences shorter than 15nt after trimming were discarded and we obtained a total of 22,737,895 reads. Sequencing artifacts were removed and the remaining reads were trimmed on the 3p and 5p ends in order to remove low-quality bases. At the end of this procedure BLASTn [29] v 2.2.30 with parameters “-task megablast, -perc_identity 100” was used to compare the remaining reads with the Rfam database (Rfam 11.0) [30]. This stringent step removed non-coding RNA (rRNA, tRNA, snRNA, snoRNA) and degraded fragments of mRNA. Reads with lengths less than 15 nucleotides were discarded. After the alignment process, a total of 10,415,180 reads were identified as possible miRNA sequences.

#### Identification of known and putative novel miRNAs

The 10,415,180 reads highlighted as possible miRNAs were aligned against a specific-plant miRBASE (Release 21) with a low-redundancy rate (http://www.mirbase.org/) using BLASTn v 2.2.30 [29] with parameters “-task blastn-short, -perc_identity 100”. Sequences with homology rate equal to 100% were considered conserved *M*. *oleifera* miRNAs. In the alignment process all reads with abundance values lower than 10 copies were discarded resulting in a total of 303,872 reads.

In order to identify uncharacterized putative novel miRNAs, all the discarded reads, generated from the alignment with the wide libraries of miRNAs present in the repertory of miRBase bank, have been selected by a homemade custom script and were considered as candidate novel miRNAs.

Subsequently, the miReader algorithm [28] has been used to identify a strict number of uncharacterized miRNAs without the support of a reference genome. Although, this algorithm is less qualitatively informative (i.e. prediction of secondary structures) than others algorithms, it can be considered the most effective quantitative analysis with unknown genome, as is the case of *M*. *oleifera*.

#### Quality controls

Quality controls were performed using FastQC software [23], before (S1 File) and after (S1 File) removing the sequencing adapter. Figures A and B in S1 File show the quality scores across all bases and sequences in more detail, highlighting the validity of sequencing and analysis processes. The Illumina small-RNA adapter inside the sequenced sample is clearly visible. (Figure C in S1 File). In Figures A and B in S2 File is visible how sequencing quality was preserved through the filtering process, the complete removal of the small-RNA adapter (Figure C in S2 File) and the removal of all ncRNAs that do not belong to miRNAs.

#### Predicting conserved miRNAs across other plant species

In order to evaluate the conservation rate for *M*. *oleifera* miRNAs across all others plant species, we developed a custom script, which retrieves the sequence information for plant organisms stored inside MirBase repository and evaluates the overlapping rate in terms of sequence homology between *M*. *oleifera* and all other plant miRNAs.

### Target prediction

#### Predicting mRNA targets in plants for both known and uncharacterized miRNAs

S2 Fig shows the process to predict gene target for known and novel-putative miRNA. Starting from the list of 74 known, psRNATarget [31] was used to predict target genes in the plant. To reduce the false-positive rate we set the maximum expectation value to 2.0 and forced the system to select only the best five predictions for each miRNA. After the target prediction analysis, the three gene sets were submitted to PlantGSEA [32] to obtain a smaller number of gene clusters that are related to plant molecular processes.

#### Predicting known miRNA with putative roles in human gene regulation

Several *Moringa* miRNAs were further analyzed to investigate their possible roles in human gene regulation. MirCompare (http://160.80.35.140/MirCompare) is a web-application based on an algorithm that compares libraries of miRNAs belonging to organisms from plant and animal kingdoms in order to find cross-kingdom regulation. The main settings for MirCompare analysis are: 1) r-value: the best comparison rate between each miRNA couple; 2) seed-region stringency: the minimum number of matches, related to the seed region.

MirCompare was used with r-value = 0.55 and seed-region threshold = 5 to identify possible *Moringa* miRNAs with functional homologies to mammalian miRNAs. The combinatorial miRNA target prediction (COMIR) web tool [33], which combines four popular scoring schemes (miRanda, PITA, TargetScan and mirSVR), was used to compute the potential of a gene to be targeted by a set of miRNAs. Therefore, COMIR tool generated a list of genes that might be regulated by *M*. *oleifera* miRNAs. Subsequently, the identified genes were submitted to Gene Ontology enrichment analysis to understand the pathways, in which miRNAs might putatively play a regulating role.

#### Transfection

We studied biological activity of the synthetic *mol-miR168a* (UCGCUUGGUGCAGGUCGGGAC) that was transfected into the hepatocellular carcinoma cell line (HEP-G2) by the lipofectamin method (Hi-Fect, Qiagen, USA) according with manufacture’s instruction (MiRNA mimic and inhibitor experiments protocols®, Qiagen, USA). Synthetic *mol-miR168a* tagged with fluorescein isothiocyanate (FITC) was used also as control of transfection. Cells were harvested 72h after transfection and characterized for the efficiency of miRNA uptake by EVOS FLoid cell imaging station (LifeTechnologies, USA) analysis, and for the effect of miRNAs on specific target genes by Western Blot assay.

#### Western blot analysis

Aliquots of 3x10^6^ transfected cells were suspended in buffers for cytoplasmic or nuclear protein extraction, and further processed for western blot analysis as already described [34]. Primary antibodies included the rabbit monoclonal antibody *SIRT1* and the goat monoclonal antibody human *beta-actin* (Santa Cruz biotechnology, CA, USA). Secondary antibodies included anti-goat and anti-rabbit IgG chain-specific antibodies that were conjugated to peroxidase (Calbiochem, Merck Millipore, Darmstadt, Germany). Western blot analysis for each sample was quantified by densitometry analysis (TINA software)

Data analysis was performed using GraphPad Prism version 6.00 (GraphPad Software, La Jolla, CA, USA). Statistical probabilities were expressed as p <0.05 (*) or p < 0.01 (**). Comparison of means of *SIRT-1* protein expression in response to *mol-miR168a* transfection was carried out using t-Test analysis.

### Experimental validation of conserved miRNAs by Quantitative Real-Time PCR (qRT-PCR)

The expression level of different miRNAs was validated using poly(A)-tailed qRT-PCR method. According to the manufacturer’s protocols (Exiqon A/S, Vedbaek, Denmark), a poly-A tail was added to the mature miRNA templates (20 ng). cDNA was synthesized using a poly-T primer with a 3’ degenerate anchor and a 5’ universal tag at 42°C for 60 min followed by heat-inactivated for 5 min at 95°C. To provide a control for quality of the cDNA synthesis reaction and the PCR, RNA spike-in (*UniSp6*) was added to the sample prior to cDNA synthesis. The cDNA template was then amplified using miRNA-specific and LNA™-enhanced forward and reverse primers. SYBR®Green was used for detection. The reactions were carried out in a Rotor-Gene®Q 72-Well Rotor (Qiagen, USA) with the following amplification conditions: activation/denaturation at 95°C for 10 min followed by 40 cycles of denaturation at 95°C for 10s, annealing and extension together at 60°C for 60 sec. Finally, melting analyses were performed to confirm the absence of false-positive peaks. qRT-PCR was performed only for conserved miRNAs. All reactions were performed in triplicate for each sample. Two controls (no template control and no Reverse Transcription control) were included in all reactions. Relative expression levels of miRNAs were quantified by using the 2^-Δ ΔCt^ method and *miR159* was used as the internal control miRNA. To determine significant differences among samples or miRNAs we applied a One Way ANOVA analysis using GraphPad Prism version 6.00.

## Results

### Analyzing small RNA populations

**Fig 1:**
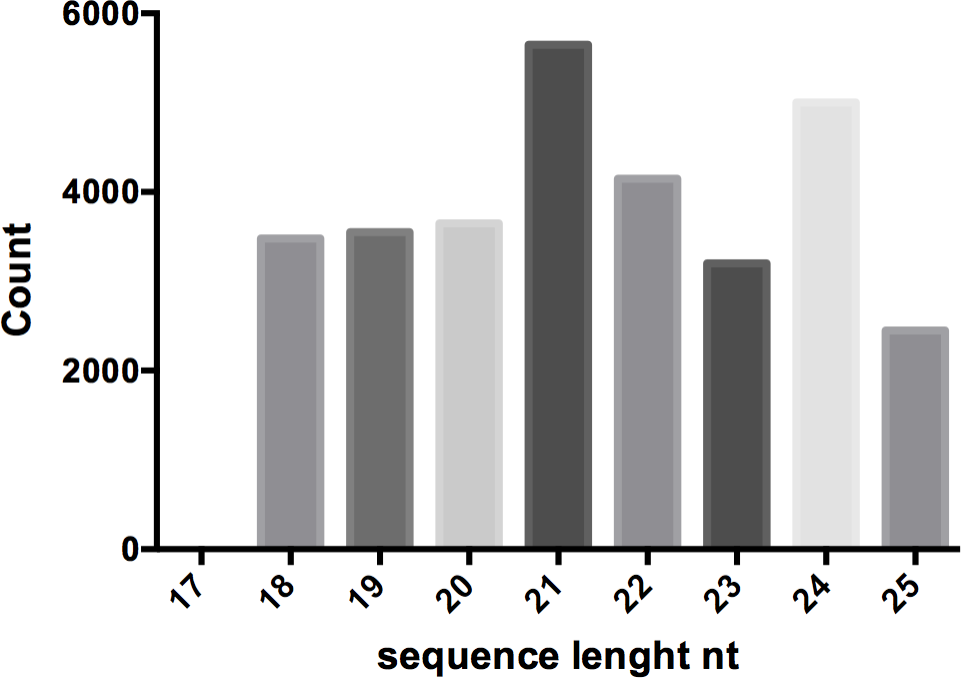
Length distribution of small RNA populations. Sequence length distributions of sRNAs in *M*. *oleifera* seeds filtered by RFAM database.

sRNAs from *M*. *oleifera* seeds with 5’-phosphate and 3’-hydroxyl groups were identified by high-throughput Illumina TruSeq smallRNA sequencing (Illumina HiSeq 1500). cDNA libraries were constructed from seeds of *M*. *oleifera* plants after removing the 5 bp adapter sequence and filtering out low quality ‘‘n” sequences. One small RNA library was constructed with about 30 million reads. Reads cleaned from adapters, ranging from 14 to 51 nts in length, were filtered with the Rfam database [30]. Looking at the sequence distribution after the filtering process, the majority of reads were 18 to 25 nts in length. The main size groups were 21 nt and 24 nt, respectively (Fig 1). These results were consistent with previous studies in other plant species where 24 nt small RNAs were the most abundant [19]. The amounts of 21 nt and 24 nt small RNAs were approximately 18.15% and 16.06%, respectively.

### Identifying conserved *M*. *oleifera*-specific miRNAs

Conserved families of miRNAs are present in many plant species due to their important regulatory role played. The highly conserved nature gives the opportunity to identify miRNAs in plant species for which genome sequence information is partially or fully available, or previously undefined. To identify the conserved miRNAs in *M*. *oleifera* seed plants, the massive dataset was compared to known plant mature miRNAs against miRBASE (Release 21), using BLASTn. The sequence analysis revealed the presence of 94 miRNAs belonging to 40 conserved families (Table 1). Deeper investigations conducted by miRBASE also identify secondary structures for most of the known *M*. *oleifera* miRNA sequences (S1 Table).

**Table 1:** Known microRNAs in *M*. *oleifera* seeds.

miRNA family	miRNA members	miRNA sequence	miRNA* sequence	Read Counts	
				miR	miR*
*mol-miR156*	*mol-miR156*	CUGACAGAAGAGAGUGAGCAC		2459	
	*mol-miR156d*		GCUCUCUAUGCUUCUGUCAUCA		23
	*mol-miR156f*	CUGACAGAAGAGAGUGAGCA	CUCACUUCUCUUUCUGUCAAUC	86	34
	*mol-miR156g*	CGACAGAAGAGAGUGAGCAC		77	
	*mol-miR156h*	UGACAGAAGAAAGAGAGCAC	GCUCUCUUUCCUUCUGCCACC	33	NA
	*mol-miR156j*	GUUGACAGAAGAGAGUGAGCAC		2546	
	*mol-miR156q*	UGACAGAAGAGAGUGAGCACU		2476	
	*mol-miR156t*	UUGACAGAAGAGAGAGAGCAC		111	
*mol-miR157*	*mol-miR157a*	UUGACAGAAGAUAGAGAGCAC	GCUCUCUAGCCUUCUGUCAUCA	316	NA
	*mol-miR157d*	UGACAGAAGAUAGAGAGCAC	GCUCUCUAUGCUUCUGUCAUC	NA	23
*mol-miR159*	*mol-miR159*	AGCUCCCUUCGAUCCAAUC	CUUGGAUUGAAGGGAGCUCU	NA	48
	*mol-miR159a*	UUUGGAUUGAAGGGAGCUCUA		11908	
	*mol-miR159b*		UUUGGAUUGAAGGGAGCUCUU		2555
	*mol-miR159b*.*1*		UUUGGAUUGAAGGGAGCUCUG		2492
	*mol-miR159c*		UUUGGAUUGAAGGGAGCUCCU		1917
	*mol-miR159d*	AUUGGAUUGAAGGGAGCUCCG		21	
	*mol-miR159f*	CUUGGAUUGAAGGGAGCUCUA		442	
*mol-miR160*	*mol-miR160h*	UGCCUGGCUCCCUGUAUGCCAUU		25	
*mol-miR162*	*mol-miR162*		UCGAUAAACCUCUGCAUCCAG		158
	*mol-miR162a*	UCGAUAAACCUCUGCAUCCA		16	
*mol-miR164*	*mol-miR164a*	UGGAGAAGCAGGGCACGUGAA		13	
	*mol-miR164c*	UGGAGAAGCAGGGCACGUGCG		13	
	*mol-miR164d*	UGGAGAAGCAGGGCACGUGCA		373	
*mol-miR165*	*mol-miR165a*	UCGGACCAGGCUUCAUCCCCC		368	
*mol-miR166*	*mol-miR166*	CCGGACCAGGCUUCAUCCCAG		14	
	*mol-miR166b*	UCGGACCAGGCUUCAUUCCCUU		2367	
	*mol-miR166e*	GGACCAGGCUUCAUUCCCC		5541	
	*mol-miR166h*	UCGGACCAGGCUUCAUUCCCGU		2923	
	*mol-miR166i*	UCGGACCAGGCUUCAUUCCCCC		65243	
	*mol-miR166j*	UCCGGACCAGGCUUCAUUCCC		546	
	*mol-miR166k*	GGAUUGUUGUCUGGCUCGGUG	UCGGACCAGGCUUCAAUCCCU	NA	240
	*mol-miR166u*	UCUCGGACCAGGCUUCAUUC		3738	
*mol-miR167*	*mol-miR167*	UCAAGCUGCCAGCAUGAUCUA	AGAUCAUGUGGCAGUUUCACC	27	514
	*mol-miR167a*	UGAAGCUGCCAGCAUGAUCUC		3881	
	*mol-miR167b*	UGAAGCUGCCAGCAUGAUCUA		3834	
	*mol-miR167c*	UGAAGCUGCCAGCAUGAUCUGG		3908	
	*mol-miR167c*	UAAGCUGCCAGCAUGAUCUUG	UAGGUCAUGCUGGUAGUUUCACC	271	NA
	*mol-miR167d*	UGAAGCUGCCAGCAUGAUCUGA		4007	
	*mol-miR167h*	UGAAGCUGCCAGCAUGAUCUUA	AGAUCAUGUGGCAGUUUCACC	18563	NA
	*mol-miR167i*	UCAUGCUGGCAGCUUCAACUGGU		586	
*mol-miR168*	*mol-miR168*	AUUCAGUUGAUGCAAGGCGGGAUC		91	
	*mol-miR168a*	UCGCUUGGUGCAGGUCGGGAC		165	
	*mol-miR168c*	UCGCUUGGUGCAGGUCGGGAC	CCCGCCUUGCAUCAACUGAAU	NA	91
	*mol-miR168d*	UCGCUUGGUGCAGGUCGGGAA	CCCGCCUUGCAUCAACUGAAU	517	NA
*mol-miR169*	*mol-miR169d*	UAGCCAAGGAUGACUUGCCU		22	
*mol-miR170*	*mol-miR170*	UAUUGGCCUGGUUCACUCAGA	UGAUUGAGCCGUGUCAAUAUC	NA	77
*mol-miR171*	*mol-miR171a*	UGAUUGAGCCGUGCCAAUAU		423	
	*mol-miR171c*	UAUUGACGCGGUUCAAUUCGA	UGAUUGAGCCGUGCCAAUAUC	NA	503
	*mol-miR171d*		UUGAGCCGUGCCAAUAUCACG		410
*mol-miR172*	*mol-miR172m*	GGAGCAUCAUCAAGAUUCACA	AGAAUCUUGAUGAUGCUGCAG	NA	68
*mol-miR319*	*mol-miR319*	UUGGACUGAAGGGAGCUCCC		1204	
	*mol-miR319e*	UUUGGACUGAAGGGAGCUCCU		2843	
	*mol-miR319g*	UUGGACUGAAGGGAGCUCCUUC		1240	
*mol-miR390*	*mol-miR390a*	AAGCUCAGGAGGGAUAGCGCC	CGCUAUCCAUCCUGAGUUUCA	1128	82
	*mol-miR390d*	AAGCUCAGGAGGGAUAGCGCC	CGCUAUCCAUCCUGAGUUUUA	NA	15
	*mol-miR390e*	AGCUCAGGAGGGAUAGCGCC	CGCUAUCUAUCCUGAGCUCCA	203	NA
*mol-miR393*	*mol-miR393a*	CAUCCAAAGGGAUCGCAUUGA		591	
	*mol-miR393b*	UCCAAAGGGAUCGCAUUGAUC		1730	
	*mol-miR393c*	UCCAAAGGGAUCGCAUUGAUCU	AUCAGUGCAAUCCCUUUGGAAU	11813	NA
	*mol-miR393h*	UUCCAAAGGGAUCGCAUUGAUC		9974	
*mol-miR394*	*mol-miR394b*	UUGGCAUUCUGUCCACCUCC	CUGUUGGUCUCUCUUUGUAA	1147	NA
*mol-miR395*	*mol-miR395a*	CUGAAGUGUUUGGGGGAACUC		84	
	*mol-miR395d*	UGAAGUGUUUGGGGGAACUUU		26	
	*mol-miR395g*	UUGAAGUGUUUGGGGGAACUC		43	
	*mol-miR395h*	AUGAAGUGUUUGGGGGAACUU		26	
*mol-miR396*	*mol-miR396a*	UUCCACAGCUUUCUUGAACGU		265	
	*mol-miR396c*	UUCCACAGCUUUCUUGAACUU		7917	
	*mol-miR396e*	UUCCACAGGCUUUCUUGAACUG		188	
	*mol-miR396g*	UCCCACAGCUUUAUUGAACUG	GUUCAAGAAAGCUGUGGAAGA	12	265
	*mol-miR396h*	UCCACAGCUUUCUUGAACUG		419	
*mol-miR397*	*mol-miR397a*	UCAUUGAGUGCAGCGUUGAUG		529	
*mol-miR398*	*mol-miR398a*	GGGUUGAUUUGAGAACAUAUG	UAUGUUCUCAGGUCGCCCCUG	NA	23
	*mol-miR398c*	UGUGUUCUCAGGUCGCCCCUG		3285	
	*mol-miR398f*	GGUGUUCUCAGGUCGCCCCUG		115	
*mol-miR399*	*mol-miR399a*	CGCCAAAGGAGAGUUGCCCUU		119	
	*mol-miR399d*	UGCCAAAGGAGAGUUGCCCUU		76	
*mol-miR403*	*mol-miR403*	UUAGAUUCACGCACAAACUCG		5277	
	*mol-miR403a*	UUAGAUUCACGCACAAACUUG		324	
*mol-miR408*	*mol-miR408*	CAGGGAUGAGGCAGAGCAUGG	CUGCACUGCCUCUUCCCUGGC	NA	27
*mol-miR530*	*mol-miR530*	UCUGCAUUUGCACCUGCACCU		950	
*mol-miR535*	*mol-miR535a*	UGACAACGAGAGAGAGCACGC		347	
*mol-miR827*	*mol-miR827*	UUUGUUGAUUGACAUCUAUAC	UUAGAUGACCAUCAACAAACG	22	NA
*mol-miR858*	*mol-miR858b*	UUCGUUGUCUGUUCGACCUUG		34	
*mol-miR894*	*mol-miR894*	CGUUUCACGUCGGGUUCACC		396	
*mol-miR1310*	*mol-miR1310*	AGGCAUCGGGGGCGCAACGCCC		1170	
*mol-miR1511*	*mol-miR1511*	CGUGGUAUCAGAGUCAUGUUA	ACCUGGCUCUGAUACCAUAAC	1279	NA
*mol-miR1515*	*mol-miR1515*	UCAUUUUUGCGUGCAAUGAUCC		61	
*mol-miR3711*	*mol-miR3711*	UGGCGCUAGAAGGAGGGCCU		271	
*mol-miR5139*	*mol-miR5139*	AAACCUGGCUCUGAUACCA		1612	
*mol-miR5559*	*mol-miR5559*	UACUUGGUGAAUUGUUGGAUC		322	
*mol-miR6300*	*mol-miR6300*	GUCGUUGUAGUAUAGUGG		10523	
*mol-miR6478*	*mol-miR6478*	CCGACCUUAGCUCAGUUGGUG		8520	
*mol-miR7751*	*mol-miR7751*	AUCUUCCUCGUGGACAAGCGGUAG	UUUGGUGCACCCGGCUGGAGAUGG	NA	1683
*mol-miR8155*	*mol-miR8155*	UAACCUGGCUCUGAUACCA		1612	

**Fig 2:**
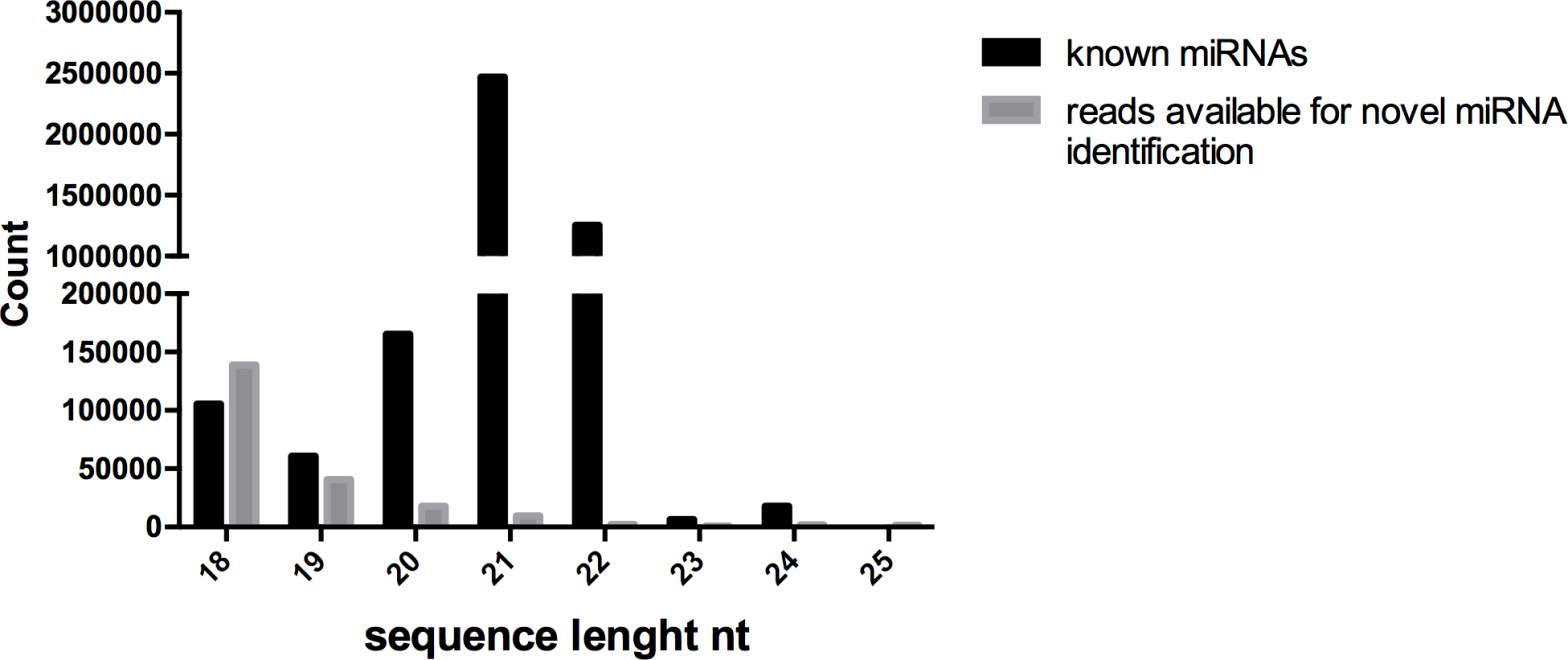
Length distribution of miRNA populations. Sequence length distributions of known and unknown miRNAs in *M*. *oleifera* seeds.

The most abundant miRNA families were *miR166*, *miR156* and *miR167* with eight members each. Among the other families, in *miR159*, seven members, and in *miR396* five members were present. The remaining miRNA families had less than five members. As reported in Fig 2, the sequence length distribution had two major peaks at 21 and 22nts with high levels of reads from 20 to 22 nucleotides. Low levels of 24 nucleotide fragments could be due to the high stringent filters applied.

### Predicting uncharacterized *M*. *oleifera* miRNAs

All data set was analysed by BLASTn and 21,065 sequences were identified as available for novel miRNA identification with miReader software. To avoid spurious contamination of mRNA fragments or sequencing artifacts, this filtering process was forced to discard all reads shorter than 18nts nucleotides. The length distribution of the resulted sequences is shown in Fig 2. The distribution had a peak at 18 nts decreasing as the sequence length increases. The processed set of sequences was sub-sequentially provided to miReader and, after the analysis, only two uncharacterized miRNA duplexes were predicted as novel uncharacterized miRNAs (Table 2). This result was not surprising since only the non-conserved miRNAs are normally expressed at low level, only in specific cell-types or under specific organ development and growth conditions [35].

**Table 2:** Putative novel miRNAs list.

miRNA members	miRNA sequence	miRNA* sequence	Read Counts	
			miR	miR*
*mol-miR1p*	CCGUCUCGCCCGGACCCUG	CGACGCGGAUCGCGACGG	12	99
*mol-miR2p*	CUAUACCCGGCCGUUGGGGC	ACCGCAUAGCGCAGUGGAU	129	53

### Identifying known miRNA conservation rates among plant species

In order to evaluate the conservation rate for the *M*. *oleifera* known miRNAs within the plant kingdom an in-house ruby script was used. In Table 3, the top 20 conserved miRNAs together with their conservation rates and their abundance in *M*. *oleifera* seeds are reported.

**Table 3:** Most highly conserved miRNAs among selected plants.

miRNA name	Sequence	Conservation Rate	Read Counts
*mol-miR166i*	UCGGACCAGGCUUCAUUCCCCC	147	65,243
*mol-miR156*	CUGACAGAAGAGAGUGAGCAC	136	2,459
*mol-miR160h*	UGCCUGGCUCCCUGUAUGCCAUU	91	25
*mol-miR395a*	CUGAAGUGUUUGGGGGAACUC	90	84
*mol-miR171c-3p*	UGAUUGAGCCGUGCCAAUAUC	77	503
*mol-miR164d*	UGGAGAAGCAGGGCACGUGCA	73	373
*mol-miR167b*	UGAAGCUGCCAGCAUGAUCUA	66	3,834
*mol-miR157a-5p*	UUGACAGAAGAUAGAGAGCAC	63	316
*mol-miR390a-5p*	AAGCUCAGGAGGGAUAGCGCC	54	1,128
*mol-miR169d*	UAGCCAAGGAUGACUUGCCU	51	22
*mol-miR394b-5p*	UUGGCAUUCUGUCCACCUCC	44	1,147
*mol-miR396c*	UUCCACAGCUUUCUUGAACUU	40	7,917
*mol-miR167d*	UGAAGCUGCCAGCAUGAUCUGA	38	4.007
*mol-miR162*	UCGAUAAACCUCUGCAUCCAG	34	158
*mol-miR319e*	UUUGGACUGAAGGGAGCUCCU	33	2,843
*mol-miR403*	UUAGAUUCACGCACAAACUCG	31	5,277
*mol-miR171d*	UUGAGCCGUGCCAAUAUCACG	30	410
*mol-miR168d-5p*	UCGCUUGGUGCAGGUCGGGAA	25	517
*mol-miR393b*	UCCAAAGGGAUCGCAUUGAUC	24	1,730
*mol-miR159a*	UUUGGAUUGAAGGGAGCUCUA	23	11,908

**Fig 3:**
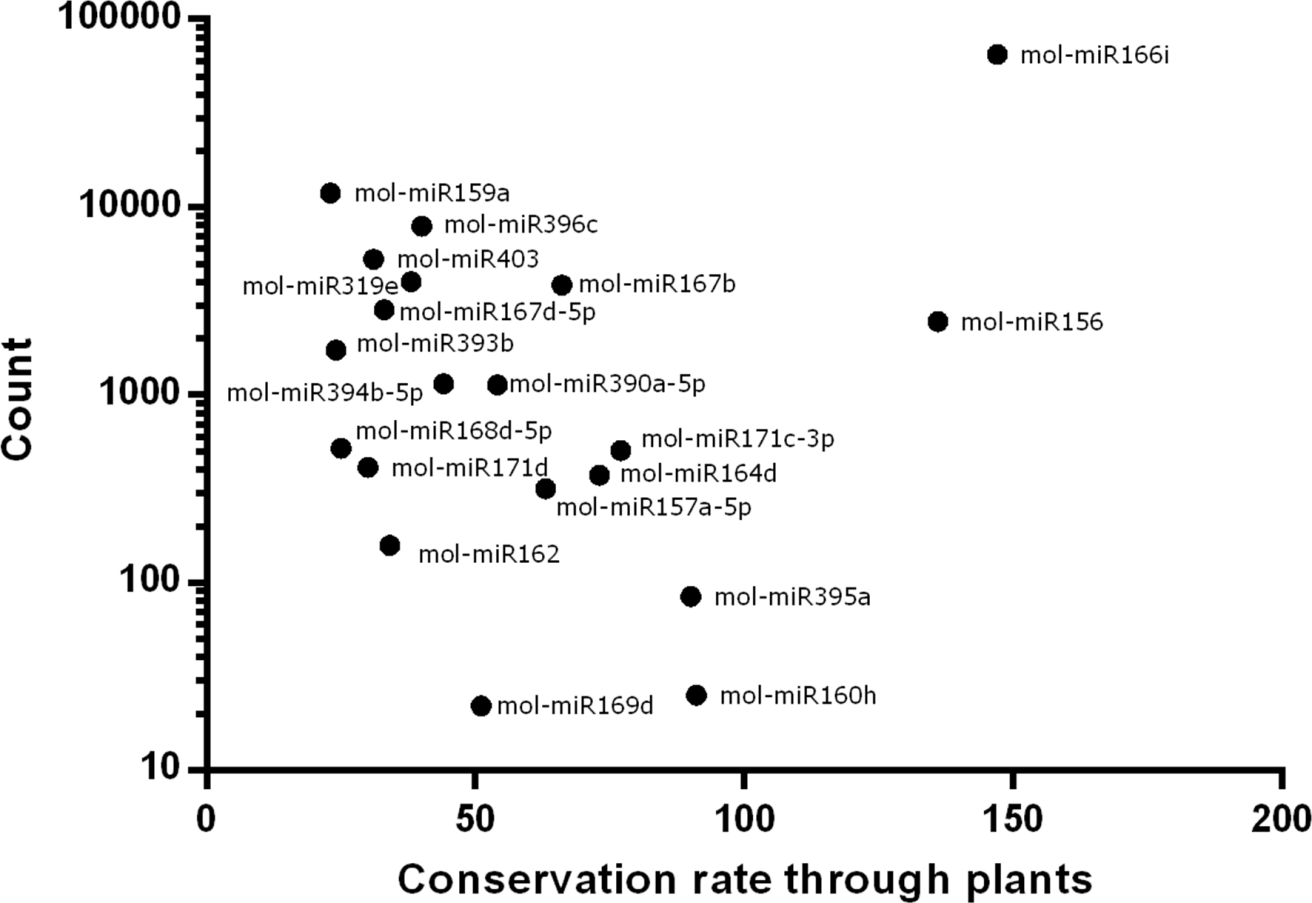
Correlation analysis. Correlation analysis between the conservation rate through plants and abundance in *M*. *oleifera* samples.

Moreover, we drew a plot for better understanding the relationship between miRNA conservation rate and abundance in the seed tissues of *Moringa*. A logarithmic scale was used to obtain a linear plot since the range of abundance values was too high. As shown in Fig 3, *mol-miR166i* was the most conserved miRNA throughout plant species and the most abundant miRNA in *Moringa* seed tissues.

### Validating expression of conserved miRNAs among the organ of *M*. *oleifera* and crop plants species

**Fig 4:**
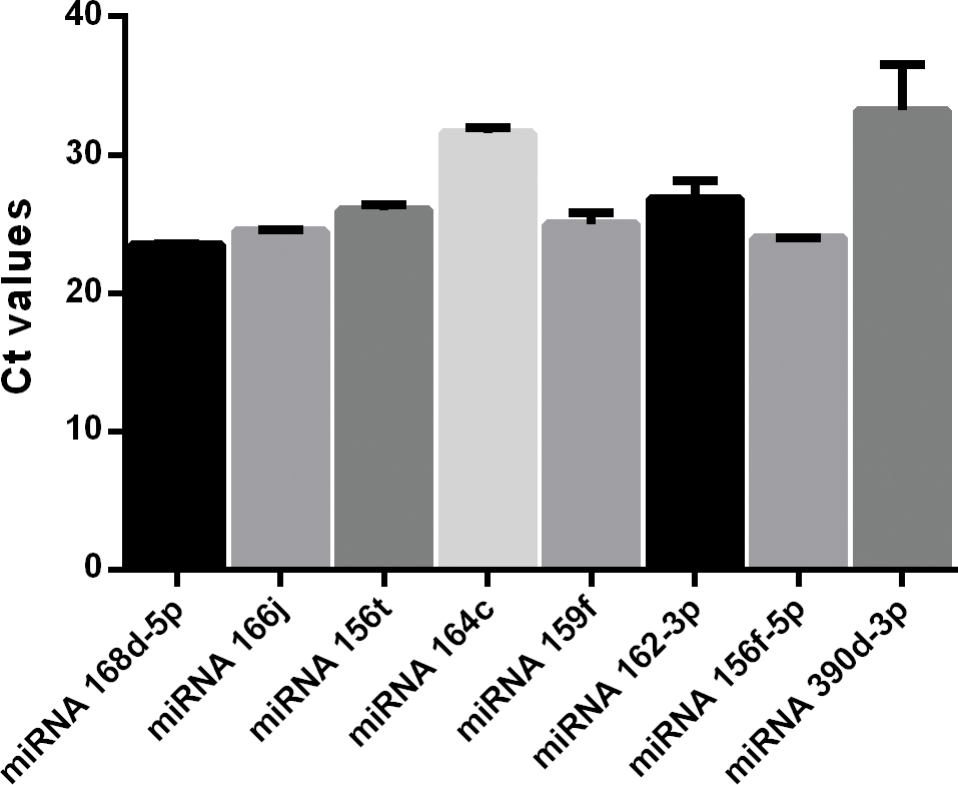
Quantitative RT-PCR analysis of mature miRNAs in *M*. *oleifera* seeds. A quantitative analysis of plant miRNAs in *M*. *oleifera* seeds by qRT-PCR. The levels of 8 plant miRNAs detected by Illumina sequencing and confirmed by qRT-PCR. All qRT-PCR reactions were prepared in triplicate for each sample.

To validate the representative expression patterns of the miRNAs, we performed qRT-PCR analysis of eight conserved miRNAs on different organ tissues of *M*. *oleifera* and on three crop plant species. The data collected demonstrated that the expression patterns were similar between the two analytical tools (Illumina sequencing and qRT-PCR) for six of the eight miRNAs, whereas *mol-miR156f-5p* showed different expression pattern as detected between the two molecular tools (Fig 4).

**Fig 5:**
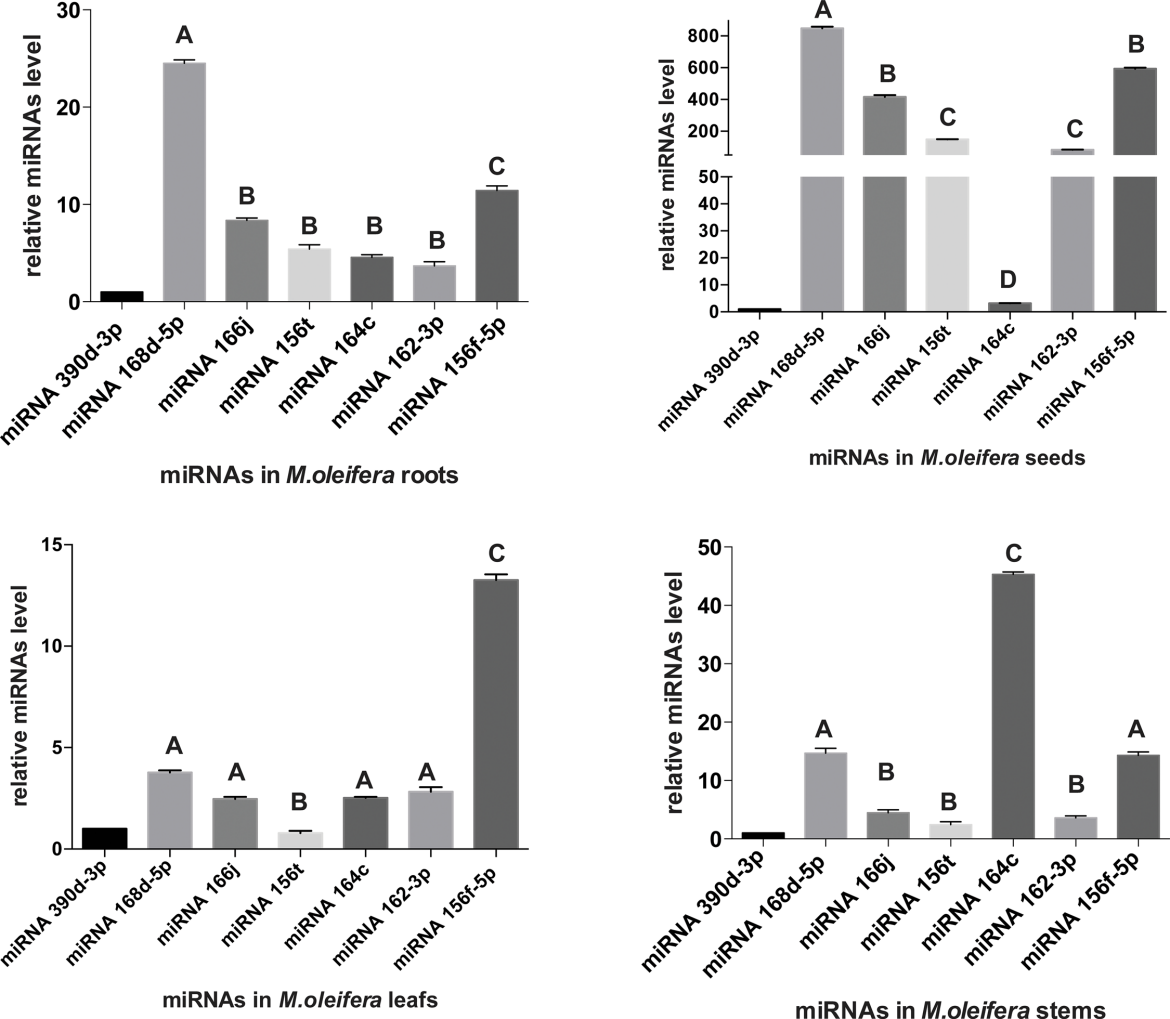
Quantitative RT-PCR analysis of mature miRNAs in *M*. *oleifera* different tissues. The relative levels of 6 plant miRNAs detected by qRT-PCR in different *M*. *oleifera* tissues. The expression level of *miR390d-3p* was set as control and taken as 1, and expression level in all other miRNAs was quantified relative to it. *MiR159* was used as an endogenous control. Each value represents the mean of three different determination. Significant differences at P<0.05 (One Way ANOVA) between miRNAs are indicated with different letters.

**Fig 6:**
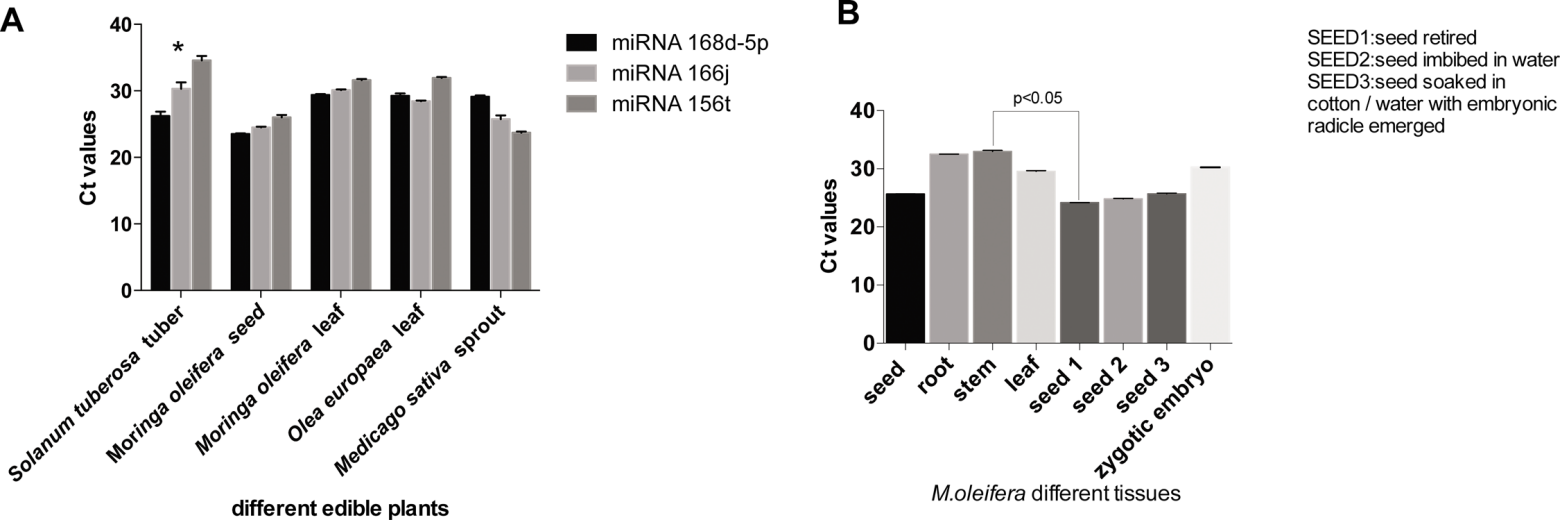
Quantitative RT-PCR analysis of mature miRNAs in different edible plants and in different *M*. *oleifera* tissues. (A) Quantitative analysis of plant *mol-miR168d-5p*, *mol-miR166j* and *mol-miR156t* in different edible plants. (B) Quantitative analysis of *mol-miR156t* in different tissue and in different seed conditions. Data are reported as the mean of three different experiments. Significant differences at P<0.05 (One Way ANOVA) between samples are indicated with asterisks or line.

As illustrated in Fig 5, *mol-miR168d-5p* and *mol-miR156f-5p* were more abundant than other miRNAs in seed, leaf and root tissues. Vice versa, *mol-miR164c* showed higher expression levels in stem tissues. However, apart from *mol-miR164c* other miRNAs detected by qRT-PCR had higher expression in seeds compared to the other organ tissues. Similar results were obtained by assessing conserved *mol-miR168d-5p*, *mol-miR166j* and *mol-miR156t* in multiple plant tissues. As shown (Fig 6A) *mol-miR168d-5p* was the most abundant among well-expressed miRNAs, except for *M*. *sativa* sprout and *O*. *europaea* leaf. Moreover, when comparing *mol-miR156t* expression levels in different tissues, including zygotic embryo, and in seeds treated in different way, we found that it was mainly expressed in dry seed tissues rather than other tissues (Fig 6B).

### Predicting target genes for conserved and uncharacterized miRNAs

The system of prediction analysis was forced to select the five best predictions for each miRNA, and from the resulting data, 48 putative target genes were identified as potentially regulated by known miRNAs (Table 4).

**Table 4:** List of target genes for known *M*. *oleifera* miRNAs.

miRNA name	Target Gene	Entrez ID	Gene Aligned Sequence	Mechanism of regulation
*mol-miR393c-5p*	*AFB3*	837838	AACAAUGCGAUCCCUUUGGA	Cleavage
*mol-miR393c-5p*	*AFB2*	822296	AACAAUGCGAUCCCUUUGGA	Cleavage
*mol-miR156*	*SPL3*	817948	UGCUUACUCUCUUCUGUCAG	Cleavage
*mol-miR156q*	*SPL10*	839626	AGUGCUCUCUCUCUUCUGUCA	Cleavage
*mol-miR156j*	*SPL11*	839625	GUGCUCUCUCUCUUCUGUCAAC	Cleavage
*mol-miR156j*	*SPL10*	839626	GUGCUCUCUCUCUUCUGUCAAC	Cleavage
*mol-miR159a*	*MYB101*	817807	UAGAGCUUCCUUCAAACCAAA	Cleavage
*mol-miR159a*	*DUO1*	825217	UGGAGCUCCAUUCGAUCCAAA	Cleavage
*mol-miR159a*	*AtMYB104*	817236	UGGAGCUCCCUUCAUUCCAAG	Cleavage
*mol-miR397a*	*LAC2*	817462	AUCAAUGCUGCACUCAAUGA	Cleavage
*mol-miR397a*	*IRX12*	818386	GUCAACGCUGCACUUAAUGA	Cleavage
*mol-miR397a*	*LAC17*	836124	AUCAAUGCUGCACUUAAUGA	Cleavage
*mol-miR166j*	*PHV*	839928	GGGAUGAAGCCUGGUCCGGA	Cleavage
*mol-miR393a*	*AFB3*	837838	CAAUGCGAUCCCUUUGGAUG	Cleavage
*mol-miR393a*	*TIR1*	825473	CAAUGCGAUCCCUUUGGAUG	Cleavage
*mol-miR393a*	*AFB2*	822296	CAAUGCGAUCCCUUUGGAUG	Cleavage
*mol-miR393a*	*GRH1*	828045	CCAUGCGAUCCCUUUGGAUG	Cleavage
*mol-miR171a*	*HAM1*	836582	AUAUUGGCGCGGCUCAAUCA	Cleavage
*mol-miR171a*	*HAM2*	830834	AUAUUGGCGCGGCUCAAUCA	Cleavage
*mol-miR171a*	*HAM3*	828208	AUAUUGGCGCGGCUCAAUCA	Cleavage
*mol-miR156t*	*SPL10*	839626	GUGCUCUCUCUCUUCUGUCAA	Cleavage
*mol-miR156t*	*SPL2*	834345	GUGCUCUCUCUCUUCUGUCAA	Cleavage
*mol-miR396e*	*AtGRF4*	824457	CCGUUCAAGAAAGCCUGUGGAA	Cleavage
*mol-miR396e*	*AtGRF3*	818213	CCGUUCAAGAAAGCCUGUGGAA	Cleavage
*mol-miR396e*	*AtGRF1*	816815	GUUCAAGAAAGCCUGUGGAA	Cleavage
*mol-miR396e*	*AtGRF2*	829930	GUUCAAGAAAGCCUGUGGAA	Cleavage
*mol-miR396e*	*AtGRF9*	819156	GUUCAAGAAAGCUUGUGGAA	Cleavage
*mol-miR172m-3p*	*AP2*	829845	CUGCAGCAUCAUCAGGAUUCU	Cleavage
*mol-miR172m-3p*	*TOE2*	836134	UGCAGCAUCAUCAGGAUUCU	Cleavage
*mol-miR156h-5p*	*SPL10*	839626	GUGCUCUCUCUCUUCUGUCA	Translation
*mol-miR156h-5p*	*SPL13A*	21393429	GUGCUCUCUCUCUUCUGUCA	Translation
*mol-miR156h-5p*	*SPL2*	834345	GUGCUCUCUCUCUUCUGUCA	Translation
*mol-miR156f-5p*	*SPL3*	817948	UGCUUACUCUCUUCUGUCAG	Cleavage
*mol-miR160h*	*ARF17*	844120	UGGCAUGCAGGGAGCCAGGCA	Cleavage
*mol-miR160h*	*ARF10*	817382	GGAAUACAGGGAGCCAGGCA	Cleavage
*mol-miR827-5p*	*NLA*	839559	UGUUUGUUGAUGGUCAUCUAA	Cleavage
*mol-miR395g*	*AST68*	830882	AGUUCUCCCAAACACUUCAA	Cleavage
*mol-miR395h*	*APS4*	834400	GAGUUCCUCCAAACACUUCAU	Cleavage
*mol-miR395d*	*APS4*	834400	AGAGUUCCUCCAAACACUUCA	Cleavage
*mol-miR395d*	*AST68*	830882	AAGUUCUCCCAAACACUUCA	Cleavage
*mol-miR166*	*PHV*	839928	UUGGGAUGAAGCCUGGUCCGG	Cleavage
*mol-miR166*	*PHB*	818036	UUGGGAUGAAGCCUGGUCCGG	Cleavage
*mol-miR157a-5p*	*SPL10*	839626	GUGCUCUCUCUCUUCUGUCA	Translation
*mol-miR157a-5p*	*SPL13A*	21393429	GUGCUCUCUCUCUUCUGUCA	Translation
*mol-miR157a-5p*	*SPL2*	834345	GUGCUCUCUCUCUUCUGUCA	Translation
*mol-miR164c*	*CUC1*	820748	GCACGUGUCCUGUUUCUCCA	Cleavage
*mol-miR164c*	*CUC2*	835478	GCACGUGUCCUGUUUCUCCA	Cleavage
*mol-miR164a*	*ANAC080*	830661	UUUACGUGCCCUGCUUCUCCA	Cleavage

**Fig 7:**
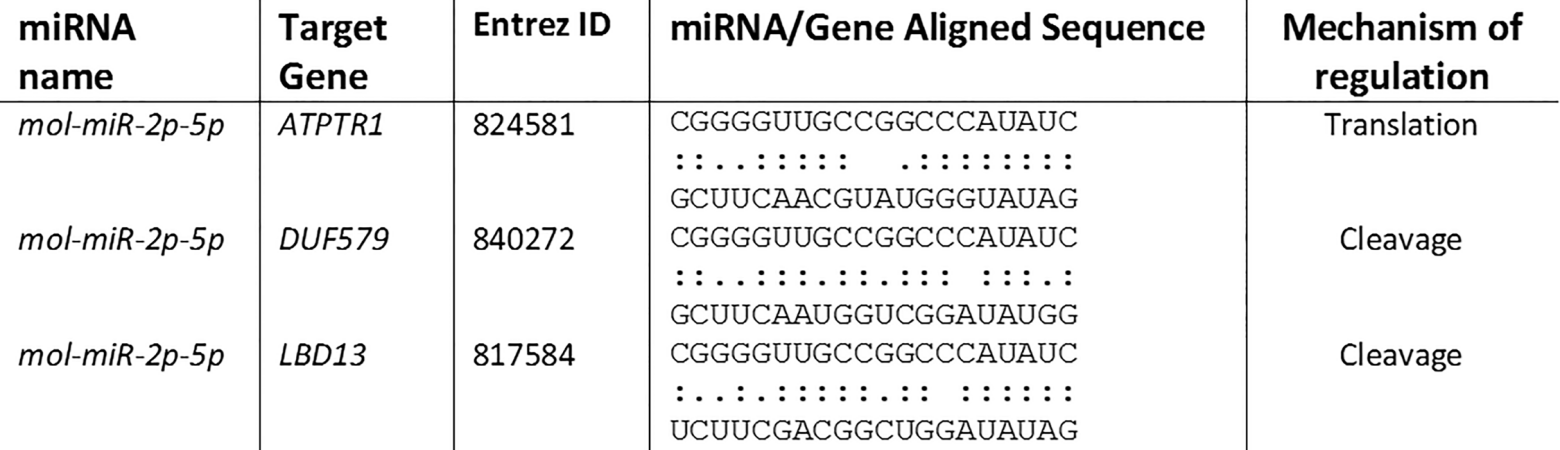
List of target genes for putative novel *M*. *oleifera* miRNAs.

Despite research of targets for all putative novel miRNA, only 3 target genes were selected (Fig 7) that involve *mol-miR2p-5p*, the most abundant uncharacterized miRNA highlighted by the miReader algorithm.

**Fig 8:**
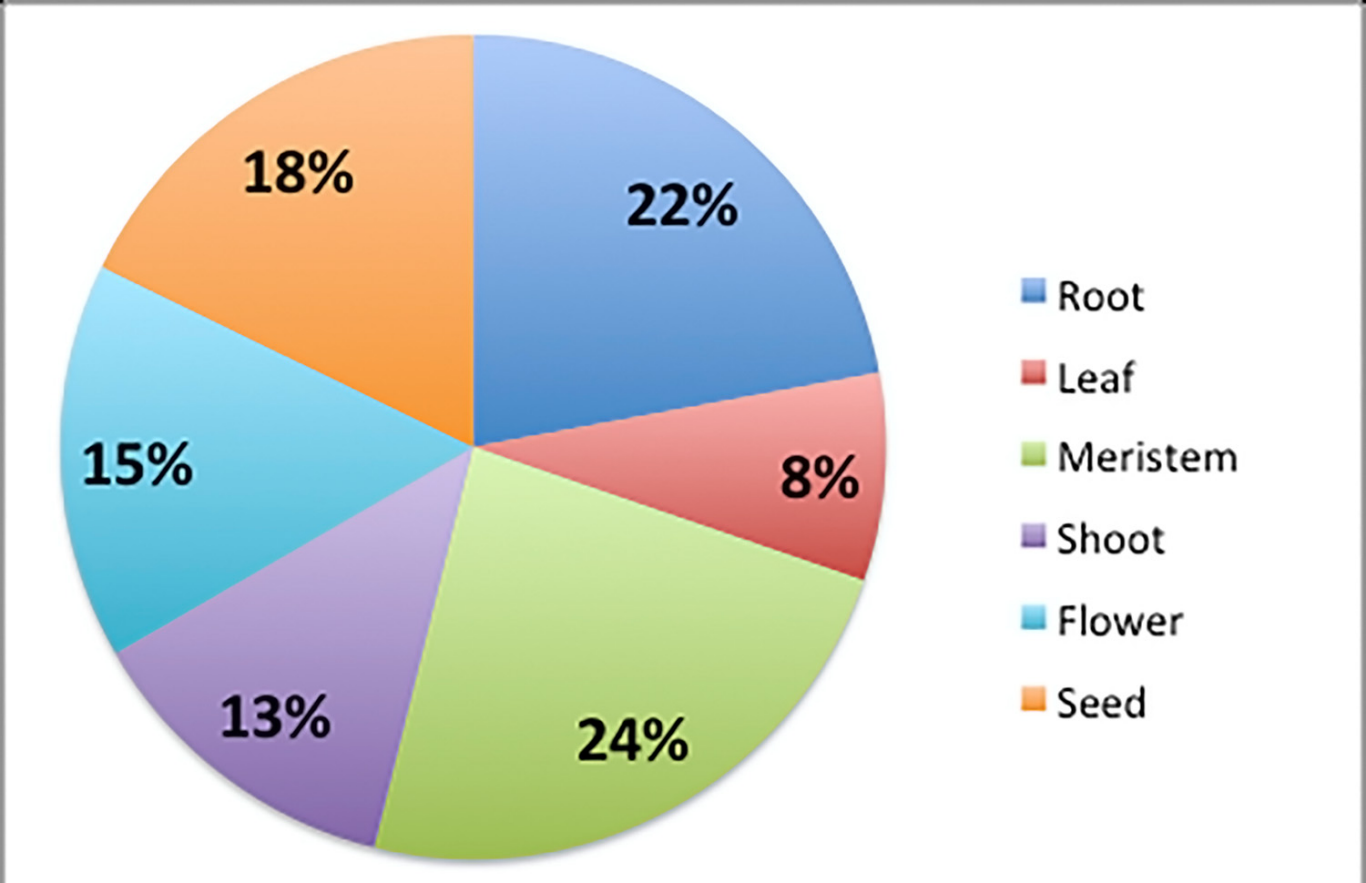
Known miRNA target functions. The miRNA target genes were classified according to the biological process.

Enrichment analysis with gene ontology terms on both target gene sets highlighted the relevance of these genes for plant organisms. As shown in Fig 8, most genes regulated by known miRNAs are involved in biological pathways localized to root, meristem and seed. Furthermore, these miRNAs participate in fundamental processes such as the development of anatomical (root, leaf) and reproductive structure, maintenance (flower, meristem, pollen and stamen), embryonic, and post-embryonic development. Genes putatively regulated by unknown miRNAs are involved in cellular metabolism of macromolecules, in particular macromolecules affecting protein transport and biosynthesis.

### Bioinformatics prediction of human gene targets for *M*. *oleifera* miRNAs

To investigate whether *M*. *oleifera* miRNAs might regulate human gene expression we used MirCompare software to search homologous human miRNAs. The most and conserved *mol-miR166i* resulted functional homologies with *hsa-miR6503-3p* (Table 5) that is involved in regulating inflammation [36], and *mol-miR393c* was homologous to *hsa-miR548ah-5p* that is involved in immune tolerance [37]. Further, *mol-mir168a* showed sequence homology with *hsa-miR579*, a human miRNA that normally regulates *TNFα* expression during endotoxin tolerance [38].

**Table 5:** Comparing *M*. *oleifera* miRNAs with their human counterparts using MirCompare software.

Human miRNA name	*M*. *oleifera* miRNA name	r-value	Sequence alignment details
*hsa-miR6503-3p*	*mol-miR166i*	0.67	-GGAC-AGG-U-CA--CC-CC
*hsa-miR548ah-5p*	*mol-miR393c*	0.65	-AAAG-GAU-GCA-UG-U--
*hsa-miR3940-5p*	*mol-miR159a*	0.65	-UGG-UUG--G-G-GCUCU-
*hsa-miR579*	*mol-miR168a*	0.57	UC--UUGGU--A---CG-GA-
*hsa-miR4534*	*mol-miR159a*	0.71	GGAU-GA-G-G-G-UCU
*hsa-miR1306-3p*	*mol-miR6478*	0.78	AC-UU-GCUC-G-UGGUG
*hsa-miR4703-3p*	*mol-miR6300*	0.72	GU-GUUGUA-U-UA-UG-
*hsa-miR5008-5p*	*mol-miR6300*	0.67	G-C-UUG--G-A-AGUGG
*hsa-miR4273*	*mol-miR398c*	0.67	GUGUUCUC-G-U-G-C--

COMIR software [33] predicted human genes that could be regulated by *M*. *oleifera* miRNAs. A subset of genes relevant to leukemia and acute myeloid (Table 6), which have key roles in the regulating cellular pathways such as apoptosis, cell cycle and protein degradation, have been found. Based on these results we identified *mol-miR168a* homologous to *hsa-miR579*, a human miRNA with many target genes including *SIRT1*. The complete report of predicted genes ranked by COMIR score (always upper than 0.9) is given in S2 Table.

**Table 6:** Predicting gene target in humans using COMIR software.

miRNA name	Ensemble Gene ID	Entrez ID	Target Gene	COMIR Score
*mol-miR166i*	ENSG00000082701	2932	*GSK3B*	0.9019
	ENSG00000064393	28996	*HIPK2*	0.9076
	ENSG00000156113	3778	*KCNMA1*	0.9038
	ENSG00000263162	8924 100653292	*HERC2*	0.9074
	ENSG00000169213	5865	*RAB3B*	0.9075
	ENSG00000171105	3643	*INSR*	0.9015
	ENSG00000078142	5289	*PIK3C3*	0.9044
	ENSG00000263162	8924 100653292	*HERC2*	0.9074
	ENSG00000119547	9480	*ONECUT2*	0.9075
*mol-miR393c*	ENSG00000178662	80034	*CSRNP3*	0.9075
	ENSG00000102908	10725	*NFAT5*	0.9074
	ENSG00000128585	4289	*MKLN1*	0.9074
	ENSG00000145907	10146	*G3BP1*	0.9074
	ENSG00000009413	5980	*REV3L*	0.9072
	ENSG00000010244	7756	*ZNF207*	0.9072
	ENSG00000100354	23112	*TNRC6B*	0.9072
	ENSG00000143190	5451	*pou2f1*	0.9072
	ENSG00000173611	286205	*Scai*	0.9072
	ENSG00000100731	22990	*pcnx*	0.9071
*mol-miR159a*	ENSG00000171435	283455	*KSR2*	0.9076
	ENSG00000119547	9480	*ONECUT2*	0.9076
	ENSG00000153721	154043	*CNKSR3*	0.9074
	ENSG00000261115	1.01E+08	*TMEM178B*	0.9074
	ENSG00000158445	3745	*KCNB1*	0.9072
	ENSG00000077157	4660	*PPP1R12B*	0.907
	ENSG00000196090	11122	*PTPRT*	0.907
	ENSG00000055609	58508	*KMT2C*	0.9068
	ENSG00000132549	157680	*VPS13B*	0.9065
	ENSG00000148019	84131	*CEP78*	0.9062
*mol-miR168a*	ENSG00000096717	23411	*SIRT1*	0.9087
	ENSG00000178562	940	*CD28*	0.9099
	ENSG00000134352	3572	*IL6ST*	0.9237
	ENSG00000118689	2309	*FOXO3*	0.9190
	ENSG00000106799	7046	*TGFBR1*	0.9115
	ENSG00000169967	10746	*MAP3K2*	0.9118
	ENSG00000175595	2072	*ERCC4*	0.9110
	ENSG00000149311	472	*ATM*	0.9187
	ENSG00000149948	8091	*HMGA2*	0.9119
	ENSG00000007372	5080	*PAX6*	0.9189
*mol-miR6478*	ENSG00000134313	57498	*KIDINS220*	0.9061
	ENSG00000106261	7586	*ZKSCAN1*	0.9058
	ENSG00000134909	9743	*ARHGAP32*	0.9055
	ENSG00000136709	55339 84826	*WDR33*	0.9055
	ENSG00000112706	3617	*IMPG1*	0.9051
	ENSG00000107331	20	*ABCA2*	0.9049
	ENSG00000088808	23368	*PPP1R13B*	0.9029
	ENSG00000167654	85300	*ATCAY*	0.9028
	ENSG00000189339	728661	*SLC35E2B*	0.9026
	ENSG00000180370	5062	*PAK2*	0.9025
*mol-miR6300*	ENSG00000178567	9852	*EPM2AIP1*	0.9076
	ENSG00000213699	54978	*SLC35F6*	0.9075
	ENSG00000197818	23315	*SLC9A8*	0.9072
	ENSG00000183751	10607	*TBL3*	0.9071
	ENSG00000206190	57194	*ATP10A*	0.9071
	ENSG00000166206	2562	*GABRB3*	0.9071
	ENSG00000198000	55035	*NOL8*	0.907
	ENSG00000172380	55970	*GNG12*	0.907
	ENSG00000152443	284309	*ZNF776*	0.9067
	ENSG00000133703	3845	*KRAS*	0.9066
*mol-miR398c*	ENSG00000055609	58508	*KMT2C*	0.9076
	ENSG00000164684	619279	*ZNF704*	0.9076
	ENSG00000145012	4026	*LPP*	0.9076
	ENSG00000151914	667	*DST*	0.9075
	ENSG00000158258	64084	*CLSTN2*	0.9074
	ENSG00000110436	6506	*SLC1A2*	0.9073
	ENSG00000064393	28996	*HIPK2*	0.9073
	ENSG00000118482	23469	*PHF3*	0.907
	ENSG00000143970	55252	*ASXL2*	0.907
	ENSG00000135968	9648	*GCC2*	0.907

### Synthetic *mol-miR168a* transfection and protein modulation

**Fig 9:**
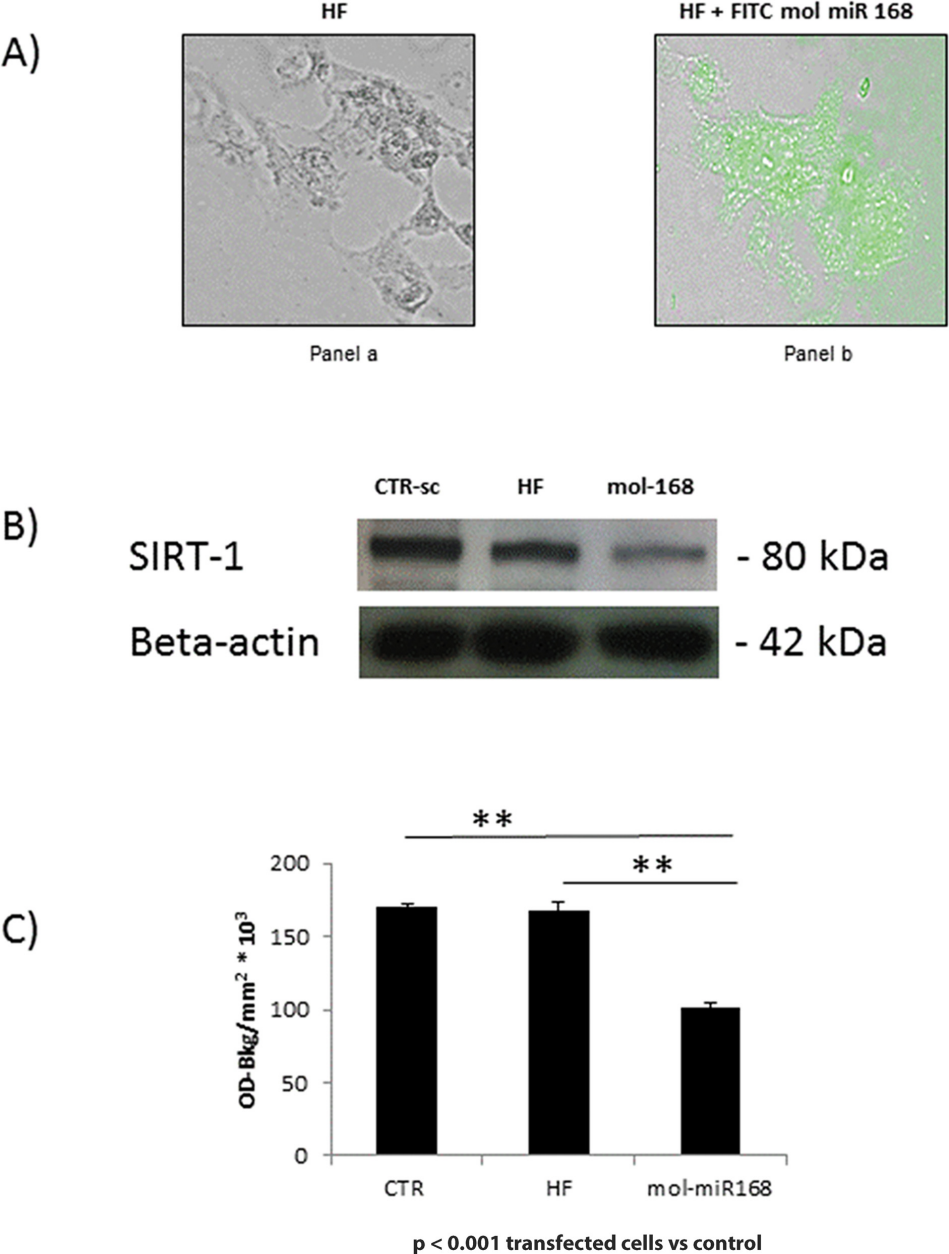
Experimental Validation of miRNA-mRNA human gene regulation. Histograms represent the mean values and ± the SD values. A) Presence of green cells in HepG2 cell lines (panel b) 72hrs after transfection with the synthetic FITC *mol-miR168a (EVOS FLoid cell imaging station*, *LifeTechnologies)*. Left panel: HepG2 cells transfected only with the lipofectamin (HF); right panel: HepG2 cells transfected with lipofectamin (HF) and synthetic FITC *mol-miR168a*. B) One representative experiment (out of three) of Western Blot analysis of *SIRT-1* expression in HepG2 cells after synthetic *mol-miR168a* transfection; *β-actin* expression, run on the same gel, indicates that an equal amount of protein was loaded for each sample. C) Western blot analysis for each sample was quantified by densitometry analysis and values were expressed as OD-Bkg/mm^2^*10^3^. Histograms represent mean values ± S.D. from three independent experiments performed on HepG2 cells. Statistical comparison of means using t-Test provided the following results: control 72h versus HF, not significant (NS); synthetic *mol-miR168a* versus control and versus HF, p<0.001.

In these preliminary studies, we tested whether an exogenous miRNA derived from *M*. *oleifera* might be functional in human cell line and we verified its ability to inhibit post transcriptional mRNA expression. In particular, transfecting Hepatoma cell line G2 with the synthetic FITC *mol-miR168a* was run. By 72 hours after transfection with FITC (Fig 9A, green cells, panel b), synthetic *mol-miR168a* increased the number of fluorescent cells by EVOS FLoid cell imaging station analysis (about 60–70% of HEPG2 cells resulted significantly and positively transfected). The transfection of synthetic *mol-miR168a*, a plant miRNA that show sequences homology with *hsa-miR579*, determined a significant decrease of *SIRT1* protein level in comparison with HF and control samples (Fig 9B and 9C).

## Discussion

Medicinal plants are studied to identify new therapeutic agents and understand their mechanisms of action against a variety of human diseases. Medicinal plant compounds are known to have high biocompatibility, low toxicity, and potential biological activity [39]. In particular, *M*. *oleifera* is commonly known and used for its health benefits [2]. For centuries and in many cultures around the world, *M*. *oleifera* has been used to treat human diseases [1] and is an example of traditional medicine that is increasingly popular among African countries, due in part to their poor economic conditions. A recent collaboration between universities in Italy and Cameroon has the objective of investigating the anti-oxidant and anti-tumor properties of *M*. *oleifera* [4]. The incredible *Moringa’s* usage as medicinal, claimed by real-life experience, is now slowly being confirmed by scientific experiences.

Here we address a new aspect of plant biology that may have substantial impact on understanding of medicinal activity. The miRNA expressed in plants may comprise an independent category of medicinal agents capable of providing beneficial effects for human consumers of valuable plants.

Recently, Shahzad and collaborators [3] reported a study of DNA markers for genetic diversity and population structure in worldwide collections of *M*. *oleifera*. However, no study on the breadth of miRNA has yet been reported for *M*. *oleifera*, therefore, the knowledge about molecular mechanisms for compounds produced by *M*. *oleifera* is still limited. Here, we describe the identification and characterization of conserved and possibly novel miRNAs from seed of *M*. *oleifera* plants grown in Cameroon. We have used bioinformatics tools to understand their potential roles in diverse biological processes and their possible role in human gene regulation.

Since the discovery of sRNAs (miRNAs and siRNAs) as regulators of gene expression in *C*. *elegans* [40] and, more recently, the discovery of plant miRNAs [41–42], the study on miRNAs has become an important and integral topic in functional genomic research.

In plants, miRNAs regulate gene expression at the post-transcriptional level by degrading or repressing translation of target mRNAs [43]. A high number of experimental and computational studies have indicated that mature miRNAs are evolutionarily conserved in plants. miRNA-mediated gene regulation has an ancient phylogenetic origin and plays an important regulatory role in physiological processes [43], many aspects of plant growth, development and environmental adaptability.

High-throughput sequencing based on Illumina technology has become a good approach for identification and expression analysis of miRNAs in several plant species, like *Arabidopsis* [44] and other plant species [45–46], although the presence of biases introduced during the construction of sRNA libraries, primarily derived from the adaptor ligation steps [47], requires a carefully attention.

In this study, we identified 94 conserved miRNAs belonging to 40 families, two uncharacterized miRNA duplexes with 48 and 3 targets gene for conserved and uncharacterized miRNAs respectively. As in earlier studies [45–46], the majority of highly conserved miRNAs from *Moringa*-seed that were predicted by our analysis resulted to be evolutionarily conserved across plant species and to have high levels of expression. For example, *miR166*, *miR393*, *miR167*, *miR396*, *miR159* and *miR156* families are well conserved among other plant species [48] and have a fundamental role in plant biology. Other miRNA families, such as *miR408* or *miR1515* were present at lower abundance. *mol-miR166* families had the highest number of reads (80,612); in particular, *mol-miR166i* and *mol-miR166* were the most and the least abundant miRNAs in these families, respectively. Results indicate that different members of the same miRNA family have differing levels of expression.

The most and least conserved miRNA families may have evolved to play different roles in plant biology. For example, *miR156*, *miR159*, *miR166* and *miR160* target *SPL*, *MYB*, *PHV* and *ARF genes* respectively. These transcription factors are important to plant growth and development. The *miR159* was reported to target *MYB101* and *MYB33* transcription factors, which are positive regulators of *ABA* signaling during *Arabidopsis* seed germination. Indeed, *miR159* may play a role in seed germination [49]. *MiR156* plays crucial role in the control of juvenile-to-adult transition in plants by targeting the *SQUAMOSA PROMOTER BINDING PROTEIN LIKE* (*SPL*) plant-specific transcription factors. *SPLs* affect processes like leaf development, shoot maturation, phase change and flowering in plants [50–52]. A recent study showed that *miR156* regulates shoot regenerative capacity and a gradual increase in *miR156* leads to a decline in shoot regenerative capacity for old plants [53]. *Auxin response factors* were found as a predicted target of *miR160*, while research studies assessed that this miRNA targets *ARF10*, *ARF16* and *ARF17* and regulates various aspects of plant development in *Arabidopsis* [54]. *MiR166/165* is an example of well-studied plant miRNAs implicated in various aspects of plant development. The *miR166/165* negatively regulates its targets *Class III Homeodomain Leucine-Zipper* (*HD-ZIPIII*) *transcription factors* that in turn regulate the polarity establishment in leaves and vasculature and radial patterning of root. The majority of *HD-ZIPIII* gene family members consisting of *PHABULOSA* (*PHB*), *PHAVOLUTA* (*PHV*), *REVOLUTA* (*REV*), *ATHB8*, and *ATHB15*, are conserved in several land plants including bryophytes, lycopods and seed plants [55–58]. *MiR393* is a conserved miRNA family discovered in many plants [41]. In *A*. *thaliana*, four F-box genes *TIR1 (TRANSPORT INHIBITOR RESPONSE PROTEIN1)*, *AFB1*, *AFB2* and *AFB3* (*AUXIN SIGNALING F-BOX*) were validated as *miR393* targets [59]. In rice, overexpression of *miR393* negatively regulates mRNAs of *TIR1* and *AFB2* [60]. *TIR1* and *AFB2* interact with *IAA* (*INDOLE-3-ACETIC ACID*) proteins, probably releasing the activities of *ARFs* (*AUXIN RESPONSE FACTORS*) and increasing resistance to auxin. The change in auxin response consequently affects diverse aspects of plant growth and development, such as flag leaf inclination, primary root growth, crown root initiation and seed development.

In this study, three possible novel miRNA targets were found: *mol-miR2p-5p* could affect target genes involved in macromolecule metabolism, in particular cellular protein transport and biosynthesis. In *Arabidopsis thaliana*, the *ATPTR1* gene seems to be involved in long-distance transport of di- and tri-peptides during seed germination [61] while *DUF579* affects xylan biosynthesis and modulates cell wall biosynthesis [62]. Further research on possible targets in other plants may provide important evidence to facilitate the understanding of these novel miRNA functions. Additional studies into novel *M*. *oleifera* miRNAs may shed light on their roles in *M*. *oleifera* biological processes.

The final phase of our bioinformatics analysis focused on the potential for human gene regulation by the most conserved *M*. *oleifera* miRNAs. The possibility of identifying plant miRNAs able to regulate human genome expression may be highly important in future studies on the nutritional value and medical usage of food. The combined use of MirCompare and COMIR software on the massive collection of data has identified a small number of human genes that might be regulated by *M*. *oleifera* miRNAs. These gene targets include cell-cycle regulation and signaling through the *p53* pathway; genes related to some classes of cancers including leukemia, acute myeloid and lipoma. For instance, *Sirtuins* have important roles in cell cycle, apoptosis, metabolic regulation and inflammation. The human genome encodes seven *Sirtuin* isoforms *SIRT1*-*SIRT7* with varying intracellular distribution; a number of studies reported evidence for their roles in a spectrum of disease like cancer, diabetes, obesity and neurodegenerative diseases [63]. Recent evidence suggests that genomic stability requires cooperation of *p53* and *SIRT1* [64]. Our transfection experiments showed that *mol-miR168a* identified by MirCompare-COMIR software inhibited translation of *SIRT1 mRNA* in cancer cells. We focused our attention on *mol-miR168a* because this is the first plant miRNA involved in cross-kingdom activity (*osa-miR168a* shown high sequence homology with *mol-miR168a*) [14] and it has functional homology with *hsa-miR579*, a putative regulator of the *SIRT1* gene.

In conclusion, we have identified a population of *Moringa*-specific miRNAs that could help our understanding of the regulatory role of miRNAs in this plant. Our results demonstrate that the differentially expressed miRNAs and predictions for their target genes provides a basis for further understanding *M*. *oleifera* seed miRNAs and biological processes in which they are involved. Further studies are necessary to search for more miRNAs that are novel and to validate their targets by expression analysis during seed development stage.

## Supporting Information

S1 Fig Sequence analysis workflow. Schematization of the process for the identification of known and novel miRNAs.
(TIF)

S2 Fig Workflow of miRNA target prediction. Plant targets were predicted using PsRNA Target. Enrichment analysis for all the predicted targets were conducted using Plant GSEA. MirCompare was used to predict cross-kingdom interaction targets in human.
(TIF)

S1 File Quality control analysis before the removal of the sequencing adapter. Quality scores across all bases (**Figure A in S1 File**). Quality score distribution over all sequences (**Figure B in S1 FIle**). Percentage of adapter sequence (**Figure C in S1 File**).
(DOCX)

S2 File Quality control analysis after the removal of the sequencing adapter. Quality scores across all bases (**Figure A in S1 File**). Quality score distribution over all sequences (**Figure B in S1 File**). Percentage of adapter sequence (**Figure C in S1 File**).
(DOCX)

S1 Table Known microRNAs in *M*. *oleifera* juvenile seed, with additional detailed information. (DOCX)

S2 Table Complete prediction report of gene target in humans, using COMIR software. (DOCX)
