# Supplementary figures and images for: MicroRNA from Moringa oleifera: Identification by High Throughput Sequencing and Their Potential Contribution to Plant Medicinal Value
Source: PLoS One. 2016 Mar 1;11(3):e0149495. doi: 10.1371/journal.pone.0149495 (PMC4773123; doi:10.1371/journal.pone.0149495)

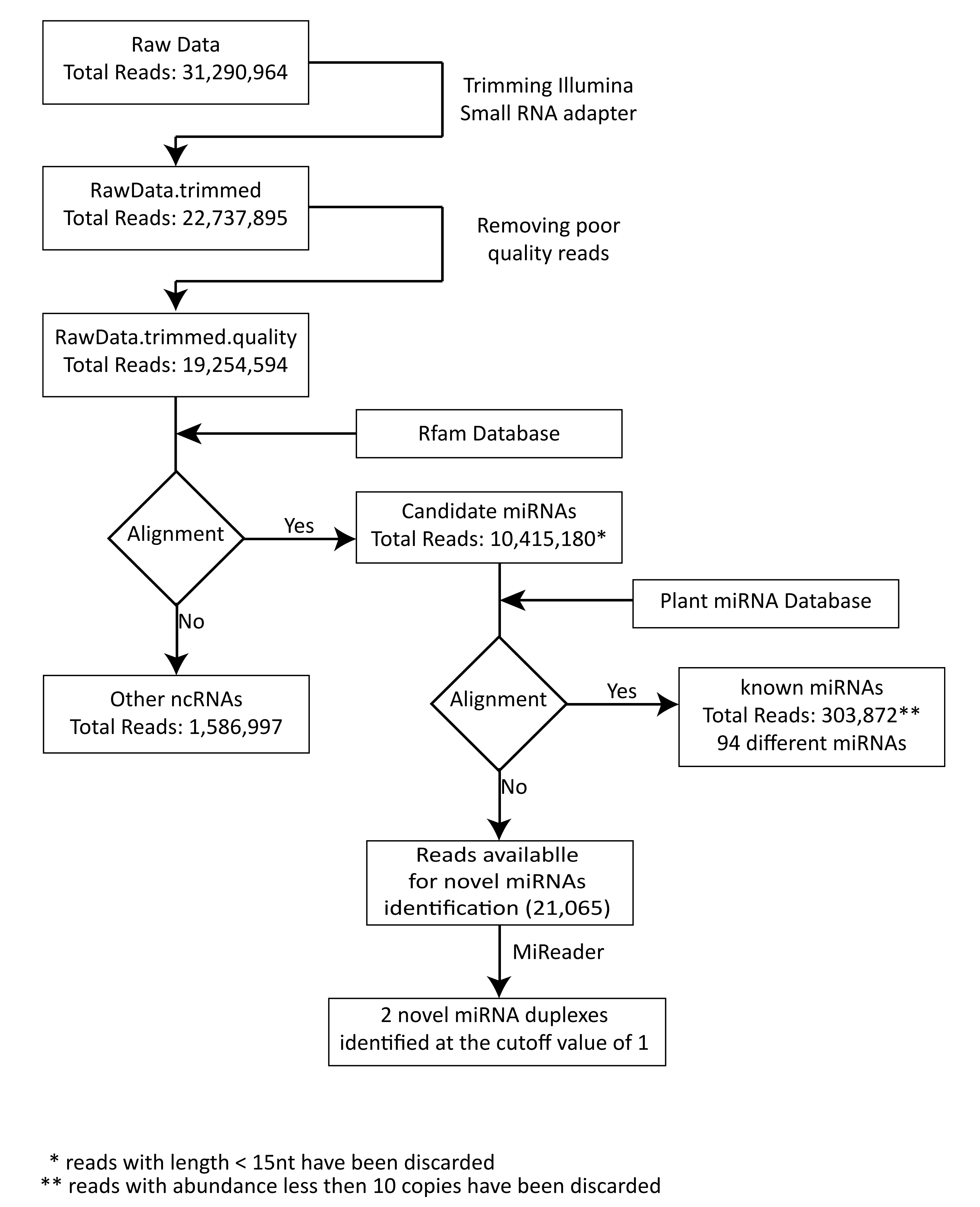

Supplement: S1 Fig — Schematization of the process for the identification of known and novel miRNAs. (TIF) [file pone.0149495.s001.tif]

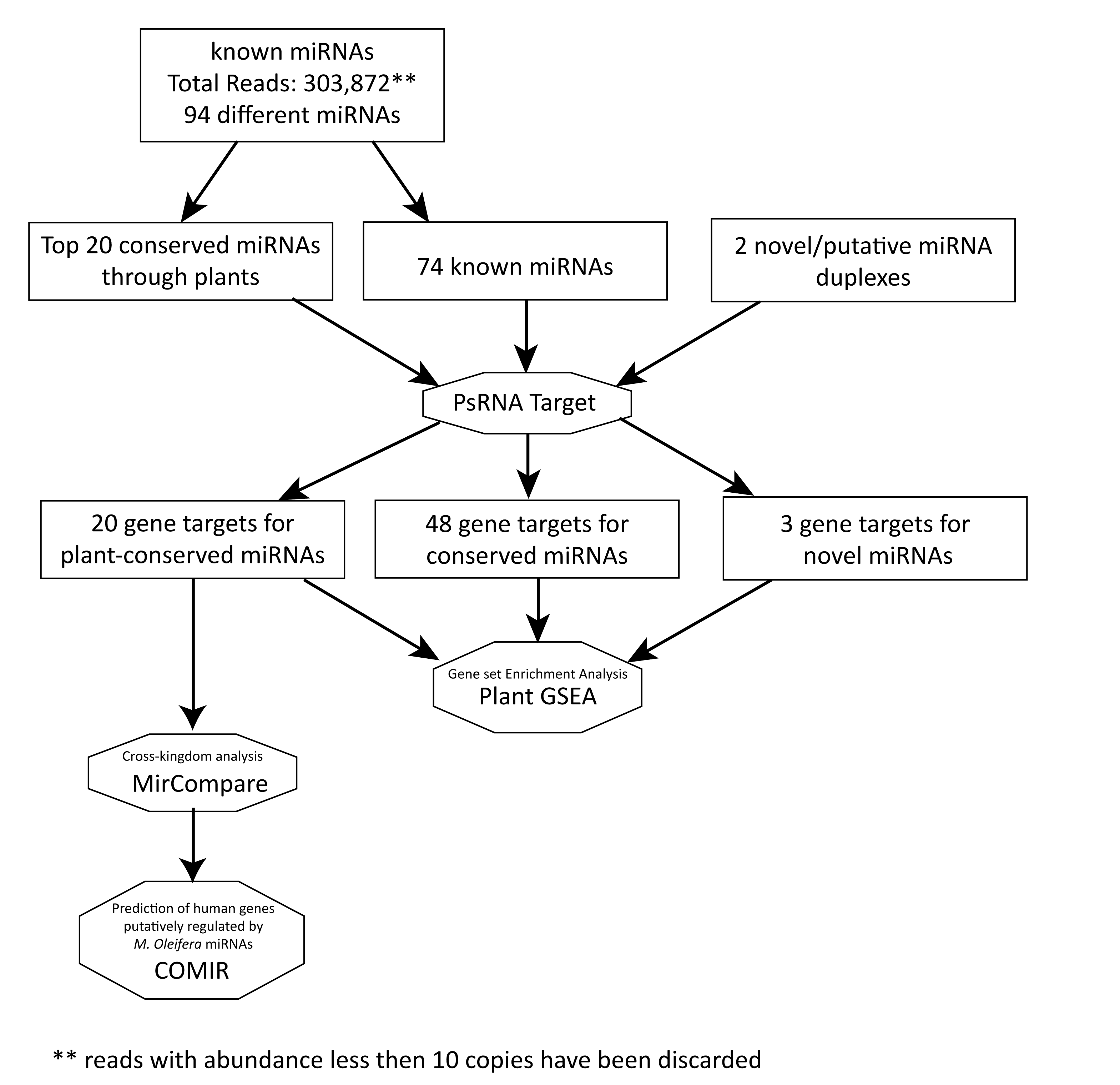

Supplement: S2 Fig — Plant targets were predicted using PsRNA Target. Enrichment analysis for all the predicted targets were conducted using Plant GSEA. MirCompare was used to predict cross-kingdom interaction targets in human. (TIF) [file pone.0149495.s002.tif]
