# Supplementary material for: MicroRNA from Moringa oleifera: Identification by High Throughput Sequencing and Their Potential Contribution to Plant Medicinal Value
Source: PLoS One. 2016 Mar 1;11(3):e0149495. doi: 10.1371/journal.pone.0149495 (PMC4773123; doi:10.1371/journal.pone.0149495)

**S2 File**. **Quality control analysis** **after the removal of the sequencing adapter**. Quality scores across all bases (**Figure A**). Quality score distribution over all sequences (**Figure B**). Percentage of adapter sequence (**Figure C**).


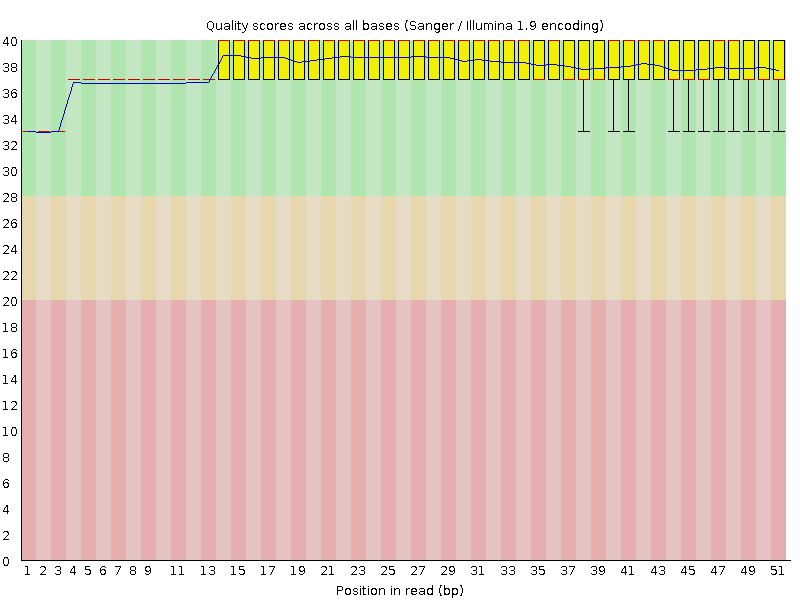


A

B

C


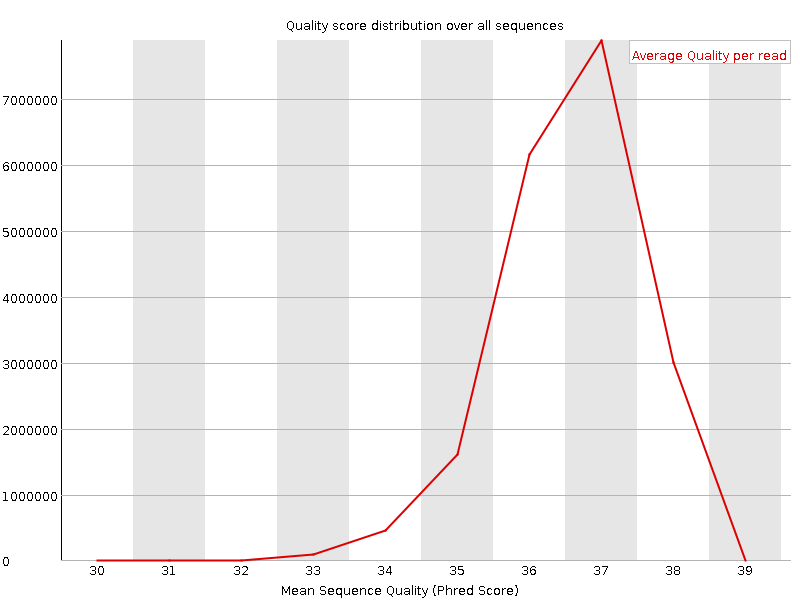

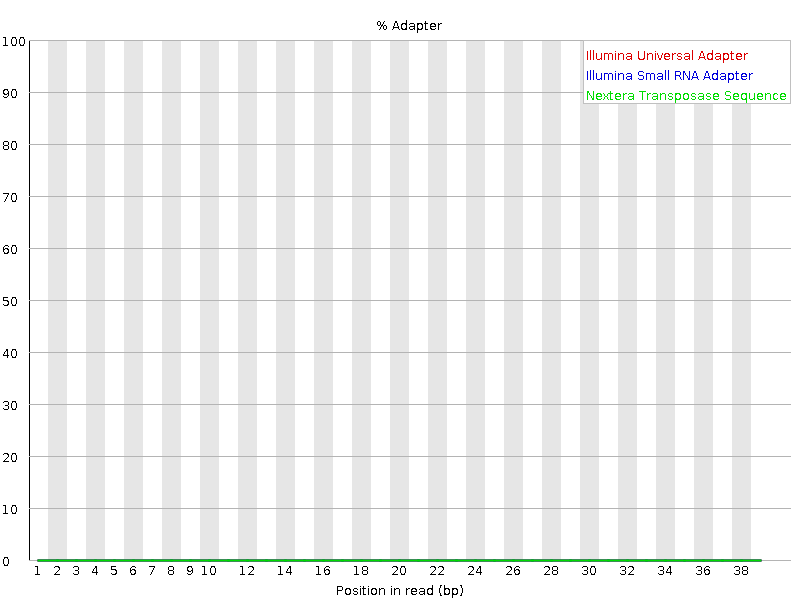

Supplement: S2 File — Quality scores across all bases (Figure A in S1 File). Quality score distribution over all sequences (Figure B in S1 File). Percentage of adapter sequence (Figure C in S1 File). (DOCX) [file pone.0149495.s004.docx]
