# Supplementary material for: MicroRNA from Moringa oleifera: Identification by High Throughput Sequencing and Their Potential Contribution to Plant Medicinal Value
Source: PLoS One. 2016 Mar 1;11(3):e0149495. doi: 10.1371/journal.pone.0149495 (PMC4773123; doi:10.1371/journal.pone.0149495)
Supplement: S1 Table — (DOCX) [file pone.0149495.s005.docx]

**S1 Table**. Known microRNAs in *Moringa oleifera* juvenile seed, with additional detailed information.

| **miRNA family** | **miRNA members** | **miRNA sequence** | **miRNA* sequence** | **Secondary structure** | **Read Counts** | |
| --- | --- | --- | --- | --- | --- | --- |
|  |  |  |  |  | **miR** | **miR*** |
| mol-miR156 | *mol-miR156* | CUGACAGAAGAGAGUGAGCAC |  |  | 2459 |  |
|  | *mol-miR156d* |  | GCUCUCUAUGCUUCUGUCAUCA |  |  | 23 |
|  | *mol-miR156f* | CUGACAGAAGAGAGUGAGCA | CUCACUUCUCUUUCUGUCAAUC | 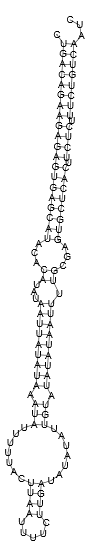 | 86 | 34 |
|  | *mol-miR156g* | CGACAGAAGAGAGUGAGCAC |  |  | 77 |  |
|  | *mol-miR156h* | UGACAGAAGAAAGAGAGCAC | GCUCUCUUUCCUUCUGCCACC | 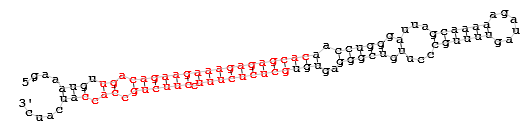 | 33 | NA |
|  | *mol-miR156j* | GUUGACAGAAGAGAGUGAGCAC |  |  | 2546 |  |
|  | *mol-miR156q* | UGACAGAAGAGAGUGAGCACU |  |  | 2476 |  |
|  | *mol-miR156t* | UUGACAGAAGAGAGAGAGCAC |  |  | 111 |  |
| mol-miR157 | *mol-miR157a* | UUGACAGAAGAUAGAGAGCAC | GCUCUCUAGCCUUCUGUCAUCA | 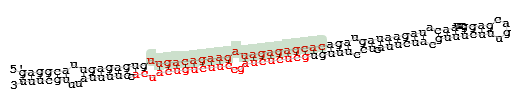 | 316 | NA |
|  | *mol-miR157d* | UGACAGAAGAUAGAGAGCAC | GCUCUCUAUGCUUCUGUCAUC | 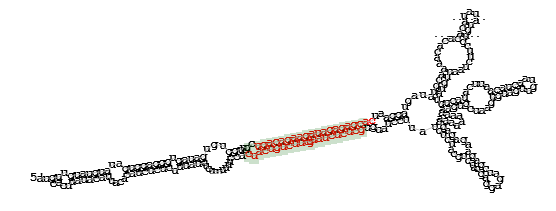 | NA | 23 |
| mol-miR159 | *mol-miR159* | AGCUCCCUUCGAUCCAAUC | CUUGGAUUGAAGGGAGCUCU | 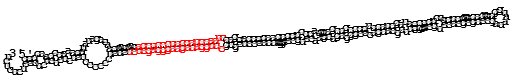 | NA | 48 |
|  | *mol-miR159a.1* | UUUGGAUUGAAGGGAGCUCUA |  | 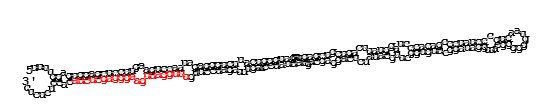 | 11908 |  |
|  | *mol-miR159b* |  | UUUGGAUUGAAGGGAGCUCUU |  |  | 2555 |
|  | *mol-miR159b.1* |  | UUUGGAUUGAAGGGAGCUCUG | 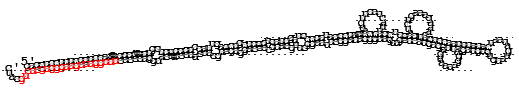 |  | 2492 |
|  | *mol-miR159c* |  | UUUGGAUUGAAGGGAGCUCCU | 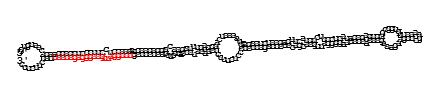 |  | 1917 |
|  | *mol-miR159d* | AUUGGAUUGAAGGGAGCUCCG |  | 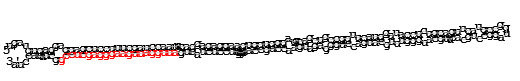 | 21 |  |
|  | *mol-miR159f* | CUUGGAUUGAAGGGAGCUCUA |  | 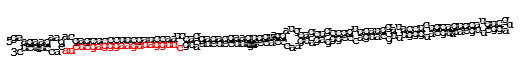 | 442 |  |
| mol-miR160 | *mol-miR160h* | UGCCUGGCUCCCUGUAUGCCAUU |  | 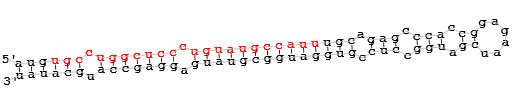 | 25 |  |
| mol-miR162 | *mol-miR162* |  | UCGAUAAACCUCUGCAUCCAG | 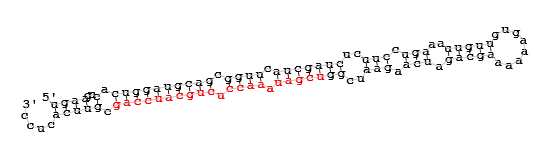 |  | 158 |
|  | *mol-miR162a* | UCGAUAAACCUCUGCAUCCA |  | 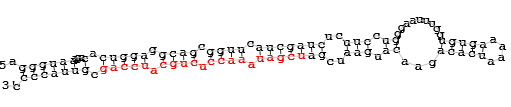 | 16 |  |
| mol-miR164 | *mol-miR164a* | UGGAGAAGCAGGGCACGUGAA |  | 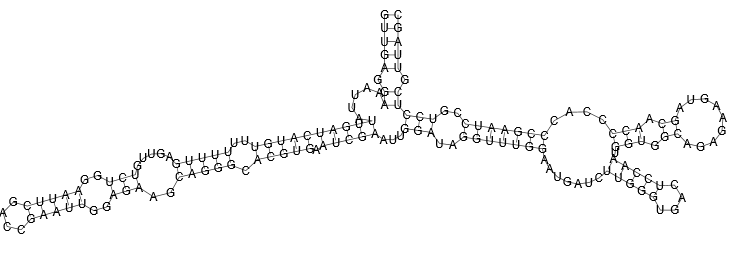 | 13 |  |
|  | *mol-miR164c* | UGGAGAAGCAGGGCACGUGCG |  | 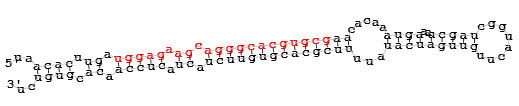 | 13 |  |
|  | *mol-miR164d* | UGGAGAAGCAGGGCACGUGCA |  | 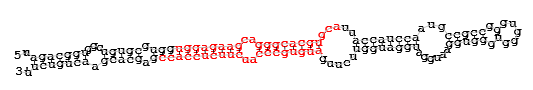 | 373 |  |
| mol-miR165 | *mol-miR165a* | UCGGACCAGGCUUCAUCCCCC |  | 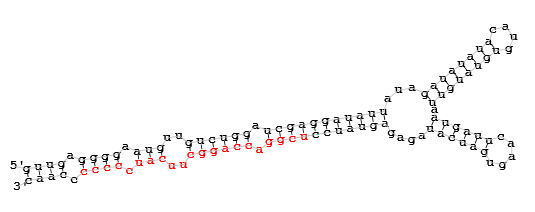 | 368 |  |
| mol-miR166 | *mol-miR166* | CCGGACCAGGCUUCAUCCCAG |  | 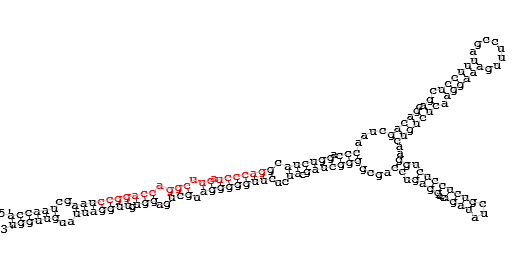 | 14 |  |
|  | *mol-miR166b* | UCGGACCAGGCUUCAUUCCCUU |  | 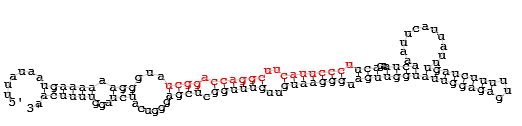 | 2367 |  |
|  | *mol-miR166e* | GGACCAGGCUUCAUUCCCC |  | 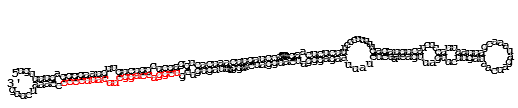 | 5541 |  |
|  | *mol-miR166h* | UCGGACCAGGCUUCAUUCCCGU |  | 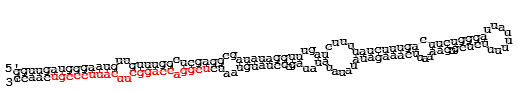 | 2923 |  |
|  | *mol-miR166i* | UCGGACCAGGCUUCAUUCCCCC |  | 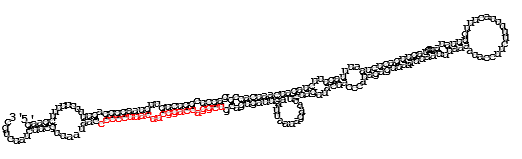 | 65243 |  |
|  | *mol-miR166j* | UCCGGACCAGGCUUCAUUCCC |  | 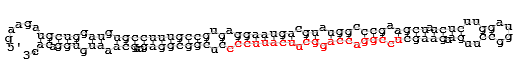 | 546 | NA |
|  | *mol-miR166k* | GGAUUGUUGUCUGGCUCGGUG | UCGGACCAGGCUUCAAUCCCU | 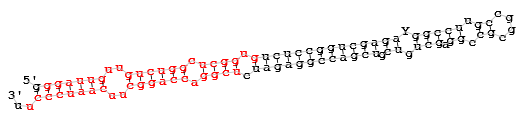 | NA | 240 |
|  | *mol-miR166u* | UCUCGGACCAGGCUUCAUUC |  | 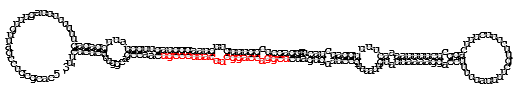 | 3738 |  |
| mol-miR167 | *mol-miR167* | UCAAGCUGCCAGCAUGAUCUA | AGAUCAUGUGGCAGUUUCACC | 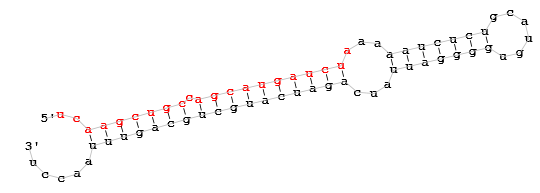 | 27 | 514 |
|  | *mol-miR167a* | UGAAGCUGCCAGCAUGAUCUC |  | 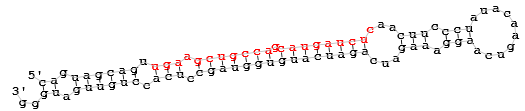 | 3881 |  |
|  | *mol-miR167b* | UGAAGCUGCCAGCAUGAUCUA |  | 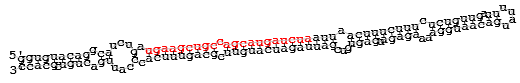 | 3834 |  |
|  | *mol-miR167c* | UGAAGCUGCCAGCAUGAUCUGG |  | 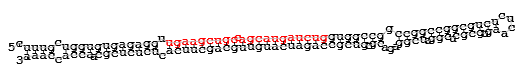 | 3908 |  |
|  | *mol-miR167c* | UAAGCUGCCAGCAUGAUCUUG | UAGGUCAUGCUGGUAGUUUCACC | 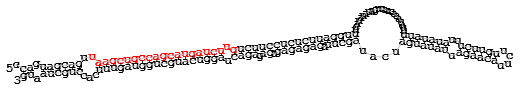 | 271 | NA |
|  | *mol-miR167d* | UGAAGCUGCCAGCAUGAUCUGA |  | 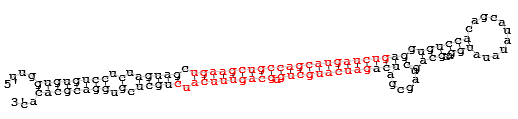 | 4007 |  |
|  | *mol-miR167h* | UGAAGCUGCCAGCAUGAUCUUA | AGAUCAUGUGGCAGUUUCACC | 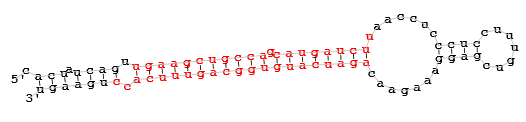 | 18563 | NA |
|  | *mol-miR167i* | UCAUGCUGGCAGCUUCAACUGGU |  | 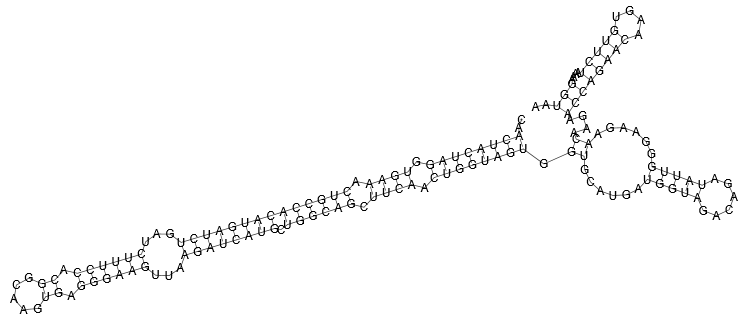 | 586 |  |
| mol-miR168 | *mol-miR168* | AUUCAGUUGAUGCAAGGCGGGAUC |  | 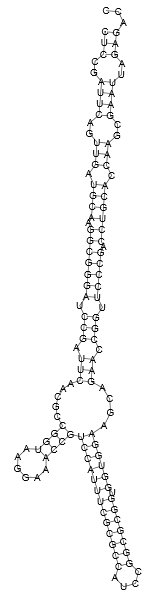 | 91 |  |
|  | *mol-miR168a* | UCGCUUGGUGCAGGUCGGGAC |  | 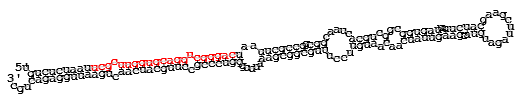 | 165 |  |
|  | *mol-miR168c* | UCGCUUGGUGCAGGUCGGGAC | CCCGCCUUGCAUCAACUGAAU | 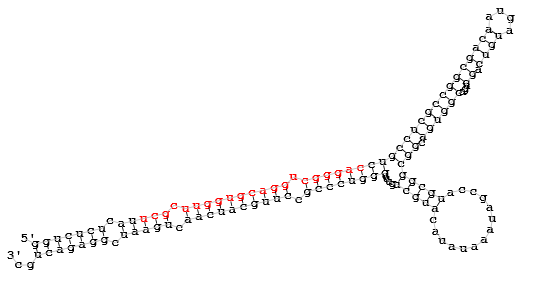 | NA | 91 |
|  | *mol-miR168d* | UCGCUUGGUGCAGGUCGGGAA | CCCGCCUUGCAUCAACUGAAU | 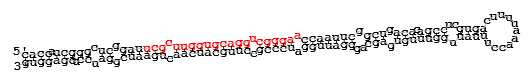 | 517 | NA |
| mol-miR169 | *mol-miR169d* | UAGCCAAGGAUGACUUGCCU |  | 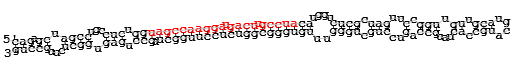 | 22 |  |
| mol-miR170 | *mol-miR170* | UAUUGGCCUGGUUCACUCAGA | UGAUUGAGCCGUGUCAAUAUC | 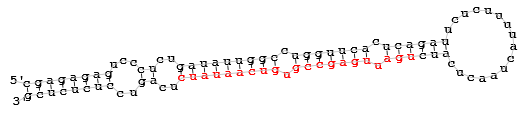 | NA | 77 |
| mol-miR171 | *mol-miR171a* | UGAUUGAGCCGUGCCAAUAU |  | 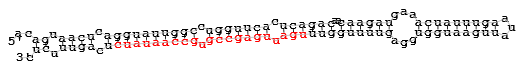 | 423 |  |
|  | *mol-miR171c* | UAUUGACGCGGUUCAAUUCGA | UGAUUGAGCCGUGCCAAUAUC | 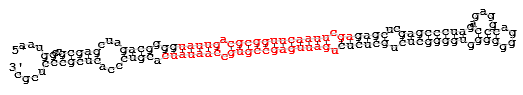 | NA | 503 |
|  | *mol-miR171d* |  | UUGAGCCGUGCCAAUAUCACG | 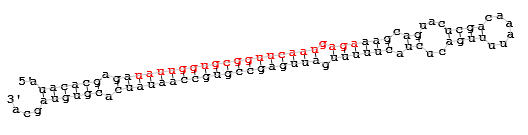 |  | 410 |
| mol-miR172 | *mol-miR172m* | GGAGCAUCAUCAAGAUUCACA | AGAAUCUUGAUGAUGCUGCAG | 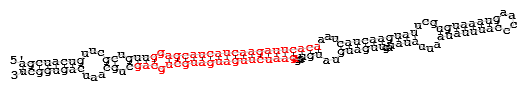 |  | 68 |
| mol-miR319 | *mol-miR319* | UUGGACUGAAGGGAGCUCCC |  | 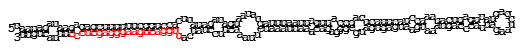 | 1204 |  |
|  | *mol-miR319e* | UUUGGACUGAAGGGAGCUCCU |  | 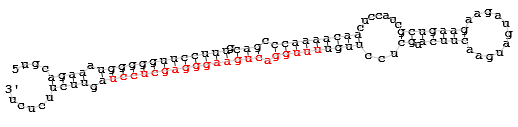 | 2843 |  |
|  | *mol-miR319g* | UUGGACUGAAGGGAGCUCCUUC |  | 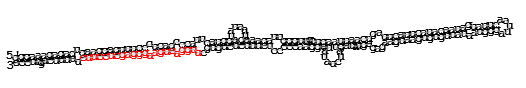 | 1240 |  |
| mol-miR390 | *mol-miR390a* | AAGCUCAGGAGGGAUAGCGCC | CGCUAUCCAUCCUGAGUUUCA | 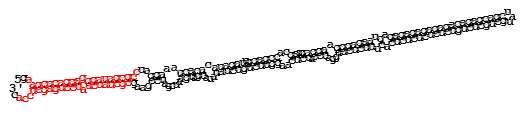 | 1128 | 82 |
|  | *mol-miR390d* | AAGCUCAGGAGGGAUAGCGCC | CGCUAUCCAUCCUGAGUUUUA | 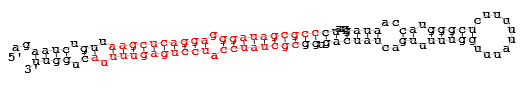 | NA | 15 |
|  | *mol-miR390e* | AGCUCAGGAGGGAUAGCGCC | CGCUAUCUAUCCUGAGCUCCA | 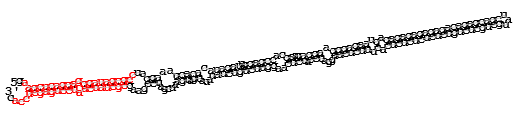 | 203 | NA |
| mol-miR393 | *mol-miR393a* | CAUCCAAAGGGAUCGCAUUGA |  | 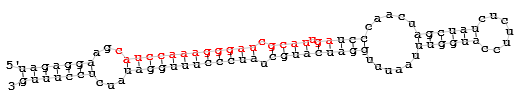 | 591 |  |
|  | *mol-miR393b* | UCCAAAGGGAUCGCAUUGAUC |  | 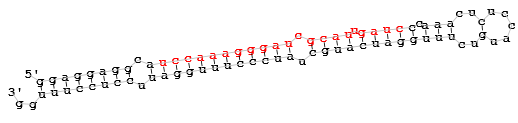 | 1730 |  |
|  | *mol-miR393c* | UCCAAAGGGAUCGCAUUGAUCU | AUCAGUGCAAUCCCUUUGGAAU | 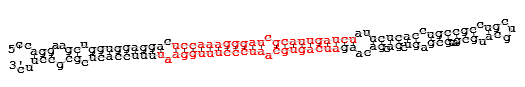 | 11813 | NA |
|  | *mol-miR393h* | UUCCAAAGGGAUCGCAUUGAUC |  | 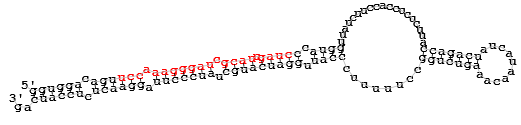 | 9974 |  |
| mol-miR394 | *mol-miR394b* | UUGGCAUUCUGUCCACCUCC | CUGUUGGUCUCUCUUUGUAA | 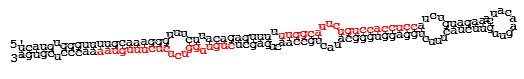 | 1147 | NA |
| mol-miR395 | *mol-miR395a* | CUGAAGUGUUUGGGGGAACUC |  | 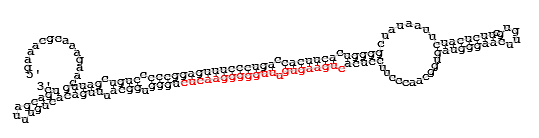 | 84 |  |
|  | *mol-miR395d* | UGAAGUGUUUGGGGGAACUUU |  | 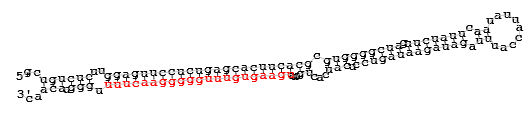 | 26 |  |
|  | *mol-miR395g* | UUGAAGUGUUUGGGGGAACUC |  | 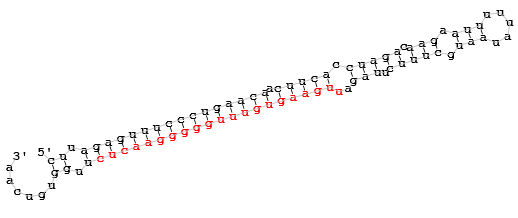 | 43 |  |
|  | *mol-miR395h* | AUGAAGUGUUUGGGGGAACUU |  | 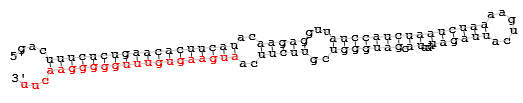 | 26 |  |
| mol-miR396 | *mol-miR396a* | UUCCACAGCUUUCUUGAACGU |  | 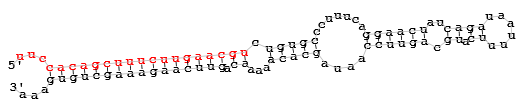 | 265 |  |
|  | *mol-miR396c* | UUCCACAGCUUUCUUGAACUU |  | 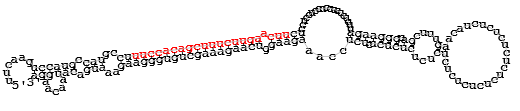 | 7917 |  |
|  | *mol-miR396e* | UUCCACAGGCUUUCUUGAACUG |  | 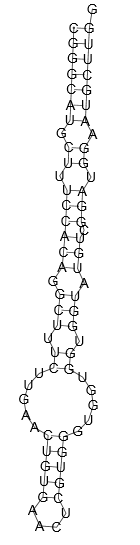 | 188 |  |
|  | *mol-miR396g* | UCCCACAGCUUUAUUGAACUG | GUUCAAGAAAGCUGUGGAAGA | 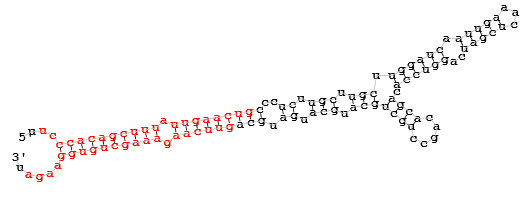 | 12 | 265 |
|  | *mol-miR396h* | UCCACAGCUUUCUUGAACUG |  | 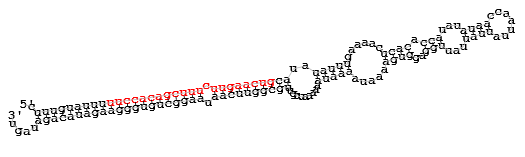 | 419 |  |
| mol-miR397 | *mol-miR397a* | UCAUUGAGUGCAGCGUUGAUG |  | 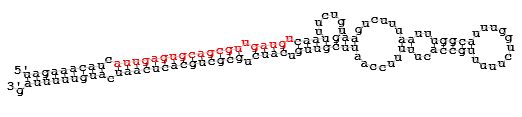 | 529 |  |
| mol-miR398 | *mol-miR398a* | GGGUUGAUUUGAGAACAUAUG | UAUGUUCUCAGGUCGCCCCUG | 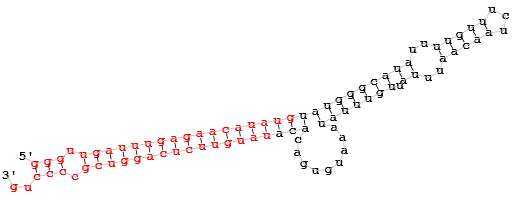 | NA | 23 |
|  | *mol-miR398c* | UGUGUUCUCAGGUCGCCCCUG |  | 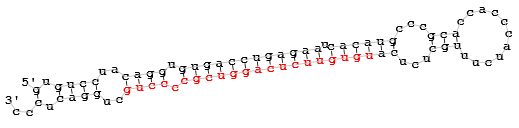 | 3285 |  |
|  | *mol-miR398f* | GGUGUUCUCAGGUCGCCCCUG |  | 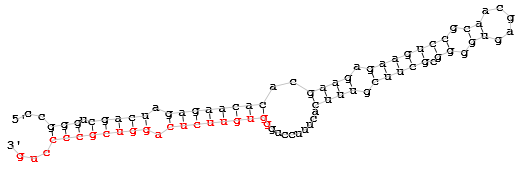 | 115 |  |
| mol-miR399 | *mol-miR399a* | CGCCAAAGGAGAGUUGCCCUU |  | 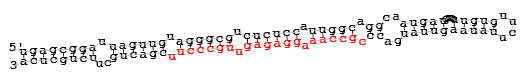 | 119 |  |
|  | *mol-miR399d* | UGCCAAAGGAGAGUUGCCCUU |  | 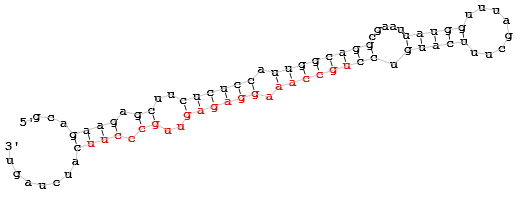 | 76 |  |
| mol-miR403 | *mol-miR403* | UUAGAUUCACGCACAAACUCG |  | 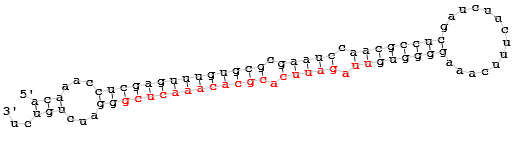 | 5277 |  |
|  | *mol-miR403a* | UUAGAUUCACGCACAAACUUG |  | 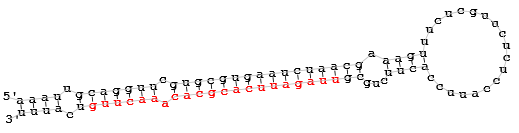 | 324 |  |
| mol-miR408 | *mol-miR408* | CAGGGAUGAGGCAGAGCAUGG | CUGCACUGCCUCUUCCCUGGC | 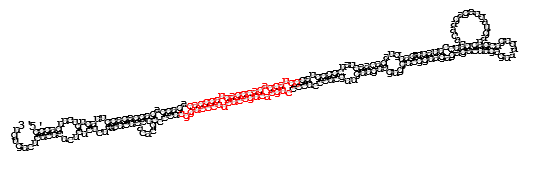 | NA | 27 |
| mol-miR530 | *mol-miR530* | UCUGCAUUUGCACCUGCACCU |  | 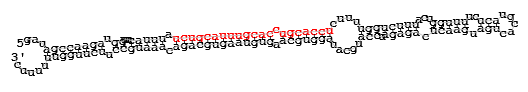 | 950 |  |
| mol-miR535 | *mol-miR535a* | UGACAACGAGAGAGAGCACGC |  | 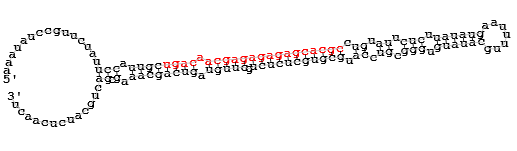 | 347 |  |
| mol-miR827 | *mol-miR827* | UUUGUUGAUUGACAUCUAUAC | UUAGAUGACCAUCAACAAACG | 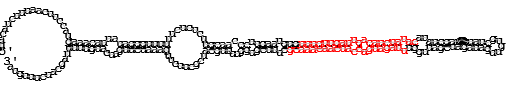 | 22 | NA |
| mol-miR858 | *mol-miR858b* | UUCGUUGUCUGUUCGACCUUG |  | 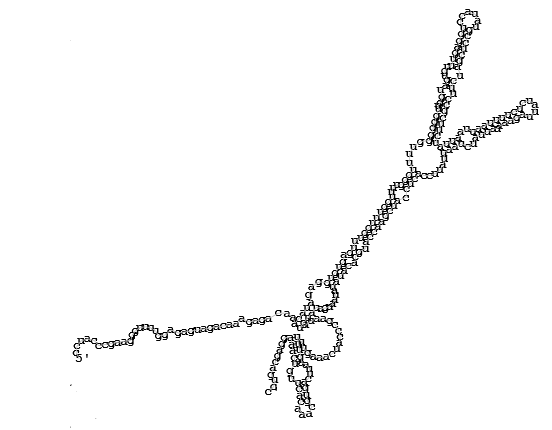 | 34 |  |
| mol-miR894 | *mol-miR894* | CGUUUCACGUCGGGUUCACC |  | 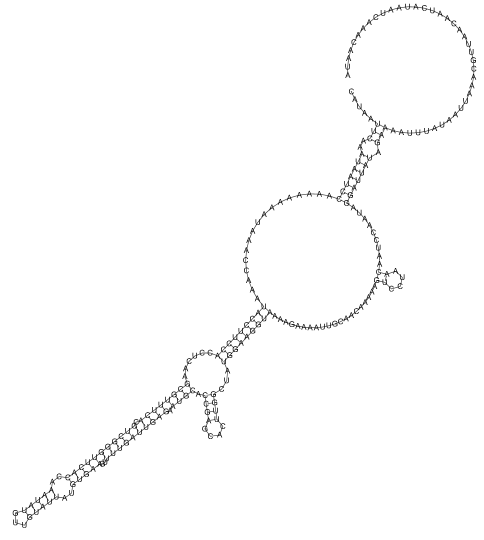 | 396 |  |
| mol-miR1310 | *mol-miR1310* | AGGCAUCGGGGGCGCAACGCCC |  | 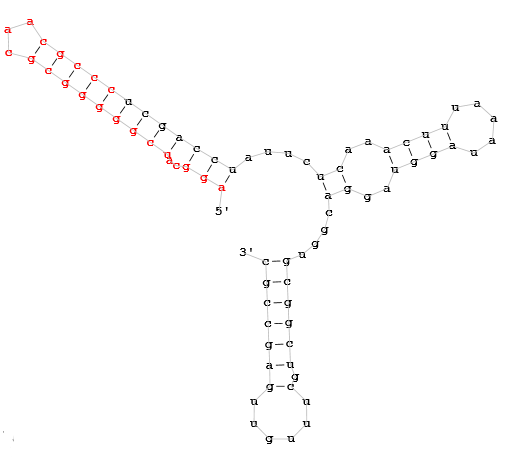 | 1170 |  |
| mol-miR1511 | *mol-miR1511* | CGUGGUAUCAGAGUCAUGUUA | ACCUGGCUCUGAUACCAUAAC | 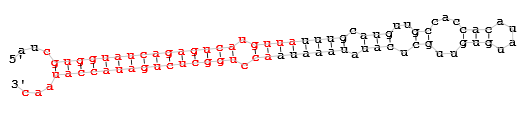 | 1279 |  |
| mol-miR1515 | *mol-miR1515* | UCAUUUUUGCGUGCAAUGAUCC |  | 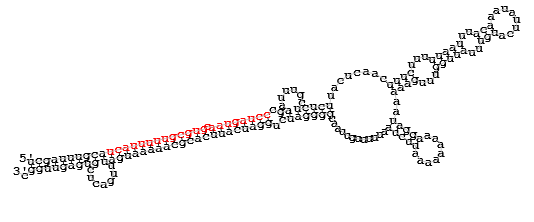 | 61 |  |
| mol-miR3711 | *mol-miR3711* | UGGCGCUAGAAGGAGGGCCU |  | 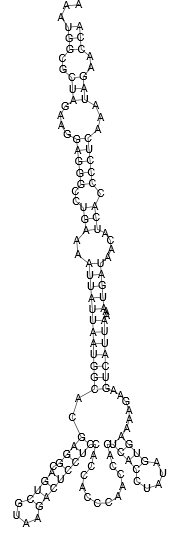 | 271 |  |
| mol-miR5139 | *mol-miR5139* | AAACCUGGCUCUGAUACCA |  | 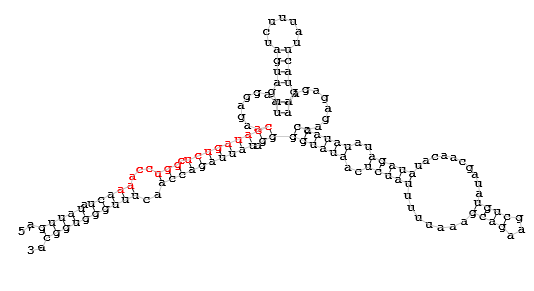 | 1612 |  |
| mol-miR5559 | *mol-miR5559* | UACUUGGUGAAUUGUUGGAUC |  | 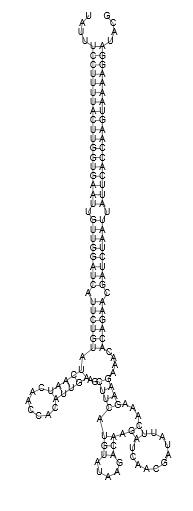 | 322 |  |
| mol-miR6300 | *mol-miR6300* | GUCGUUGUAGUAUAGUGG |  | 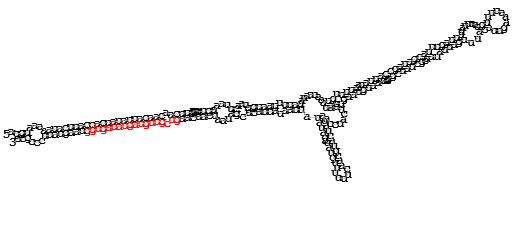 | 10523 |  |
| mol-miR6478 | *mol-miR6478* | CCGACCUUAGCUCAGUUGGUG |  | 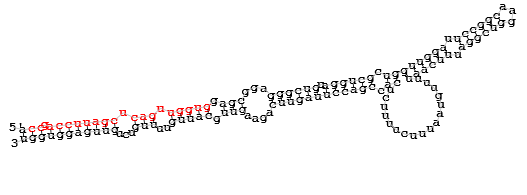 | 8520 |  |
| mol-miR7751 | *mol-miR7751* | AUCUUCCUCGUGGACAAGCGGUAG | UUUGGUGCACCCGGCUGGAGAUGG | 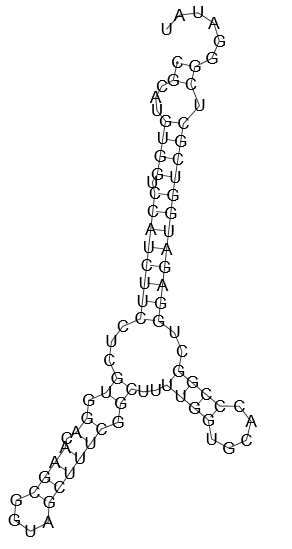 | NA | 1683 |
| mol-miR8155 | *mol-miR8155* | UAACCUGGCUCUGAUACCA |  | 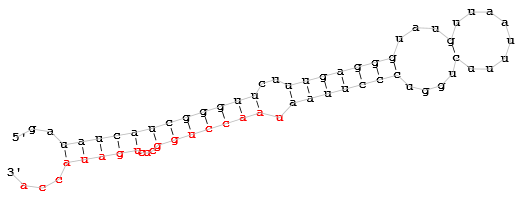 | 1612 |  |
