# Supplementary material for: MicroRNA from Moringa oleifera: Identification by High Throughput Sequencing and Their Potential Contribution to Plant Medicinal Value
Source: PLoS One. 2016 Mar 1;11(3):e0149495. doi: 10.1371/journal.pone.0149495 (PMC4773123; doi:10.1371/journal.pone.0149495)
Supplement: S2 Table — (DOCX) [file pone.0149495.s006.docx]

**S2 Table.** Complete prediction report of gene target in humans, using COMIR software.

| **miRNA name** | **Ensemble Gene ID** | **Entrez ID** | **Gene name** | **COMIR Score** |
| --- | --- | --- | --- | --- |
| ***mol-miR-166i*** | ENSG00000064393 | 28996 | *HIPK2* | 0.9076 |
|  | ENSG00000169213 | 5865 | *RAB3B* | 0.9075 |
|  | ENSG00000119547 | 9480 | *ONECUT2* | 0.9075 |
|  | ENSG00000263162 | 8924 100653292 | *HERC2* | 0.9074 |
|  | ENSG00000077279 | 1641 | *DCX* | 0.9073 |
|  | ENSG00000077157 | 4660 | *PPP1R12B* | 0.9071 |
|  | ENSG00000161791 | 91010 | *FMNL3* | 0.9071 |
|  | ENSG00000163291 | 152559 | *PAQR3* | 0.907 |
|  | ENSG00000221866 | 91584 | *PLXNA4* | 0.907 |
|  | ENSG00000112200 | 26036 | *ZNF451* | 0.9068 |
|  | ENSG00000164684 | 619279 | *ZNF704* | 0.9058 |
|  | ENSG00000131242 | 84440 | *RAB11FIP4* | 0.9058 |
|  | ENSG00000196850 | 160760 | *PPTC7* | 0.9058 |
|  | ENSG00000102908 | 10725 | *NFAT5* | 0.9057 |
|  | ENSG00000198700 | 55705 | *IPO9* | 0.9056 |
|  | ENSG00000272325 | 11165 | *NUDT3* | 0.9056 |
|  | ENSG00000120137 | 79646 | *PANK3* | 0.9055 |
|  | ENSG00000187231 | 91404 | *SESTD1* | 0.9054 |
|  | ENSG00000145725 | 23262 | *PPIP5K2* | 0.9054 |
|  | ENSG00000079950 | 8417 | *STX7* | 0.9053 |
|  | ENSG00000076641 | 55824 | *PAG1* | 0.9052 |
|  | ENSG00000110395 | 867 | *CBL* | 0.9051 |
|  | ENSG00000183826 | 114781 | *BTBD9* | 0.9051 |
|  | ENSG00000132549 | 157680 | *VPS13B* | 0.9051 |
|  | ENSG00000152104 | 5784 | *PTPN14* | 0.9051 |
|  | ENSG00000143195 | 387597 | *ILDR2* | 0.905 |
|  | ENSG00000152495 | 814 | *CAMK4* | 0.905 |
|  | ENSG00000149212 | 143686 | *SESN3* | 0.9049 |
|  | ENSG00000186432 | 3840 | *KPNA4* | 0.9049 |
|  | ENSG00000158445 | 3745 | *KCNB1* | 0.9048 |
|  | ENSG00000070214 | 23446 | *SLC44A1* | 0.9047 |
|  | ENSG00000082175 | 5241 | *PGR* | 0.9046 |
|  | ENSG00000166436 | 9866 | *TRIM66* | 0.9046 |
|  | ENSG00000148158 | 401548 | *SNX30* | 0.9046 |
|  | ENSG00000101098 | 140730 | *RIMS4* | 0.9045 |
|  | ENSG00000127124 | 59269 | *HIVEP3* | 0.9044 |
|  | ENSG00000078142 | 5289 | *PIK3C3* | 0.9044 |
|  | ENSG00000076770 | 55796 | *MBNL3* | 0.9044 |
|  | ENSG00000112419 | 9749 | *PHACTR2* | 0.9043 |
|  | ENSG00000184863 | 155435 | *RBM33* | 0.9042 |
|  | ENSG00000050030 | 340533 | *KIAA2022* | 0.904 |
|  | ENSG00000168769 | 54790 | *TET2* | 0.9039 |
|  | ENSG00000148200 | 2649 | *NR6A1* | 0.9039 |
|  | ENSG00000156113 | 3778 | *KCNMA1* | 0.9038 |
|  | ENSG00000154478 | 2849 | *GPR26* | 0.9038 |
|  | ENSG00000122359 | 311 | *ANXA11* | 0.9036 |
|  | ENSG00000214338 | 9729 387104 | *SOGA3* | 0.9036 |
|  | ENSG00000255330 | 9729 387104 | *NA* | 0.9036 |
|  | ENSG00000163492 | 285025 | *CCDC141* | 0.9034 |
|  | ENSG00000122012 | 22987 | *SV2C* | 0.9032 |
|  | ENSG00000205765 | 285636 | *C5orf51* | 0.903 |
|  | ENSG00000165185 | 158405 | *KIAA1958* | 0.9028 |
|  | ENSG00000123213 | 57486 | *NLN* | 0.9027 |
|  | ENSG00000050628 | 5733 | *PTGER3* | 0.9026 |
|  | ENSG00000128607 | 23008 | *KLHDC10* | 0.9023 |
|  | ENSG00000082701 | 2932 | *GSK3B* | 0.9019 |
|  | ENSG00000112339 | 10767 | *HBS1L* | 0.9018 |
|  | ENSG00000128567 | 5420 | *PODXL* | 0.9016 |
|  | ENSG00000171105 | 3643 | *INSR* | 0.9015 |
|  | ENSG00000108175 | 57178 | *ZMIZ1* | 0.9015 |
|  | ENSG00000043143 | 23338 | *PHF15* | 0.9014 |
|  | ENSG00000150394 | 1006 | *CDH8* | 0.9013 |
|  | ENSG00000157823 | 10239 100526783 | *AP3S2* | 0.9013 |
|  | ENSG00000164066 | 27152 | *INTU* | 0.901 |
|  | ENSG00000163281 | 132789 | *GNPDA2* | 0.9009 |
|  | ENSG00000181090 | 79813 | *EHMT1* | 0.9009 |
|  | ENSG00000100038 | 8940 | *TOP3B* | 0.9007 |
|  | ENSG00000164114 | 79884 | *MAP9* | 0.9006 |
|  | ENSG00000144036 | 23233 | *EXOC6B* | 0.9004 |
|  | ENSG00000173068 | 54796 | *BNC2* | 0.9003 |
|  | ENSG00000143469 | 255928 | *SYT14* | 0.9 |
|  | ENSG00000182378 | 55344 | *PLCXD1* | 0.9 |
| ***mol-miR-393c*** | ENSG00000178662 | 80034 | *CSRNP3* | 0.9075 |
|  | ENSG00000102908 | 10725 | *NFAT5* | 0.9074 |
|  | ENSG00000128585 | 4289 | *MKLN1* | 0.9074 |
|  | ENSG00000145907 | 10146 | *G3BP1* | 0.9074 |
|  | ENSG00000009413 | 5980 | *REV3L* | 0.9072 |
|  | ENSG00000010244 | 7756 | *ZNF207* | 0.9072 |
|  | ENSG00000100354 | 23112 | *TNRC6B* | 0.9072 |
|  | ENSG00000143190 | 5451 | *pou2f1* | 0.9072 |
|  | ENSG00000173611 | 286205 | *Scai* | 0.9072 |
|  | ENSG00000100731 | 22990 | *pcnx* | 0.9071 |
|  | ENSG00000204524 | 390980 | *ZNF805* | 0.9071 |
|  | ENSG00000109189 | 64854 | *USP46* | 0.907 |
|  | ENSG00000111728 | 6489 | *ST8SIA1* | 0.907 |
|  | ENSG00000177511 | 51046 | *ST8SIA3* | 0.907 |
|  | ENSG00000180011 | 284273 | *ZADH2* | 0.907 |
|  | ENSG00000205269 | 1E+08 | *Tmem170b* | 0.9069 |
|  | ENSG00000127328 | 117177 | *RAB3IP* | 0.9067 |
|  | ENSG00000138767 | 246175 | *CNOT6L* | 0.9067 |
|  | ENSG00000178568 | 2066 | *Erbb4* | 0.9067 |
|  | ENSG00000106701 | 83856 | *FSD1L* | 0.9064 |
|  | ENSG00000083844 | 9422 | *ZNF264* | 0.9063 |
|  | ENSG00000119547 | 9480 | *ONECUT2* | 0.9063 |
|  | ENSG00000140538 | 4916 | *NTRK3* | 0.9063 |
|  | ENSG00000145012 | 4026 | *lpp* | 0.9063 |
|  | ENSG00000165288 | 254065 | *Brwd3* | 0.9063 |
|  | ENSG00000154080 | 83539 | *Chst9* | 0.9062 |
|  | ENSG00000151422 | 2241 | *fer* | 0.906 |
|  | ENSG00000048740 | 10659 | *CELF2* | 0.9059 |
|  | ENSG00000124783 | 6745 | *SSR1* | 0.9059 |
|  | ENSG00000134852 | 9575 | *CLOCK* | 0.9059 |
|  | ENSG00000170500 | 164832 | *Lonrf2* | 0.9059 |
|  | ENSG00000168807 | 6645 | *sntb2* | 0.9058 |
|  | ENSG00000124788 | 6310 | *ATXN1* | 0.9055 |
|  | ENSG00000148053 | 4915 | *NTRK2* | 0.9055 |
|  | ENSG00000186951 | 5465 | *PPARA* | 0.9055 |
|  | ENSG00000091436 | 51776 | *ZAK* | 0.9054 |
|  | ENSG00000147036 | 347404 | *Lancl3* | 0.9053 |
|  | ENSG00000008277 | 53616 | *ADAM22* | 0.9052 |
|  | ENSG00000134352 | 3572 | *IL6ST* | 0.9052 |
|  | ENSG00000060982 | 586 | *BCAT1* | 0.9051 |
|  | ENSG00000163697 | 323 | *Apbb2* | 0.9051 |
|  | ENSG00000166342 | 81832 | *NETO1* | 0.9051 |
|  | ENSG00000176542 | 205717 | *KIAA2018* | 0.905 |
|  | ENSG00000106692 | 2218 | *FKTN* | 0.9049 |
|  | ENSG00000165186 | 139411 | *PTCHD1* | 0.9049 |
|  | ENSG00000206052 | 220164 | *Dok6* | 0.9049 |
|  | ENSG00000139132 | 121512 | *FGD4* | 0.9048 |
|  | ENSG00000102781 | 84056 | *KATNAL1* | 0.9047 |
|  | ENSG00000187118 | 152100 | *CMC1* | 0.9047 |
|  | ENSG00000111605 | 11052 | *Cpsf6* | 0.9046 |
|  | ENSG00000152409 | 133746 | *JMY* | 0.9046 |
|  | ENSG00000161791 | 91010 | *FMNL3* | 0.9045 |
|  | ENSG00000187068 | 285382 | *C3orf70* | 0.9044 |
|  | ENSG00000172795 | 167227 | *dcp2* | 0.9043 |
|  | ENSG00000103064 | 9057 | *SLC7A6* | 0.9042 |
|  | ENSG00000106771 | 23731 | *c9orf5* | 0.9042 |
|  | ENSG00000173517 | 79834 | *SGK269* | 0.9041 |
|  | ENSG00000139496 | 9818 | *nupl1* | 0.9036 |
|  | ENSG00000166225 | 10818 | *frs2* | 0.9036 |
|  | ENSG00000069667 | 6095 | *RORA* | 0.9035 |
|  | ENSG00000164199 | 84059 | *GPR98* | 0.9035 |
|  | ENSG00000175387 | 4087 | *SMAD2* | 0.9035 |
|  | ENSG00000100030 | 5594 | *MAPK1* | 0.9034 |
|  | ENSG00000107957 | 9644 | *SH3PXD2A* | 0.9034 |
|  | ENSG00000157741 | 254048 | *ubn2* | 0.9034 |
|  | ENSG00000175548 | 144245 | *ALG10B* | 0.9034 |
|  | ENSG00000188647 | 375743 | *PTAR1* | 0.9034 |
|  | ENSG00000152932 | 115827 | *Rab3c* | 0.9033 |
|  | ENSG00000169967 | 10746 | *MAP3K2* | 0.9033 |
|  | ENSG00000186472 | 27445 | *PCLO* | 0.9033 |
|  | ENSG00000108256 | 57532 | *NUFIP2* | 0.9032 |
|  | ENSG00000112902 | 9037 | *SEMA5A* | 0.9032 |
|  | ENSG00000113494 | 5618 | *PRLR* | 0.9032 |
|  | ENSG00000166128 | 51762 | *RAB8B* | 0.9032 |
|  | ENSG00000184743 | 25923 | *ATL3* | 0.9032 |
|  | ENSG00000187231 | 91404 | *Sestd1* | 0.9032 |
|  | ENSG00000198625 | 4194 | *MDM4* | 0.9032 |
|  | ENSG00000204977 | 10206 | *TRIM13* | 0.9032 |
|  | ENSG00000128573 | 93986 | *FOXP2* | 0.9031 |
|  | ENSG00000152492 | 152137 | *CCDC50* | 0.9031 |
|  | ENSG00000163848 | 7707 | *ZNF148* | 0.9031 |
|  | ENSG00000188177 | 376940 | *ZC3H6* | 0.9031 |
|  | ENSG00000048649 | 51773 | *rsf1* | 0.903 |
|  | ENSG00000078142 | 5289 | *PIK3C3* | 0.903 |
|  | ENSG00000094916 | 23468 | *CBX5* | 0.903 |
|  | ENSG00000140526 | 11057 | *ABHD2* | 0.903 |
|  | ENSG00000196090 | 11122 | *PTPRT* | 0.903 |
|  | ENSG00000182168 | 8633 | *UNC5C* | 0.9029 |
|  | ENSG00000182836 | 345557 | *PLCXD3* | 0.9029 |
|  | ENSG00000186432 | 3840 | *KPNA4* | 0.9029 |
|  | ENSG00000173744 | 3267 | *AGFG1* | 0.9028 |
|  | ENSG00000090376 | 11213 | *irak3* | 0.9027 |
|  | ENSG00000104643 | 66036 | *Mtmr9* | 0.9027 |
|  | ENSG00000167306 | 4645 | *LOC441420 LOC392335 MYO5B* | 0.9027 |
|  | ENSG00000128872 | 29767 | *tmod2* | 0.9026 |
|  | ENSG00000164164 | 54726 | *OTUD4* | 0.9026 |
|  | ENSG00000175497 | 57628 | *DPP10* | 0.9026 |
|  | ENSG00000185658 | 54014 | *BRWD1* | 0.9026 |
|  | ENSG00000109171 | 57606 | *SLAIN2* | 0.9025 |
|  | ENSG00000122591 | 84668 | *FAM126A* | 0.9025 |
|  | ENSG00000173218 | 81839 | *VANGL1* | 0.9025 |
|  | ENSG00000177853 | 9849 | *ZNF518A* | 0.9025 |
|  | ENSG00000198162 | 10905 | *MAN1A2* | 0.9025 |
|  | ENSG00000177570 | 401474 | *SAMD12* | 0.9024 |
|  | ENSG00000084093 | 5978 | *REST* | 0.9023 |
|  | ENSG00000101966 | 331 | *XIAP* | 0.9023 |
|  | ENSG00000153094 | 10018 | *BCL2L11* | 0.9023 |
|  | ENSG00000157617 | 25966 | *C2CD2* | 0.9023 |
|  | ENSG00000172403 | 171024 | *SYNPO2* | 0.9023 |
|  | ENSG00000066739 | 55102 | *ATG2B* | 0.9022 |
|  | ENSG00000118217 | 22926 | *Atf6* | 0.9021 |
|  | ENSG00000100433 | 54207 | *Kcnk10* | 0.902 |
|  | ENSG00000175414 | 285598 | *arl10* | 0.902 |
|  | ENSG00000177565 | 79718 | *TBL1XR1* | 0.902 |
|  | ENSG00000196632 | 65267 | *WNK3* | 0.902 |
|  | ENSG00000128000 | 163131 | *ZNF780B* | 0.9017 |
|  | ENSG00000174010 | 80311 | *KLHL15* | 0.9017 |
|  | ENSG00000177463 | 7182 | *NR2C2* | 0.9017 |
|  | ENSG00000180667 | 55432 | *YOD1* | 0.9017 |
|  | ENSG00000056586 | 54542 | *RC3H2* | 0.9016 |
|  | ENSG00000173578 | 2829 | *Xcr1* | 0.9016 |
|  | ENSG00000083896 | 91746 | *YTHDC1* | 0.9015 |
|  | ENSG00000175175 | 22843 | *PPM1E* | 0.9015 |
|  | ENSG00000204406 | 55777 | *MBD5* | 0.9015 |
|  | ENSG00000171885 | 361 | *AQP4* | 0.9014 |
|  | ENSG00000099725 | 5616 | *PRKY* | 0.9013 |
|  | ENSG00000101290 | 8760 | *Cds2* | 0.9013 |
|  | ENSG00000162433 | 205 | *AK3L2 AK3L1* | 0.9013 |
|  | ENSG00000185219 | 353274 | *ZNF445* | 0.9013 |
|  | ENSG00000003989 | 6542 | *SLC7A2* | 0.9012 |
|  | ENSG00000102910 | 83752 | *LONP2* | 0.9012 |
|  | ENSG00000138735 | 8654 | *PDE5A* | 0.9012 |
|  | ENSG00000116678 | 3953 | *LEPR* | 0.9011 |
|  | ENSG00000197323 | 51592 | *trim33* | 0.9011 |
|  | ENSG00000154429 | 126731 | *C1orf96* | 0.901 |
|  | ENSG00000145819 | 23092 | *arhgap26* | 0.9009 |
|  | ENSG00000186260 | 57496 | *MKL2* | 0.9009 |
|  | ENSG00000139160 | 254013 | *C12orf72* | 0.9008 |
|  | ENSG00000008294 | 9043 | *Spag9* | 0.9007 |
|  | ENSG00000135316 | 10492 | *SYNCRIP* | 0.9007 |
|  | ENSG00000164114 | 79884 | *MAP9* | 0.9007 |
|  | ENSG00000151151 | 253430 | *IPMK* | 0.9006 |
|  | ENSG00000173166 | 65059 | *Raph1* | 0.9006 |
|  | ENSG00000174804 | 8322 | *FZD4* | 0.9006 |
|  | ENSG00000198948 | 9848 | *MFAP3L* | 0.9006 |
|  | ENSG00000040199 | 23035 | *PHLPP2* | 0.9004 |
|  | ENSG00000092978 | 55105 | *gpatch2* | 0.9004 |
|  | ENSG00000152377 | 6695 | *Spock1* | 0.9004 |
|  | ENSG00000165434 | 283209 | *PGM2L1* | 0.9004 |
|  | ENSG00000161048 | 222236 | *NAPEPLD* | 0.9003 |
|  | ENSG00000175329 | 91464 | *ISX* | 0.9003 |
|  | ENSG00000127334 | 8445 | *dyrk2* | 0.9002 |
|  | ENSG00000183826 | 114781 | *BTBD9* | 0.9002 |
|  | ENSG00000006468 | 2115 | *etv1* | 0.9001 |
|  | ENSG00000114331 | 23527 | *ACAP2* | 0.9001 |
|  | ENSG00000127329 | 5787 | *PTPRB* | 0.9001 |
|  | ENSG00000188227 | 390927 | *ZNF793* | 0.9001 |
|  | ENSG00000197381 | 104 | *Adarb1* | 0.9001 |
| ***mol-miR-159a*** | ENSG00000171435 | 283455 | *KSR2* | 0.9076 |
|  | ENSG00000119547 | 9480 | *ONECUT2* | 0.9076 |
|  | ENSG00000153721 | 154043 | *CNKSR3* | 0.9074 |
|  | ENSG00000261115 | 1.01E+08 | *TMEM178B* | 0.9074 |
|  | ENSG00000158445 | 3745 | *KCNB1* | 0.9072 |
|  | ENSG00000077157 | 4660 | *PPP1R12B* | 0.907 |
|  | ENSG00000196090 | 11122 | *PTPRT* | 0.907 |
|  | ENSG00000055609 | 58508 | *KMT2C* | 0.9068 |
|  | ENSG00000132549 | 157680 | *VPS13B* | 0.9065 |
|  | ENSG00000148019 | 84131 | *CEP78* | 0.9062 |
|  | ENSG00000110436 | 6506 | *SLC1A2* | 0.9062 |
|  | ENSG00000161791 | 91010 | *FMNL3* | 0.9062 |
|  | ENSG00000153404 | 153478 | *PLEKHG4B* | 0.906 |
|  | ENSG00000185621 | 89782 | *LMLN* | 0.906 |
|  | ENSG00000055332 | 5610 | *EIF2AK2* | 0.9059 |
|  | ENSG00000141622 | 494470 | *RNF165* | 0.9059 |
|  | ENSG00000051620 | 23593 | *HEBP2* | 0.9057 |
|  | ENSG00000069702 | 7049 | *TGFBR3* | 0.9053 |
|  | ENSG00000124788 | 6310 | *ATXN1* | 0.9053 |
|  | ENSG00000114857 | 4820 | *NKTR* | 0.9052 |
|  | ENSG00000175387 | 4087 | *SMAD2* | 0.905 |
|  | ENSG00000185518 | 9899 | *SV2B* | 0.9047 |
|  | ENSG00000206052 | 220164 | *DOK6* | 0.9047 |
|  | ENSG00000255833 | 497189 | *TIFAB* | 0.9045 |
|  | ENSG00000035664 | 23604 | *DAPK2* | 0.904 |
|  | ENSG00000164663 | 25862 | *USP49* | 0.9039 |
|  | ENSG00000165238 | 65268 | *WNK2* | 0.9034 |
|  | ENSG00000122733 | 23349 | *KIAA1045* | 0.9033 |
|  | ENSG00000125686 | 5469 | *MED1* | 0.9029 |
|  | ENSG00000114416 | 8087 | *FXR1* | 0.9026 |
|  | ENSG00000001497 | 81887 | *LAS1L* | 0.9025 |
|  | ENSG00000157423 | 54768 100288805 101060699 | *HYDIN* | 0.9024 |
|  | ENSG00000250091 | NA | *DNAH10OS* | 0.9024 |
|  | ENSG00000092871 | 117584 | *RFFL* | 0.9023 |
|  | ENSG00000170456 | 160518 | *DENND5B* | 0.9022 |
|  | ENSG00000128487 | 92521 | *SPECC1* | 0.902 |
|  | ENSG00000171914 | 83660 | *TLN2* | 0.9018 |
|  | ENSG00000183833 | 89876 | *MAATS1* | 0.9018 |
|  | ENSG00000183715 | 4978 | *OPCML* | 0.9018 |
|  | ENSG00000135074 | 8728 | *ADAM19* | 0.9014 |
|  | ENSG00000143190 | 5451 | *POU2F1* | 0.9014 |
|  | ENSG00000102908 | 10725 | *NFAT5* | 0.9014 |
|  | ENSG00000140538 | 4916 | *NTRK3* | 0.9013 |
|  | ENSG00000138185 | 953 | *ENTPD1* | 0.9013 |
|  | ENSG00000149639 | 140710 | *SOGA1* | 0.9012 |
|  | ENSG00000133019 | 1131 | *CHRM3* | 0.9011 |
|  | ENSG00000198216 | 777 | *CACNA1E* | 0.9011 |
|  | ENSG00000106789 | 7464 | *CORO2A* | 0.9011 |
|  | ENSG00000269216 | 8218 | *NA* | 0.9011 |
|  | ENSG00000164742 | 107 | *ADCY1* | 0.9009 |
|  | ENSG00000170624 | 6444 | *SGCD* | 0.9009 |
|  | ENSG00000221866 | 91584 | *PLXNA4* | 0.9008 |
|  | ENSG00000183166 | 83698 | *CALN1* | 0.9008 |
|  | ENSG00000188859 | 149297 | *FAM78B* | 0.9008 |
|  | ENSG00000068654 | 25885 | *POLR1A* | 0.9007 |
|  | ENSG00000196092 | 5079 | *PAX5* | 0.9007 |
|  | ENSG00000113494 | 5618 | *PRLR* | 0.9005 |
|  | ENSG00000164684 | 619279 | *ZNF704* | 0.9005 |
|  | ENSG00000139767 | 84530 | *SRRM4* | 0.9004 |
|  | ENSG00000151338 | 145282 | *MIPOL1* | 0.9004 |
|  | ENSG00000162849 | 55083 | *KIF26B* | 0.9004 |
|  | ENSG00000178033 | 254228 | *FAM26E* | 0.9003 |
|  | ENSG00000160551 | 57551 | *TAOK1* | 0.9002 |
|  | ENSG00000153029 | 3140 | *MR1* | 0.9002 |
|  | ENSG00000165185 | 158405 | *KIAA1958* | 0.9001 |
|  | ENSG00000006432 | 4293 | *MAP3K9* | 0.9001 |
|  | ENSG00000117597 | 27042 | *DIEXF* | 0.9001 |
|  | ENSG00000036530 | 10858 | *CYP46A1* | 0.9 |
|  | ENSG00000146414 | 257218 | *SHPRH* | 0.9 |
|  | ENSG00000117036 | 2117 | *ETV3* | 0.9 |
|  | ENSG00000010292 | 9918 | *NCAPD2* | 0.9 |
|  | ENSG00000183049 | 57118 | *CAMK1D* | 0.9 |
| ***mol-miR-6478*** | ENSG00000134313 | 57498 | *KIDINS220* | 0.9061 |
|  | ENSG00000106261 | 7586 | *ZKSCAN1* | 0.9058 |
|  | ENSG00000134909 | 9743 | *ARHGAP32* | 0.9055 |
|  | ENSG00000136709 | 55339 84826 | *WDR33* | 0.9055 |
|  | ENSG00000112706 | 3617 | *IMPG1* | 0.9051 |
|  | ENSG00000107331 | 20 | *ABCA2* | 0.9049 |
|  | ENSG00000088808 | 23368 | *PPP1R13B* | 0.9029 |
|  | ENSG00000167654 | 85300 | *ATCAY* | 0.9028 |
|  | ENSG00000189339 | 728661 | *SLC35E2B* | 0.9026 |
|  | ENSG00000180370 | 5062 | *PAK2* | 0.9025 |
|  | ENSG00000198000 | 55035 | *NOL8* | 0.9023 |
|  | ENSG00000183496 | 84206 | *MEX3B* | 0.9021 |
|  | ENSG00000055163 | 26999 | *CYFIP2* | 0.9016 |
|  | ENSG00000162337 | 4041 | *LRP5* | 0.9004 |
|  | ENSG00000112655 | 5754 | *PTK7* | 0.9001 |
| ***mol-miR-6300*** | ENSG00000178567 | 9852 | *EPM2AIP1* | 0.9076 |
|  | ENSG00000213699 | 54978 | *SLC35F6* | 0.9075 |
|  | ENSG00000197818 | 23315 | *SLC9A8* | 0.9072 |
|  | ENSG00000183751 | 10607 | *TBL3* | 0.9071 |
|  | ENSG00000206190 | 57194 | *ATP10A* | 0.9071 |
|  | ENSG00000166206 | 2562 | *GABRB3* | 0.9071 |
|  | ENSG00000198000 | 55035 | *NOL8* | 0.907 |
|  | ENSG00000172380 | 55970 | *GNG12* | 0.907 |
|  | ENSG00000152443 | 284309 | *ZNF776* | 0.9067 |
|  | ENSG00000133703 | 3845 | *KRAS* | 0.9066 |
|  | ENSG00000057252 | 6646 | *SOAT1* | 0.9065 |
|  | ENSG00000103342 | 2935 | *GSPT1* | 0.9065 |
|  | ENSG00000137502 | 27314 | *RAB30* | 0.9064 |
|  | ENSG00000179532 | 144132 | *DNHD1* | 0.9064 |
|  | ENSG00000140557 | 8128 | *ST8SIA2* | 0.9064 |
|  | ENSG00000214688 | 414152 | *C10orf105* | 0.9063 |
|  | ENSG00000162413 | 9903 | *KLHL21* | 0.9062 |
|  | ENSG00000139163 | 55500 | *ETNK1* | 0.9062 |
|  | ENSG00000152700 | 51128 | *SAR1B* | 0.9062 |
|  | ENSG00000257950 | NA | *NA* | 0.9061 |
|  | ENSG00000009765 | 389434 | *IYD* | 0.9061 |
|  | ENSG00000168056 | 4054 | *LTBP3* | 0.9061 |
|  | ENSG00000100413 | 171568 | *POLR3H* | 0.9061 |
|  | ENSG00000171385 | 3752 | *KCND3* | 0.9061 |
|  | ENSG00000148848 | 8038 | *ADAM12* | 0.9061 |
|  | ENSG00000130477 | 23025 | *UNC13A* | 0.906 |
|  | ENSG00000256062 | 28 | *ABO* | 0.906 |
|  | ENSG00000185532 | 5592 | *PRKG1* | 0.906 |
|  | ENSG00000122692 | 55234 | *SMU1* | 0.9058 |
|  | ENSG00000125447 | 23163 | *GGA3* | 0.9057 |
|  | ENSG00000156052 | 2776 | *GNAQ* | 0.9057 |
|  | ENSG00000105227 | 57716 | *PRX* | 0.9057 |
|  | ENSG00000108107 | 6158 | *RPL28* | 0.9056 |
|  | ENSG00000197183 | 140688 | *C20orf112* | 0.9056 |
|  | ENSG00000186866 | 23275 | *POFUT2* | 0.9055 |
|  | ENSG00000113361 | 1004 | *CDH6* | 0.9054 |
|  | ENSG00000162378 | 79699 | *ZYG11B* | 0.9054 |
|  | ENSG00000176274 | 401612 | *SLC25A53* | 0.9053 |
|  | ENSG00000187627 | 400966 | *RGPD1* | 0.9053 |
|  | ENSG00000186174 | 283149 | *BCL9L* | 0.9049 |
|  | ENSG00000147459 | 80005 | *DOCK5* | 0.9049 |
|  | ENSG00000153246 | 22925 | *PLA2R1* | 0.9049 |
|  | ENSG00000186260 | 57496 | *MKL2* | 0.9049 |
|  | ENSG00000169247 | 79628 | *SH3TC2* | 0.9049 |
|  | ENSG00000087266 | 6452 | *SH3BP2* | 0.9048 |
|  | ENSG00000158985 | 56990 | *CDC42SE2* | 0.9048 |
|  | ENSG00000269743 | 401612 | *NA* | 0.9047 |
|  | ENSG00000163728 | 151613 | *TTC14* | 0.9046 |
|  | ENSG00000175390 | 8665 | *EIF3F* | 0.9045 |
|  | ENSG00000197323 | 51592 | *TRIM33* | 0.9044 |
|  | ENSG00000116852 | 23046 | *KIF21B* | 0.9043 |
|  | ENSG00000160352 | 148206 | *ZNF714* | 0.9043 |
|  | ENSG00000181634 | 9966 | *TNFSF15* | 0.9043 |
|  | ENSG00000261772 | 440073 100996825 | *IQSEC3* | 0.9043 |
|  | ENSG00000125246 | 171425 | *CLYBL* | 0.904 |
|  | ENSG00000162337 | 4041 | *LRP5* | 0.9039 |
|  | ENSG00000113580 | 2908 | *NR3C1* | 0.9039 |
|  | ENSG00000141622 | 494470 | *RNF165* | 0.9038 |
|  | ENSG00000110693 | 55553 | *SOX6* | 0.9038 |
|  | ENSG00000105793 | 85865 | *GTPBP10* | 0.9038 |
|  | ENSG00000182372 | 2055 | *CLN8* | 0.9038 |
|  | ENSG00000177000 | 4524 | *MTHFR* | 0.9038 |
|  | ENSG00000120645 | 440073 100996825 | *IQSEC3* | 0.9037 |
|  | ENSG00000262607 | 440073 100996825 | *IQSEC3* | 0.9037 |
|  | ENSG00000100077 | 157 | *ADRBK2* | 0.9037 |
|  | ENSG00000015153 | 10138 | *YAF2* | 0.9037 |
|  | ENSG00000271374 | 23241 | *PACS2* | 0.9037 |
|  | ENSG00000134780 | 747 | *DAGLA* | 0.9036 |
|  | ENSG00000170584 | 134492 | *NUDCD2* | 0.9036 |
|  | ENSG00000169891 | 9185 | *REPS2* | 0.9036 |
|  | ENSG00000144320 | 80856 | *KIAA1715* | 0.9035 |
|  | ENSG00000197134 | 113835 | *ZNF257* | 0.9034 |
|  | ENSG00000160209 | 8566 | *PDXK* | 0.9034 |
|  | ENSG00000179364 | 23241 | *PACS2* | 0.9034 |
|  | ENSG00000153029 | 3140 | *MR1* | 0.9034 |
|  | ENSG00000154122 | 56172 | *ANKH* | 0.9034 |
|  | ENSG00000151834 | 2555 | *GABRA2* | 0.9034 |
|  | ENSG00000196628 | 6925 | *TCF4* | 0.9034 |
|  | ENSG00000129159 | 3746 | *KCNC1* | 0.9034 |
|  | ENSG00000184005 | 256435 | *ST6GALNAC3* | 0.9034 |
|  | ENSG00000171992 | 11346 | *SYNPO* | 0.9034 |
|  | ENSG00000070413 | 9993 | *DGCR2* | 0.9033 |
|  | ENSG00000134982 | 324 | *APC* | 0.9033 |
|  | ENSG00000166025 | 154810 | *AMOTL1* | 0.9033 |
|  | ENSG00000122863 | 9469 | *CHST3* | 0.9033 |
|  | ENSG00000188177 | 376940 | *ZC3H6* | 0.9033 |
|  | ENSG00000184863 | 155435 | *RBM33* | 0.9033 |
|  | ENSG00000143195 | 387597 | *ILDR2* | 0.9033 |
|  | ENSG00000168769 | 54790 | *TET2* | 0.9033 |
|  | ENSG00000162512 | 9672 | *SDC3* | 0.9032 |
|  | ENSG00000182389 | 785 | *CACNB4* | 0.9032 |
|  | ENSG00000140526 | 11057 | *ABHD2* | 0.9032 |
|  | ENSG00000179583 | 4261 | *CIITA* | 0.9031 |
|  | ENSG00000180628 | 84333 | *PCGF5* | 0.9031 |
|  | ENSG00000113448 | 5144 | *PDE4D* | 0.9031 |
|  | ENSG00000028277 | 5452 | *POU2F2* | 0.9031 |
|  | ENSG00000108854 | 64750 | *SMURF2* | 0.903 |
|  | ENSG00000171862 | 5728 | *PTEN* | 0.9029 |
|  | ENSG00000262909 | 64750 | *SMURF2* | 0.9029 |
|  | ENSG00000262023 | 8629 | *JRK* | 0.9029 |
|  | ENSG00000145911 | 23138 | *N4BP3* | 0.9029 |
|  | ENSG00000250305 | 57604 | *KIAA1456* | 0.9029 |
|  | ENSG00000148942 | 159963 | *SLC5A12* | 0.9028 |
|  | ENSG00000168939 | 10251 | *SPRY3* | 0.9028 |
|  | ENSG00000175215 | 10106 | *CTDSP2* | 0.9028 |
|  | ENSG00000196739 | 85301 | *COL27A1* | 0.9028 |
|  | ENSG00000105810 | 1021 | *CDK6* | 0.9027 |
|  | ENSG00000104343 | 55284 | *UBE2W* | 0.9027 |
|  | ENSG00000188848 | 389206 | *BEND4* | 0.9027 |
|  | ENSG00000206530 | 55779 | *WDR52* | 0.9027 |
|  | ENSG00000135870 | 149041 | *RC3H1* | 0.9026 |
|  | ENSG00000026652 | 56895 | *AGPAT4* | 0.9026 |
|  | ENSG00000141905 | 4782 | *NFIC* | 0.9026 |
|  | ENSG00000162849 | 55083 | *KIF26B* | 0.9026 |
|  | ENSG00000185219 | 353274 | *ZNF445* | 0.9026 |
|  | ENSG00000213949 | 3672 | *ITGA1* | 0.9026 |
|  | ENSG00000078177 | 55728 | *N4BP2* | 0.9025 |
|  | ENSG00000156239 | 29104 | *N6AMT1* | 0.9025 |
|  | ENSG00000106261 | 7586 | *ZKSCAN1* | 0.9025 |
|  | ENSG00000134352 | 3572 | *IL6ST* | 0.9024 |
|  | ENSG00000248905 | 342184 | *FMN1* | 0.9024 |
|  | ENSG00000066294 | 8832 | *CD84* | 0.9024 |
|  | ENSG00000175161 | 253559 | *CADM2* | 0.9023 |
|  | ENSG00000127328 | 117177 | *RAB3IP* | 0.9023 |
|  | ENSG00000170006 | 201799 | *TMEM154* | 0.9023 |
|  | ENSG00000177511 | 51046 | *ST8SIA3* | 0.9022 |
|  | ENSG00000145687 | 23635 | *SSBP2* | 0.9022 |
|  | ENSG00000128573 | 93986 | *FOXP2* | 0.9021 |
|  | ENSG00000214338 | 9729 387104 | *SOGA3* | 0.9021 |
|  | ENSG00000255330 | 9729 387104 | *NA* | 0.9021 |
|  | ENSG00000186575 | 4771 | *NF2* | 0.902 |
|  | ENSG00000120137 | 79646 | *PANK3* | 0.902 |
|  | ENSG00000175387 | 4087 | *SMAD2* | 0.902 |
|  | ENSG00000163291 | 152559 | *PAQR3* | 0.9019 |
|  | ENSG00000156103 | 4325 | *MMP16* | 0.9019 |
|  | ENSG00000185630 | 5087 | *PBX1* | 0.9019 |
|  | ENSG00000009413 | 5980 | *REV3L* | 0.9018 |
|  | ENSG00000196591 | 3066 | *HDAC2* | 0.9018 |
|  | ENSG00000103197 | 7249 | *TSC2* | 0.9017 |
|  | ENSG00000068724 | 57217 | *TTC7A* | 0.9017 |
|  | ENSG00000116260 | 5768 | *QSOX1* | 0.9017 |
|  | ENSG00000173230 | 2804 | *GOLGB1* | 0.9017 |
|  | ENSG00000079950 | 8417 | *STX7* | 0.9017 |
|  | ENSG00000225190 | 9842 | *PLEKHM1* | 0.9016 |
|  | ENSG00000022567 | 57210 | *SLC45A4* | 0.9016 |
|  | ENSG00000143190 | 5451 | *POU2F1* | 0.9016 |
|  | ENSG00000146414 | 257218 | *SHPRH* | 0.9016 |
|  | ENSG00000145012 | 4026 | *LPP* | 0.9016 |
|  | ENSG00000196526 | 60312 | *AFAP1* | 0.9015 |
|  | ENSG00000115112 | 29842 | *TFCP2L1* | 0.9015 |
|  | ENSG00000165959 | 79789 | *CLMN* | 0.9015 |
|  | ENSG00000164855 | 202915 | *TMEM184A* | 0.9015 |
|  | ENSG00000164742 | 107 | *ADCY1* | 0.9015 |
|  | ENSG00000187902 | 729956 | *SHISA7* | 0.9014 |
|  | ENSG00000164916 | 221937 | *FOXK1* | 0.9014 |
|  | ENSG00000064393 | 28996 | *HIPK2* | 0.9013 |
|  | ENSG00000116667 | 81563 | *C1orf21* | 0.9012 |
|  | ENSG00000149575 | 6327 | *SCN2B* | 0.9012 |
|  | ENSG00000128915 | 79664 | *NARG2* | 0.9012 |
|  | ENSG00000196535 | 399687 | *MYO18A* | 0.9012 |
|  | ENSG00000183354 | 158358 | *KIAA2026* | 0.9012 |
|  | ENSG00000263162 | 8924 100653292 | *HERC2* | 0.9011 |
|  | ENSG00000169905 | 163590 | *TOR1AIP2* | 0.9011 |
|  | ENSG00000114735 | 51409 | *HEMK1* | 0.9011 |
|  | ENSG00000165288 | 254065 | *BRWD3* | 0.9011 |
|  | ENSG00000107331 | 20 | *ABCA2* | 0.901 |
|  | ENSG00000235568 | 150372 | *NFAM1* | 0.901 |
|  | ENSG00000224470 | 342371 | *ATXN1L* | 0.901 |
|  | ENSG00000146350 | 221322 | *TBC1D32* | 0.901 |
|  | ENSG00000182218 | 84439 | *HHIPL1* | 0.901 |
|  | ENSG00000117016 | 9783 | *RIMS3* | 0.9009 |
|  | ENSG00000137413 | 129685 | *TAF8* | 0.9009 |
|  | ENSG00000169926 | 51621 | *KLF13* | 0.9009 |
|  | ENSG00000197779 | 347344 | *ZNF81* | 0.9009 |
|  | ENSG00000269594 | 347344 | *ZNF81* | 0.9009 |
|  | ENSG00000083312 | 3842 | *TNPO1* | 0.9009 |
|  | ENSG00000013288 | 23324 | *MAN2B2* | 0.9009 |
|  | ENSG00000171208 | 81831 | *NETO2* | 0.9008 |
|  | ENSG00000106460 | 54664 | *TMEM106B* | 0.9008 |
|  | ENSG00000131242 | 84440 | *RAB11FIP4* | 0.9008 |
|  | ENSG00000107864 | 22849 | *CPEB3* | 0.9008 |
|  | ENSG00000185838 | 54584 | *GNB1L* | 0.9008 |
|  | ENSG00000188039 | 284434 | *NWD1* | 0.9007 |
|  | ENSG00000196092 | 5079 | *PAX5* | 0.9007 |
|  | ENSG00000076641 | 55824 | *PAG1* | 0.9007 |
|  | ENSG00000148516 | 6935 100996668 | *ZEB1* | 0.9006 |
|  | ENSG00000167378 | 126298 | *IRGQ* | 0.9006 |
|  | ENSG00000164366 | 133957 | *CCDC127* | 0.9006 |
|  | ENSG00000185920 | 5727 | *PTCH1* | 0.9006 |
|  | ENSG00000107957 | 9644 | *SH3PXD2A* | 0.9005 |
|  | ENSG00000188735 | 144404 | *TMEM120B* | 0.9005 |
|  | ENSG00000203685 | 375057 | *C1orf95* | 0.9005 |
|  | ENSG00000158445 | 3745 | *KCNB1* | 0.9005 |
|  | ENSG00000036530 | 10858 | *CYP46A1* | 0.9005 |
|  | ENSG00000163873 | 2899 | *GRIK3* | 0.9005 |
|  | ENSG00000196581 | 55966 | *AJAP1* | 0.9004 |
|  | ENSG00000167291 | 125058 | *TBC1D16* | 0.9004 |
|  | ENSG00000165997 | 221079 | *ARL5B* | 0.9004 |
|  | ENSG00000135835 | 57710 | *KIAA1614* | 0.9003 |
|  | ENSG00000138185 | 953 | *ENTPD1* | 0.9003 |
|  | ENSG00000092847 | 26523 | *AGO1* | 0.9003 |
|  | ENSG00000055609 | 58508 | *KMT2C* | 0.9003 |
|  | ENSG00000269891 | 84986 | *ARHGAP19-SLIT1* | 0.9003 |
|  | ENSG00000100106 | 11078 | *TRIOBP* | 0.9002 |
|  | ENSG00000169213 | 5865 | *RAB3B* | 0.9002 |
|  | ENSG00000136279 | 28988 | *DBNL* | 0.9002 |
|  | ENSG00000089916 | 55668 | *GPATCH2L* | 0.9002 |
|  | ENSG00000119547 | 9480 | *ONECUT2* | 0.9001 |
|  | ENSG00000153404 | 153478 | *PLEKHG4B* | 0.9001 |
|  | ENSG00000140538 | 4916 | *NTRK3* | 0.9001 |
|  | ENSG00000160991 | 80228 | *ORAI2* | 0.9001 |
|  | ENSG00000145725 | 23262 | *PPIP5K2* | 0.9 |
| ***mol-miR-398c*** | ENSG00000055609 | 58508 | *KMT2C* | 0.9076 |
|  | ENSG00000164684 | 619279 | *ZNF704* | 0.9076 |
|  | ENSG00000145012 | 4026 | *LPP* | 0.9076 |
|  | ENSG00000151914 | 667 | *DST* | 0.9075 |
|  | ENSG00000158258 | 64084 | *CLSTN2* | 0.9074 |
|  | ENSG00000110436 | 6506 | *SLC1A2* | 0.9073 |
|  | ENSG00000064393 | 28996 | *HIPK2* | 0.9073 |
|  | ENSG00000118482 | 23469 | *PHF3* | 0.907 |
|  | ENSG00000143970 | 55252 | *ASXL2* | 0.907 |
|  | ENSG00000135968 | 9648 | *GCC2* | 0.907 |
|  | ENSG00000111816 | 2444 | *FRK* | 0.9068 |
|  | ENSG00000146414 | 257218 | *SHPRH* | 0.9067 |
|  | ENSG00000145725 | 23262 | *PPIP5K2* | 0.9067 |
|  | ENSG00000132549 | 157680 | *VPS13B* | 0.9067 |
|  | ENSG00000079950 | 8417 | *STX7* | 0.9067 |
|  | ENSG00000102908 | 10725 | *NFAT5* | 0.9066 |
|  | ENSG00000170006 | 201799 | *TMEM154* | 0.9065 |
|  | ENSG00000214338 | 9729 387104 | *SOGA3* | 0.9065 |
|  | ENSG00000184156 | 3786 | *KCNQ3* | 0.9065 |
|  | ENSG00000255330 | 9729 387104 | *NA* | 0.9065 |
|  | ENSG00000261115 | 1.01E+08 | *TMEM178B* | 0.9065 |
|  | ENSG00000196535 | 399687 | *MYO18A* | 0.9063 |
|  | ENSG00000177511 | 51046 | *ST8SIA3* | 0.9062 |
|  | ENSG00000069667 | 6095 | *RORA* | 0.9061 |
|  | ENSG00000151338 | 145282 | *MIPOL1* | 0.906 |
|  | ENSG00000196591 | 3066 | *HDAC2* | 0.906 |
|  | ENSG00000164916 | 221937 | *FOXK1* | 0.9058 |
|  | ENSG00000143195 | 387597 | *ILDR2* | 0.9057 |
|  | ENSG00000138944 | 85352 | *KIAA1644* | 0.9057 |
|  | ENSG00000146676 | 5814 | *PURB* | 0.9056 |
|  | ENSG00000169967 | 10746 | *MAP3K2* | 0.9055 |
|  | ENSG00000138185 | 953 | *ENTPD1* | 0.9055 |
|  | ENSG00000250305 | 57604 | *KIAA1456* | 0.9054 |
|  | ENSG00000156802 | 29028 | *ATAD2* | 0.9053 |
|  | ENSG00000065534 | 4638 | *MYLK* | 0.9053 |
|  | ENSG00000122545 | 989 | *Sep-07* | 0.9052 |
|  | ENSG00000041353 | 5874 | *RAB27B* | 0.9051 |
|  | ENSG00000122641 | 3624 | *INHBA* | 0.905 |
|  | ENSG00000163808 | 56992 | *KIF15* | 0.905 |
|  | ENSG00000139278 | 11010 | *GLIPR1* | 0.9048 |
|  | ENSG00000169905 | 163590 | *TOR1AIP2* | 0.9048 |
|  | ENSG00000110888 | 65981 | *CAPRIN2* | 0.9047 |
|  | ENSG00000145087 | 9515 | *STXBP5L* | 0.9047 |
|  | ENSG00000182197 | 2131 | *EXT1* | 0.9047 |
|  | ENSG00000183833 | 89876 | *MAATS1* | 0.9045 |
|  | ENSG00000100354 | 23112 | *TNRC6B* | 0.9044 |
|  | ENSG00000173230 | 2804 | *GOLGB1* | 0.9044 |
|  | ENSG00000153721 | 154043 | *CNKSR3* | 0.9044 |
|  | ENSG00000094916 | 23468 | *CBX5* | 0.9043 |
|  | ENSG00000152495 | 814 | *CAMK4* | 0.9043 |
|  | ENSG00000076770 | 55796 | *MBNL3* | 0.9043 |
|  | ENSG00000146373 | 154214 | *RNF217* | 0.9042 |
|  | ENSG00000169213 | 5865 | *RAB3B* | 0.9042 |
|  | ENSG00000272325 | 11165 | *NUDT3* | 0.9042 |
|  | ENSG00000163960 | 26043 | *UBXN7* | 0.9041 |
|  | ENSG00000166450 | 283659 | *PRTG* | 0.904 |
|  | ENSG00000205726 | 6453 | *ITSN1* | 0.904 |
|  | ENSG00000173068 | 54796 | *BNC2* | 0.904 |
|  | ENSG00000065060 | 54887 | *UHRF1BP1* | 0.9039 |
|  | ENSG00000113494 | 5618 | *PRLR* | 0.9039 |
|  | ENSG00000124783 | 6745 | *SSR1* | 0.9039 |
|  | ENSG00000178568 | 2066 | *ERBB4* | 0.9039 |
|  | ENSG00000146267 | 84553 | *FAXC* | 0.9039 |
|  | ENSG00000114933 | 54891 | *INO80D* | 0.9039 |
|  | ENSG00000111728 | 6489 | *ST8SIA1* | 0.9038 |
|  | ENSG00000153404 | 153478 | *PLEKHG4B* | 0.9038 |
|  | ENSG00000162599 | 4774 | *NFIA* | 0.9038 |
|  | ENSG00000166750 | 162394 | *SLFN5* | 0.9037 |
|  | ENSG00000203668 | 1122 | *CHML* | 0.9037 |
|  | ENSG00000076641 | 55824 | *PAG1* | 0.9036 |
|  | ENSG00000036530 | 10858 | *CYP46A1* | 0.9036 |
|  | ENSG00000154229 | 5578 | *PRKCA* | 0.9036 |
|  | ENSG00000128573 | 93986 | *FOXP2* | 0.9036 |
|  | ENSG00000119900 | 79627 | *OGFRL1* | 0.9036 |
|  | ENSG00000104290 | 7976 | *FZD3* | 0.9035 |
|  | ENSG00000128872 | 29767 | *TMOD2* | 0.9035 |
|  | ENSG00000143324 | 9213 | *XPR1* | 0.9035 |
|  | ENSG00000168675 | 753 | *LDLRAD4* | 0.9035 |
|  | ENSG00000006468 | 2115 | *ETV1* | 0.9034 |
|  | ENSG00000138767 | 246175 | *CNOT6L* | 0.9034 |
|  | ENSG00000147488 | 9705 | *ST18* | 0.9034 |
|  | ENSG00000118922 | 11278 | *KLF12* | 0.9034 |
|  | ENSG00000106692 | 2218 | *FKTN* | 0.9033 |
|  | ENSG00000148516 | 6935 100996668 | *ZEB1* | 0.9033 |
|  | ENSG00000073803 | 9175 | *MAP3K13* | 0.9032 |
|  | ENSG00000101290 | 8760 | *CDS2* | 0.9032 |
|  | ENSG00000161791 | 91010 | *FMNL3* | 0.9031 |
|  | ENSG00000012048 | 672 | *BRCA1* | 0.9031 |
|  | ENSG00000133019 | 1131 | *CHRM3* | 0.903 |
|  | ENSG00000132932 | 51761 | *ATP8A2* | 0.903 |
|  | ENSG00000272047 | 404672 | *GTF2H5* | 0.903 |
|  | ENSG00000153993 | 223117 | *SEMA3D* | 0.9029 |
|  | ENSG00000165288 | 254065 | *BRWD3* | 0.9029 |
|  | ENSG00000124356 | 10617 | *STAMBP* | 0.9027 |
|  | ENSG00000185070 | 23768 100506718 | *FLRT2* | 0.9026 |
|  | ENSG00000083099 | 57226 | *LYRM2* | 0.9026 |
|  | ENSG00000132275 | 23378 | *RRP8* | 0.9025 |
|  | ENSG00000154760 | 146857 | *SLFN13* | 0.9025 |
|  | ENSG00000150893 | 341640 | *FREM2* | 0.9024 |
|  | ENSG00000163285 | 2565 | *GABRG1* | 0.9024 |
|  | ENSG00000112208 | 9532 | *BAG2* | 0.9024 |
|  | ENSG00000128833 | 55930 | *MYO5C* | 0.9023 |
|  | ENSG00000154124 | 90268 | *FAM105B* | 0.9023 |
|  | ENSG00000120733 | 51780 | *KDM3B* | 0.9023 |
|  | ENSG00000187537 | 641455 | *POTEM* | 0.9023 |
|  | ENSG00000168807 | 6645 | *SNTB2* | 0.9022 |
|  | ENSG00000102189 | 8411 | *EEA1* | 0.9022 |
|  | ENSG00000101343 | 51340 | *CRNKL1* | 0.9022 |
|  | ENSG00000222036 | 404785 | *POTEG* | 0.9022 |
|  | ENSG00000260873 | 6645 | *SNTB2* | 0.9022 |
|  | ENSG00000100596 | 9517 | *SPTLC2* | 0.9021 |
|  | ENSG00000141252 | 55275 | *VPS53* | 0.9021 |
|  | ENSG00000187268 | 171484 | *FAM9C* | 0.902 |
|  | ENSG00000117597 | 27042 | *DIEXF* | 0.902 |
|  | ENSG00000060982 | 586 | *BCAT1* | 0.9019 |
|  | ENSG00000133454 | 84700 | *MYO18B* | 0.9019 |
|  | ENSG00000101558 | 9218 | *VAPA* | 0.9019 |
|  | ENSG00000146166 | 51557 | *LGSN* | 0.9019 |
|  | ENSG00000112541 | 10846 | *PDE10A* | 0.9018 |
|  | ENSG00000125810 | 22918 | *CD93* | 0.9017 |
|  | ENSG00000141431 | 80816 | *ASXL3* | 0.9017 |
|  | ENSG00000140526 | 11057 | *ABHD2* | 0.9017 |
|  | ENSG00000118496 | 84085 | *FBXO30* | 0.9016 |
|  | ENSG00000129422 | 57509 | *MTUS1* | 0.9016 |
|  | ENSG00000131711 | 4131 | *MAP1B* | 0.9016 |
|  | ENSG00000172888 | 285268 | *ZNF621* | 0.9016 |
|  | ENSG00000198380 | 2673 | *GFPT1* | 0.9016 |
|  | ENSG00000051825 | 10198 | *MPHOSPH9* | 0.9015 |
|  | ENSG00000042980 | 10863 | *ADAM28* | 0.9015 |
|  | ENSG00000174437 | 488 | *ATP2A2* | 0.9014 |
|  | ENSG00000113389 | 4883 | *NPR3* | 0.9013 |
|  | ENSG00000151834 | 2555 | *GABRA2* | 0.9013 |
|  | ENSG00000167378 | 126298 | *IRGQ* | 0.9013 |
|  | ENSG00000112394 | 117247 | *SLC16A10* | 0.9012 |
|  | ENSG00000147862 | 4781 | *NFIB* | 0.9012 |
|  | ENSG00000165959 | 79789 | *CLMN* | 0.901 |
|  | ENSG00000241978 | 11217 445815 | *AKAP2* | 0.9008 |
|  | ENSG00000157654 | 11217 445815 | *PALM2-AKAP2* | 0.9008 |
|  | ENSG00000163430 | 11167 | *FSTL1* | 0.9007 |
|  | ENSG00000119314 | 9991 | *PTBP3* | 0.9005 |
|  | ENSG00000134250 | 4853 | *NOTCH2* | 0.9005 |
|  | ENSG00000111859 | 4739 | *NEDD9* | 0.9005 |
|  | ENSG00000264983 | 388677 | *NA* | 0.9005 |
|  | ENSG00000176049 | 9832 | *JAKMIP2* | 0.9004 |
|  | ENSG00000136811 | 4957 | *ODF2* | 0.9004 |
|  | ENSG00000163635 | 6314 | *ATXN7* | 0.9002 |
|  | ENSG00000165699 | 7248 | *TSC1* | 0.9002 |
|  | ENSG00000134874 | 22873 | *DZIP1* | 0.9001 |
|  | ENSG00000129204 | 9098 | *USP6* | 0.9 |
| ***mol-miR-168a*** | ENSG00000155966 | 2334 | *AFF2* | 0.9241 |
|  | ENSG00000269754 | 2334 | *AFF2* | 0.9241 |
|  | ENSG00000153721 | 154043 | *CNKSR3* | 0.9241 |
|  | ENSG00000173230 | 2804 | *GOLGB1* | 0.9241 |
|  | ENSG00000126070 | 192669 | *AGO3* | 0.924 |
|  | ENSG00000104290 | 7976 | *FZD3* | 0.924 |
|  | ENSG00000078177 | 55728 | *N4BP2* | 0.924 |
|  | ENSG00000163590 | 151742 | *PPM1L* | 0.924 |
|  | ENSG00000260873 | 6645 | *SNTB2* | 0.924 |
|  | ENSG00000168807 | 6645 | *SNTB2* | 0.924 |
|  | ENSG00000182389 | 785 | *CACNB4* | 0.9239 |
|  | ENSG00000174840 | 201626 | *PDE12* | 0.9239 |
|  | ENSG00000166450 | 283659 | *PRTG* | 0.9239 |
|  | ENSG00000165186 | 139411 | *PTCHD1* | 0.9239 |
|  | ENSG00000145725 | 23262 | *PPIP5K2* | 0.9238 |
|  | ENSG00000009413 | 5980 | *REV3L* | 0.9238 |
|  | ENSG00000134982 | 324 | *APC* | 0.9237 |
|  | ENSG00000172795 | 167227 | *DCP2* | 0.9237 |
|  | ENSG00000178568 | 2066 | *ERBB4* | 0.9237 |
|  | ENSG00000135968 | 9648 | *GCC2* | 0.9237 |
|  | ENSG00000134352 | 3572 | *IL6ST* | 0.9237 |
|  | ENSG00000135297 | 25821 | *MTO1* | 0.9237 |
|  | ENSG00000162409 | 5563 | *PRKAA2* | 0.9237 |
|  | ENSG00000137502 | 27314 | *RAB30* | 0.9237 |
|  | ENSG00000173208 | 225 | *ABCD2* | 0.9236 |
|  | ENSG00000139163 | 55500 | *ETNK1* | 0.9236 |
|  | ENSG00000109452 | 8821 | *INPP4B* | 0.9236 |
|  | ENSG00000119900 | 79627 | *OGFRL1* | 0.9236 |
|  | ENSG00000164398 | 23305 | *ACSL6* | 0.9234 |
|  | ENSG00000112773 | 55603 | *FAM46A* | 0.9232 |
|  | ENSG00000152495 | 814 | *CAMK4* | 0.9231 |
|  | ENSG00000148019 | 84131 | *CEP78* | 0.9231 |
|  | ENSG00000184156 | 3786 | *KCNQ3* | 0.9231 |
|  | ENSG00000055609 | 58508 | *KMT2C* | 0.9231 |
|  | ENSG00000269670 | 347404 | *LANCL3* | 0.9231 |
|  | ENSG00000147036 | 347404 | *LANCL3* | 0.9231 |
|  | ENSG00000076770 | 55796 | *MBNL3* | 0.9231 |
|  | ENSG00000196865 | 374354 | *NHLRC2* | 0.9231 |
|  | ENSG00000140538 | 4916 | *NTRK3* | 0.9231 |
|  | ENSG00000119547 | 9480 | *ONECUT2* | 0.9231 |
|  | ENSG00000171016 | 26108 | *PYGO1* | 0.9231 |
|  | ENSG00000169213 | 5865 | *RAB3B* | 0.9231 |
|  | ENSG00000198743 | 6526 | *SLC5A3* | 0.9231 |
|  | ENSG00000010244 | 7756 | *ZNF207* | 0.9231 |
|  | ENSG00000164684 | 619279 | *ZNF704* | 0.9231 |
|  | ENSG00000163297 | 118429 | *ANTXR2* | 0.923 |
|  | ENSG00000124406 | 10396 | *ATP8A1* | 0.923 |
|  | ENSG00000036530 | 10858 | *CYP46A1* | 0.923 |
|  | ENSG00000145864 | 2561 | *GABRB2* | 0.923 |
|  | ENSG00000145451 | 8001 | *GLRA3* | 0.923 |
|  | ENSG00000123213 | 57486 | *NLN* | 0.923 |
|  | ENSG00000184005 | 256435 | *ST6GALNAC3* | 0.923 |
|  | ENSG00000145779 | 25816 | *TNFAIP8* | 0.923 |
|  | ENSG00000108582 | 1362 | *CPD* | 0.9229 |
|  | ENSG00000133739 | 85444 | *LRRCC1* | 0.9229 |
|  | ENSG00000147862 | 4781 | *NFIB* | 0.9229 |
|  | ENSG00000050628 | 5733 | *PTGER3* | 0.9229 |
|  | ENSG00000111490 | 23329 | *TBC1D30* | 0.9229 |
|  | ENSG00000152078 | 148534 | *TMEM56* | 0.9229 |
|  | ENSG00000136861 | 55755 | *CDK5RAP2* | 0.9228 |
|  | ENSG00000083799 | 1540 | *CYLD* | 0.9228 |
|  | ENSG00000122741 | 79269 | *DCAF10* | 0.9228 |
|  | ENSG00000164031 | 79982 | *DNAJB14* | 0.9228 |
|  | ENSG00000186687 | 90624 | *LYRM7* | 0.9228 |
|  | ENSG00000115421 | 64895 | *PAPOLG* | 0.9228 |
|  | ENSG00000165434 | 283209 | *PGM2L1* | 0.9228 |
|  | ENSG00000153147 | 8467 | *SMARCA5* | 0.9228 |
|  | ENSG00000154429 | 126731 | *CCSAP* | 0.9227 |
|  | ENSG00000143797 | 129642 | *MBOAT2* | 0.9227 |
|  | ENSG00000005889 | 7543 | *ZFX* | 0.9227 |
|  | ENSG00000070018 | 4040 | *LRP6* | 0.9226 |
|  | ENSG00000151414 | 140609 | *NEK7* | 0.9226 |
|  | ENSG00000111371 | 81539 | *SLC38A1* | 0.9226 |
|  | ENSG00000051382 | 5291 | *PIK3CB* | 0.9225 |
|  | ENSG00000093000 | 10762 | *NUP50* | 0.9224 |
|  | ENSG00000144228 | 339745 | *SPOPL* | 0.9224 |
|  | ENSG00000046653 | 2824 | *GPM6B* | 0.9223 |
|  | ENSG00000183775 | 57528 | *KCTD16* | 0.9222 |
|  | ENSG00000175087 | 149420 | *PDIK1L* | 0.9222 |
|  | ENSG00000110344 | 9354 | *UBE4A* | 0.9221 |
|  | ENSG00000268110 | 9354 | *UBE4A* | 0.9221 |
|  | ENSG00000138138 | 84896 | *ATAD1* | 0.922 |
|  | ENSG00000173068 | 54796 | *BNC2* | 0.922 |
|  | ENSG00000206052 | 220164 | *DOK6* | 0.922 |
|  | ENSG00000151914 | 667 | *DST* | 0.922 |
|  | ENSG00000172461 | 10690 | *FUT9* | 0.922 |
|  | ENSG00000205726 | 6453 | *ITSN1* | 0.922 |
|  | ENSG00000102908 | 10725 | *NFAT5* | 0.922 |
|  | ENSG00000143190 | 5451 | *POU2F1* | 0.922 |
|  | ENSG00000079950 | 8417 | *STX7* | 0.922 |
|  | ENSG00000082438 | 22837 | *COBLL1* | 0.9219 |
|  | ENSG00000118260 | 1385 | *CREB1* | 0.9219 |
|  | ENSG00000178662 | 80034 | *CSRNP3* | 0.9219 |
|  | ENSG00000189350 | 165186 | *FAM179A* | 0.9219 |
|  | ENSG00000182263 | 55137 | *FIGN* | 0.9219 |
|  | ENSG00000272047 | 404672 | *GTF2H5* | 0.9219 |
|  | ENSG00000112379 | 57221 | *KIAA1244* | 0.9219 |
|  | ENSG00000123684 | 9926 | *LPGAT1* | 0.9219 |
|  | ENSG00000114857 | 4820 | *NKTR* | 0.9219 |
|  | ENSG00000154678 | 5137 | *PDE1C* | 0.9219 |
|  | ENSG00000112531 | 9444 | *QKI* | 0.9219 |
|  | ENSG00000144642 | 27303 | *RBMS3* | 0.9219 |
|  | ENSG00000080298 | 5991 | *RFX3* | 0.9219 |
|  | ENSG00000187079 | 7003 | *TEAD1* | 0.9219 |
|  | ENSG00000164180 | 153396 | *TMEM161B* | 0.9219 |
|  | ENSG00000128872 | 29767 | *TMOD2* | 0.9219 |
|  | ENSG00000151718 | 53842 80014 | *WWC2* | 0.9219 |
|  | ENSG00000188177 | 376940 | *ZC3H6* | 0.9219 |
|  | ENSG00000106261 | 7586 | *ZKSCAN1* | 0.9219 |
|  | ENSG00000110888 | 65981 | *CAPRIN2* | 0.9218 |
|  | ENSG00000118922 | 11278 | *KLF12* | 0.9218 |
|  | ENSG00000121454 | 89884 | *LHX4* | 0.9218 |
|  | ENSG00000204406 | 55777 | *MBD5* | 0.9218 |
|  | ENSG00000173542 | 92597 | *MOB1B* | 0.9218 |
|  | ENSG00000154654 | 4685 | *NCAM2* | 0.9218 |
|  | ENSG00000169760 | 22871 | *NLGN1* | 0.9218 |
|  | ENSG00000113494 | 5618 | *PRLR* | 0.9218 |
|  | ENSG00000171862 | 5728 | *PTEN* | 0.9218 |
|  | ENSG00000127328 | 117177 | *RAB3IP* | 0.9218 |
|  | ENSG00000138670 | 153020 | *RASGEF1B* | 0.9218 |
|  | ENSG00000182010 | 219790 | *RTKN2* | 0.9218 |
|  | ENSG00000179542 | 139065 | *SLITRK4* | 0.9218 |
|  | ENSG00000177565 | 79718 | *TBL1XR1* | 0.9218 |
|  | ENSG00000181634 | 9966 | *TNFSF15* | 0.9218 |
|  | ENSG00000182463 | 128553 | *TSHZ2* | 0.9218 |
|  | ENSG00000181626 | 342850 | *ANKRD62* | 0.9217 |
|  | ENSG00000203668 | 1122 | *CHML* | 0.9217 |
|  | ENSG00000169018 | 10116 | *FEM1B* | 0.9217 |
|  | ENSG00000178695 | 115207 | *KCTD12* | 0.9217 |
|  | ENSG00000163808 | 56992 | *KIF15* | 0.9217 |
|  | ENSG00000117533 | 8674 | *VAMP4* | 0.9217 |
|  | ENSG00000143324 | 9213 | *XPR1* | 0.9217 |
|  | ENSG00000138443 | 10152 | *ABI2* | 0.9216 |
|  | ENSG00000137145 | 55667 | *DENND4C* | 0.9216 |
|  | ENSG00000180628 | 84333 | *PCGF5* | 0.9216 |
|  | ENSG00000139151 | 89869 | *PLCZ1* | 0.9216 |
|  | ENSG00000103769 | 8766 | *RAB11A* | 0.9216 |
|  | ENSG00000166257 | 55800 | *SCN3B* | 0.9216 |
|  | ENSG00000097033 | 51100 | *SH3GLB1* | 0.9216 |
|  | ENSG00000072274 | 7037 | *TFRC* | 0.9216 |
|  | ENSG00000170185 | 84640 | *USP38* | 0.9216 |
|  | ENSG00000211448 | 1734 | *DIO2* | 0.9215 |
|  | ENSG00000011201 | 3730 | *KAL1* | 0.9215 |
|  | ENSG00000203727 | 389432 | *SAMD5* | 0.9215 |
|  | ENSG00000112339 | 10767 | *HBS1L* | 0.9214 |
|  | ENSG00000154001 | 5529 | *PPP2R5E* | 0.9214 |
|  | ENSG00000148158 | 401548 | *SNX30* | 0.9214 |
|  | ENSG00000151458 | 57182 | *ANKRD50* | 0.9213 |
|  | ENSG00000154727 | 2551 | *GABPA* | 0.9213 |
|  | ENSG00000164134 | 80155 | *NAA15* | 0.9213 |
|  | ENSG00000102471 | 54602 | *NDFIP2* | 0.9213 |
|  | ENSG00000132388 | 7326 | *UBE2G1* | 0.9213 |
|  | ENSG00000154263 | 10349 | *ABCA10* | 0.9212 |
|  | ENSG00000118985 | 22936 | *ELL2* | 0.9212 |
|  | ENSG00000127507 | 30817 | *EMR2* | 0.9212 |
|  | ENSG00000153395 | 79888 | *LPCAT1* | 0.9212 |
|  | ENSG00000135341 | 6885 | *MAP3K7* | 0.9212 |
|  | ENSG00000169116 | 25849 | *PARM1* | 0.9212 |
|  | ENSG00000146587 | 57786 389458 100533952 | *RBAK* | 0.9212 |
|  | ENSG00000113810 | 10051 | *SMC4* | 0.9212 |
|  | ENSG00000088756 | 79822 | *ARHGAP28* | 0.9211 |
|  | ENSG00000205339 | 10527 | *IPO7* | 0.9211 |
|  | ENSG00000198914 | 5455 | *POU3F3* | 0.9211 |
|  | ENSG00000196458 | 1E+08 | *ZNF605* | 0.9211 |
|  | ENSG00000151917 | 221336 | *BEND6* | 0.921 |
|  | ENSG00000181264 | 219902 | *TMEM136* | 0.921 |
|  | ENSG00000247746 | 158880 | *USP51* | 0.921 |
|  | ENSG00000143297 | 83416 | *FCRL5* | 0.9209 |
|  | ENSG00000185231 | 4158 | *MC2R* | 0.9209 |
|  | ENSG00000171735 | 23261 | *CAMTA1* | 0.9208 |
|  | ENSG00000164659 | 222223 | *KIAA1324L* | 0.9208 |
|  | ENSG00000157330 | 93190 | *C1orf158* | 0.9207 |
|  | ENSG00000137960 | 54810 | *GIPC2* | 0.9206 |
|  | ENSG00000139915 | 161357 | *MDGA2* | 0.9206 |
|  | ENSG00000139324 | 160418 | *TMTC3* | 0.9206 |
|  | ENSG00000163507 | 57650 | *KIAA1524* | 0.9205 |
|  | ENSG00000151148 | 89910 | *UBE3B* | 0.9205 |
|  | ENSG00000145703 | 10788 | *IQGAP2* | 0.9203 |
|  | ENSG00000153207 | 25909 | *AHCTF1* | 0.9197 |
|  | ENSG00000140386 | 49855 | *SCAPER* | 0.9197 |
|  | ENSG00000105810 | 1021 | *CDK6* | 0.9193 |
|  | ENSG00000055332 | 5610 | *EIF2AK2* | 0.9193 |
|  | ENSG00000138185 | 953 | *ENTPD1* | 0.9193 |
|  | ENSG00000144791 | 8994 | *LIMD1* | 0.9193 |
|  | ENSG00000128585 | 4289 | *MKLN1* | 0.9193 |
|  | ENSG00000082175 | 5241 | *PGR* | 0.9193 |
|  | ENSG00000146373 | 154214 | *RNF217* | 0.9193 |
|  | ENSG00000112394 | 117247 | *SLC16A10* | 0.9193 |
|  | ENSG00000143970 | 55252 | *ASXL2* | 0.9192 |
|  | ENSG00000060982 | 586 | *BCAT1* | 0.9192 |
|  | ENSG00000175161 | 253559 | *CADM2* | 0.9192 |
|  | ENSG00000066294 | 8832 | *CD84* | 0.9192 |
|  | ENSG00000150394 | 1006 | *CDH8* | 0.9192 |
|  | ENSG00000153933 | 8526 | *DGKE* | 0.9192 |
|  | ENSG00000145907 | 10146 | *G3BP1* | 0.9192 |
|  | ENSG00000168172 | 84376 | *HOOK3* | 0.9192 |
|  | ENSG00000122778 | 57670 | *KIAA1549* | 0.9192 |
|  | ENSG00000178425 | 221294 | *NT5DC1* | 0.9192 |
|  | ENSG00000243444 | 114299 | *PALM2* | 0.9192 |
|  | ENSG00000078142 | 5289 | *PIK3C3* | 0.9192 |
|  | ENSG00000152104 | 5784 | *PTPN14* | 0.9192 |
|  | ENSG00000088179 | 5775 | *PTPN4* | 0.9192 |
|  | ENSG00000076053 | 10179 | *RBM7* | 0.9192 |
|  | ENSG00000177570 | 401474 | *SAMD12* | 0.9192 |
|  | ENSG00000110436 | 6506 | *SLC1A2* | 0.9192 |
|  | ENSG00000160551 | 57551 | *TAOK1* | 0.9192 |
|  | ENSG00000116747 | 6738 | *TROVE2* | 0.9192 |
|  | ENSG00000163960 | 26043 | *UBXN7* | 0.9192 |
|  | ENSG00000205189 | 65986 | *ZBTB10* | 0.9192 |
|  | ENSG00000197779 | 347344 | *ZNF81* | 0.9192 |
|  | ENSG00000269594 | 347344 | *ZNF81* | 0.9192 |
|  | ENSG00000169083 | 367 | *AR* | 0.9191 |
|  | ENSG00000112182 | 60468 | *BACH2* | 0.9191 |
|  | ENSG00000204116 | 53344 | *CHIC1* | 0.9191 |
|  | ENSG00000175390 | 8665 | *EIF3F* | 0.9191 |
|  | ENSG00000163412 | 317649 | *EIF4E3* | 0.9191 |
|  | ENSG00000152409 | 133746 | *JMY* | 0.9191 |
|  | ENSG00000050030 | 340533 | *KIAA2022* | 0.9191 |
|  | ENSG00000188906 | 120892 | *LRRK2* | 0.9191 |
|  | ENSG00000071073 | 11320 | *MGAT4A* | 0.9191 |
|  | ENSG00000153029 | 3140 | *MR1* | 0.9191 |
|  | ENSG00000113971 | 27031 | *NPHP3* | 0.9191 |
|  | ENSG00000108256 | 57532 | *NUFIP2* | 0.9191 |
|  | ENSG00000177425 | 5074 | *PAWR* | 0.9191 |
|  | ENSG00000112419 | 9749 | *PHACTR2* | 0.9191 |
|  | ENSG00000118482 | 23469 | *PHF3* | 0.9191 |
|  | ENSG00000221866 | 91584 | *PLXNA4* | 0.9191 |
|  | ENSG00000046889 | 80243 | *PREX2* | 0.9191 |
|  | ENSG00000143248 | 8490 | *RGS5* | 0.9191 |
|  | ENSG00000112246 | 6492 | *SIM1* | 0.9191 |
|  | ENSG00000117500 | 50999 | *TMED5* | 0.9191 |
|  | ENSG00000083312 | 3842 | *TNPO1* | 0.9191 |
|  | ENSG00000182168 | 8633 | *UNC5C* | 0.9191 |
|  | ENSG00000164663 | 25862 | *USP49* | 0.9191 |
|  | ENSG00000206538 | 389136 | *VGLL3* | 0.9191 |
|  | ENSG00000172466 | 7572 | *ZNF24* | 0.9191 |
|  | ENSG00000156804 | 114907 | *FBXO32* | 0.919 |
|  | ENSG00000248905 | 342184 | *FMN1* | 0.919 |
|  | ENSG00000118689 | 2309 | *FOXO3* | 0.919 |
|  | ENSG00000106701 | 83856 | *FSD1L* | 0.919 |
|  | ENSG00000156052 | 2776 | *GNAQ* | 0.919 |
|  | ENSG00000153936 | 9653 | *HS2ST1* | 0.919 |
|  | ENSG00000172260 | 257194 | *NEGR1* | 0.919 |
|  | ENSG00000113448 | 5144 | *PDE4D* | 0.919 |
|  | ENSG00000146247 | 55023 | *PHIP* | 0.919 |
|  | ENSG00000121879 | 5290 | *PIK3CA* | 0.919 |
|  | ENSG00000152061 | 9910 | *RABGAP1L* | 0.919 |
|  | ENSG00000162521 | 5928 | *RBBP4* | 0.919 |
|  | ENSG00000177189 | 6197 | *RPS6KA3* | 0.919 |
|  | ENSG00000080493 | 8671 | *SLC4A4* | 0.919 |
|  | ENSG00000189362 | 1E+08 | *TMEM194B* | 0.919 |
|  | ENSG00000141655 | 8792 | *TNFRSF11A* | 0.919 |
|  | ENSG00000132953 | 64328 | *XPO4* | 0.919 |
|  | ENSG00000185728 | 253943 | *YTHDF3* | 0.919 |
|  | ENSG00000121406 | 256051 | *ZNF549* | 0.919 |
|  | ENSG00000139083 | 2120 | *ETV6* | 0.9189 |
|  | ENSG00000165669 | 63877 | *FAM204A* | 0.9189 |
|  | ENSG00000116199 | 9917 | *FAM20B* | 0.9189 |
|  | ENSG00000189308 | 132660 | *LIN54* | 0.9189 |
|  | ENSG00000145416 | 55016 | *MARCH1* | 0.9189 |
|  | ENSG00000218497 | 7637 100996464 | *NA* | 0.9189 |
|  | ENSG00000104320 | 4683 | *NBN* | 0.9189 |
|  | ENSG00000007372 | 5080 | *PAX6* | 0.9189 |
|  | ENSG00000163637 | 166336 | *PRICKLE2* | 0.9189 |
|  | ENSG00000113522 | 10111 | *RAD50* | 0.9189 |
|  | ENSG00000181704 | 286451 | *YIPF6* | 0.9189 |
|  | ENSG00000198040 | 7637 100996464 | *ZNF84* | 0.9189 |
|  | ENSG00000163697 | 323 | *APBB2* | 0.9188 |
|  | ENSG00000171885 | 361 | *AQP4* | 0.9188 |
|  | ENSG00000143079 | 55917 | *CTTNBP2NL* | 0.9188 |
|  | ENSG00000165323 | 120114 | *FAT3* | 0.9188 |
|  | ENSG00000263334 | 3092 | *HIP1* | 0.9188 |
|  | ENSG00000127946 | 3092 | *HIP1* | 0.9188 |
|  | ENSG00000198000 | 55035 | *NOL8* | 0.9188 |
|  | ENSG00000166501 | 5579 | *PRKCB* | 0.9188 |
|  | ENSG00000138434 | 6744 | *SSFA2* | 0.9188 |
|  | ENSG00000166415 | 256764 | *WDR72* | 0.9188 |
|  | ENSG00000149311 | 472 | *ATM* | 0.9187 |
|  | ENSG00000165813 | 55088 | *C10orf118* | 0.9187 |
|  | ENSG00000180881 | 84698 | *CAPS2* | 0.9187 |
|  | ENSG00000115042 | 51011 | *FAHD2A* | 0.9187 |
|  | ENSG00000138286 | 317662 | *FAM149B1* | 0.9187 |
|  | ENSG00000116977 | 3964 | *LGALS8* | 0.9187 |
|  | ENSG00000013375 | 5238 | *PGM3* | 0.9187 |
|  | ENSG00000131242 | 84440 | *RAB11FIP4* | 0.9187 |
|  | ENSG00000099246 | 22931 | *RAB18* | 0.9187 |
|  | ENSG00000172007 | 83452 | *RAB33B* | 0.9187 |
|  | ENSG00000137266 | 63027 | *SLC22A23* | 0.9187 |
|  | ENSG00000132122 | 54558 | *SPATA6* | 0.9187 |
|  | ENSG00000169895 | 94056 | *SYAP1* | 0.9187 |
|  | ENSG00000187555 | 7874 | *USP7* | 0.9187 |
|  | ENSG00000114251 | 7474 | *WNT5A* | 0.9187 |
|  | ENSG00000172493 | 4299 | *AFF1* | 0.9186 |
|  | ENSG00000072364 | 27125 | *AFF4* | 0.9186 |
|  | ENSG00000166845 | 162681 | *C18orf54* | 0.9186 |
|  | ENSG00000102531 | 22862 | *FNDC3A* | 0.9186 |
|  | ENSG00000106031 | 3209 | *HOXA13* | 0.9186 |
|  | ENSG00000145332 | 57563 | *KLHL8* | 0.9186 |
|  | ENSG00000167005 | 11051 | *NUDT21* | 0.9186 |
|  | ENSG00000139144 | 5288 | *PIK3C2G* | 0.9186 |
|  | ENSG00000164244 | 133619 | *PRRC1* | 0.9186 |
|  | ENSG00000070950 | 56852 | *RAD18* | 0.9186 |
|  | ENSG00000071242 | 6196 | *RPS6KA2* | 0.9186 |
|  | ENSG00000120784 | 22835 | *ZFP30* | 0.9186 |
|  | ENSG00000197343 | 79027 | *ZNF655* | 0.9186 |
|  | ENSG00000157500 | 26060 | *APPL1* | 0.9185 |
|  | ENSG00000188107 | 346007 | *EYS* | 0.9185 |
|  | ENSG00000162981 | 151354 | *FAM84A* | 0.9185 |
|  | ENSG00000168672 | 157638 | *FAM84B* | 0.9185 |
|  | ENSG00000100522 | 64841 | *GNPNAT1* | 0.9185 |
|  | ENSG00000187772 | 389421 | *LIN28B* | 0.9185 |
|  | ENSG00000144959 | 57552 | *NCEH1* | 0.9185 |
|  | ENSG00000078043 | 9063 | *PIAS2* | 0.9185 |
|  | ENSG00000112739 | 8899 | *PRPF4B* | 0.9185 |
|  | ENSG00000139746 | 64062 | *RBM26* | 0.9185 |
|  | ENSG00000134278 | 56907 | *SPIRE1* | 0.9185 |
|  | ENSG00000172765 | 23023 | *TMCC1* | 0.9185 |
|  | ENSG00000101019 | 55245 | *UQCC* | 0.9185 |
|  | ENSG00000116985 | 656 | *BMP8B* | 0.9184 |
|  | ENSG00000258289 | 91612 | *CHURC1* | 0.9184 |
|  | ENSG00000060749 | 79832 | *QSER1* | 0.9184 |
|  | ENSG00000060709 | 23504 | *RIMBP2* | 0.9184 |
|  | ENSG00000156463 | 153769 | *SH3RF2* | 0.9184 |
|  | ENSG00000112343 | 10475 | *TRIM38* | 0.9184 |
|  | ENSG00000142065 | 57677 | *ZFP14* | 0.9184 |
|  | ENSG00000235931 | NA | *C10orf40* | 0.9183 |
|  | ENSG00000198087 | 23607 | *CD2AP* | 0.9183 |
|  | ENSG00000178573 | 4094 | *MAF* | 0.9183 |
|  | ENSG00000157890 | 84465 | *MEGF11* | 0.9183 |
|  | ENSG00000165494 | 51585 | *PCF11* | 0.9183 |
|  | ENSG00000103740 | 23205 | *ACSBG1* | 0.9182 |
|  | ENSG00000198668 | 801 805 808 | *CALM1* | 0.9182 |
|  | ENSG00000023318 | 23071 | *ERP44* | 0.9182 |
|  | ENSG00000162636 | 284611 | *FAM102B* | 0.9182 |
|  | ENSG00000106070 | 2887 | *GRB10* | 0.9182 |
|  | ENSG00000172901 | 206338 | *NA* | 0.9182 |
|  | ENSG00000153561 | 64795 | *RMND5A* | 0.9182 |
|  | ENSG00000085491 | 29957 | *SLC25A24* | 0.9182 |
|  | ENSG00000144747 | 7110 | *TMF1* | 0.9182 |
|  | ENSG00000169756 | 3987 | *LIMS1* | 0.9181 |
|  | ENSG00000042429 | 9440 | *MED17* | 0.9181 |
|  | ENSG00000159216 | 861 100506403 | *RUNX1* | 0.9181 |
|  | ENSG00000127526 | 79939 | *SLC35E1* | 0.9181 |
|  | ENSG00000186017 | 84924 | *ZNF566* | 0.9181 |
|  | ENSG00000178538 | 767 | *CA8* | 0.918 |
|  | ENSG00000107249 | 169792 | *GLIS3* | 0.918 |
|  | ENSG00000116001 | 7072 | *TIA1* | 0.918 |
|  | ENSG00000122042 | 5412 | *UBL3* | 0.918 |
|  | ENSG00000164951 | 54704 | *PDP1* | 0.9179 |
|  | ENSG00000106772 | 158471 | *PRUNE2* | 0.9179 |
|  | ENSG00000106686 | 55064 | *SPATA6L* | 0.9179 |
|  | ENSG00000095209 | 55151 | *TMEM38B* | 0.9179 |
|  | ENSG00000110090 | 1374 | *CPT1A* | 0.9178 |
|  | ENSG00000177150 | 125228 | *FAM210A* | 0.9178 |
|  | ENSG00000164100 | 9348 | *NDST3* | 0.9178 |
|  | ENSG00000188725 | 643155 | *SMIM15* | 0.9178 |
|  | ENSG00000170088 | 201931 | *TMEM192* | 0.9178 |
|  | ENSG00000165832 | 142940 | *TRUB1* | 0.9178 |
|  | ENSG00000134371 | 79577 | *CDC73* | 0.9177 |
|  | ENSG00000196227 | 63939 | *FAM217B* | 0.9177 |
|  | ENSG00000189403 | 3146 | *HMGB1* | 0.9177 |
|  | ENSG00000136111 | 9882 | *TBC1D4* | 0.9177 |
|  | ENSG00000081026 | 260425 | *MAGI3* | 0.9176 |
|  | ENSG00000117090 | 6504 | *SLAMF1* | 0.9176 |
|  | ENSG00000147679 | 84294 | *UTP23* | 0.9176 |
|  | ENSG00000113532 | 7903 | *ST8SIA4* | 0.9175 |
|  | ENSG00000163092 | 129446 | *XIRP2* | 0.9175 |
|  | ENSG00000256660 | 387837 | *CLEC12B* | 0.9174 |
|  | ENSG00000158290 | 8450 | *CUL4B* | 0.9174 |
|  | ENSG00000163518 | 83417 | *FCRL4* | 0.9174 |
|  | ENSG00000255661 | NA | *NA* | 0.9174 |
|  | ENSG00000120278 | 57480 | *PLEKHG1* | 0.9174 |
|  | ENSG00000244694 | 442213 | *PTCHD4* | 0.9174 |
|  | ENSG00000170759 | 3799 | *KIF5B* | 0.9173 |
|  | ENSG00000073711 | 5523 | *PPP2R3A* | 0.9173 |
|  | ENSG00000183161 | 2188 | *FANCF* | 0.9172 |
|  | ENSG00000164830 | 55074 | *OXR1* | 0.9172 |
|  | ENSG00000102104 | 6247 | *RS1* | 0.9172 |
|  | ENSG00000181381 | 91351 | *DDX60L* | 0.9171 |
|  | ENSG00000164308 | 64167 | *ERAP2* | 0.917 |
|  | ENSG00000177707 | 25945 | *PVRL3* | 0.917 |
|  | ENSG00000152256 | 5163 | *PDK1* | 0.9169 |
|  | ENSG00000197851 | 401388 | *C7orf76* | 0.9168 |
|  | ENSG00000256043 | 1519 | *CTSO* | 0.9168 |
|  | ENSG00000263238 | 1519 | *CTSO* | 0.9168 |
|  | ENSG00000081803 | 93664 | *CADPS2* | 0.9167 |
|  | ENSG00000268943 | 1193 | *CLIC2* | 0.9167 |
|  | ENSG00000155962 | 1193 | *CLIC2* | 0.9167 |
|  | ENSG00000248874 | NA | *C5orf17* | 0.9166 |
|  | ENSG00000156284 | 9073 | *CLDN8* | 0.9166 |
|  | ENSG00000138379 | 2660 | *MSTN* | 0.9166 |
|  | ENSG00000104177 | 50804 | *MYEF2* | 0.9166 |
|  | ENSG00000079134 | 9984 | *THOC1* | 0.9165 |
|  | ENSG00000012983 | 11183 | *MAP4K5* | 0.9163 |
|  | ENSG00000177272 | 3738 | *KCNA3* | 0.916 |
|  | ENSG00000139323 | 282809 | *POC1B* | 0.9159 |
|  | ENSG00000062194 | 65056 | *GPBP1* | 0.9158 |
|  | ENSG00000113638 | 23548 | *TTC33* | 0.9158 |
|  | ENSG00000184486 | 5454 | *POU3F2* | 0.9157 |
|  | ENSG00000038427 | 1462 | *VCAN* | 0.915 |
|  | ENSG00000092847 | 26523 | *AGO1* | 0.9119 |
|  | ENSG00000158258 | 64084 | *CLSTN2* | 0.9119 |
|  | ENSG00000077279 | 1641 | *DCX* | 0.9119 |
|  | ENSG00000146267 | 84553 | *FAXC* | 0.9119 |
|  | ENSG00000089916 | 55668 | *GPATCH2L* | 0.9119 |
|  | ENSG00000051620 | 23593 | *HEBP2* | 0.9119 |
|  | ENSG00000263162 | 8924 100653292 | *HERC2* | 0.9119 |
|  | ENSG00000064393 | 28996 | *HIPK2* | 0.9119 |
|  | ENSG00000149948 | 8091 | *HMGA2* | 0.9119 |
|  | ENSG00000114933 | 54891 | *INO80D* | 0.9119 |
|  | ENSG00000156113 | 3778 | *KCNMA1* | 0.9119 |
|  | ENSG00000165185 | 158405 | *KIAA1958* | 0.9119 |
|  | ENSG00000171435 | 283455 | *KSR2* | 0.9119 |
|  | ENSG00000255330 | 9729 387104 | *NA* | 0.9119 |
|  | ENSG00000120137 | 79646 | *PANK3* | 0.9119 |
|  | ENSG00000152932 | 115827 | *RAB3C* | 0.9119 |
|  | ENSG00000146414 | 257218 | *SHPRH* | 0.9119 |
|  | ENSG00000214338 | 9729 387104 | *SOGA3* | 0.9119 |
|  | ENSG00000177511 | 51046 | *ST8SIA3* | 0.9119 |
|  | ENSG00000104343 | 55284 | *UBE2W* | 0.9119 |
|  | ENSG00000118492 | 79747 | *ADGB* | 0.9118 |
|  | ENSG00000165566 | 219287 | *AMER2* | 0.9118 |
|  | ENSG00000183166 | 83698 | *CALN1* | 0.9118 |
|  | ENSG00000198010 | 9228 | *DLGAP2* | 0.9118 |
|  | ENSG00000184949 | 646851 | *FAM227A* | 0.9118 |
|  | ENSG00000140718 | 79068 | *FTO* | 0.9118 |
|  | ENSG00000152749 | 160897 | *GPR180* | 0.9118 |
|  | ENSG00000144366 | 51454 | *GULP1* | 0.9118 |
|  | ENSG00000198700 | 55705 | *IPO9* | 0.9118 |
|  | ENSG00000213949 | 3672 | *ITGA1* | 0.9118 |
|  | ENSG00000186432 | 3840 | *KPNA4* | 0.9118 |
|  | ENSG00000113441 | 4012 | *LNPEP* | 0.9118 |
|  | ENSG00000169967 | 10746 | *MAP3K2* | 0.9118 |
|  | ENSG00000163291 | 152559 | *PAQR3* | 0.9118 |
|  | ENSG00000091844 | 26575 | *RGS17* | 0.9118 |
|  | ENSG00000170624 | 6444 | *SGCD* | 0.9118 |
|  | ENSG00000070214 | 23446 | *SLC44A1* | 0.9118 |
|  | ENSG00000151012 | 23657 | *SLC7A11* | 0.9118 |
|  | ENSG00000185518 | 9899 | *SV2B* | 0.9118 |
|  | ENSG00000196628 | 6925 | *TCF4* | 0.9118 |
|  | ENSG00000114126 | 7029 | *TFDP2* | 0.9118 |
|  | ENSG00000196581 | 55966 | *AJAP1* | 0.9117 |
|  | ENSG00000112309 | 135152 | *B3GAT2* | 0.9117 |
|  | ENSG00000187118 | 152100 | *CMC1* | 0.9117 |
|  | ENSG00000146592 | 9586 401317 | *CREB5* | 0.9117 |
|  | ENSG00000126733 | 117154 | *DACH2* | 0.9117 |
|  | ENSG00000151233 | 283464 | *GXYLT1* | 0.9117 |
|  | ENSG00000167378 | 126298 | *IRGQ* | 0.9117 |
|  | ENSG00000102781 | 84056 | *KATNAL1* | 0.9117 |
|  | ENSG00000118263 | 8609 | *KLF7* | 0.9117 |
|  | ENSG00000114030 | 3836 | *KPNA1* | 0.9117 |
|  | ENSG00000073803 | 9175 | *MAP3K13* | 0.9117 |
|  | ENSG00000162601 | 114803 | *MYSM1* | 0.9117 |
|  | ENSG00000134369 | 89796 | *NAV1* | 0.9117 |
|  | ENSG00000076641 | 55824 | *PAG1* | 0.9117 |
|  | ENSG00000155846 | 133522 | *PPARGC1B* | 0.9117 |
|  | ENSG00000158528 | 55607 | *PPP1R9A* | 0.9117 |
|  | ENSG00000124209 | 57403 | *RAB22A* | 0.9117 |
|  | ENSG00000079102 | 862 | *RUNX1T1* | 0.9117 |
|  | ENSG00000118473 | 84251 | *SGIP1* | 0.9117 |
|  | ENSG00000169905 | 163590 | *TOR1AIP2* | 0.9117 |
|  | ENSG00000180011 | 284273 | *ZADH2* | 0.9117 |
|  | ENSG00000197928 | 342926 | *ZNF677* | 0.9117 |
|  | ENSG00000197302 | 124411 | *ZNF720* | 0.9117 |
|  | ENSG00000136010 | 160428 | *ALDH1L2* | 0.9116 |
|  | ENSG00000196914 | 23365 | *ARHGEF12* | 0.9116 |
|  | ENSG00000165997 | 221079 | *ARL5B* | 0.9116 |
|  | ENSG00000112208 | 9532 | *BAG2* | 0.9116 |
|  | ENSG00000172292 | 253782 | *CERS6* | 0.9116 |
|  | ENSG00000102189 | 8411 | *EEA1* | 0.9116 |
|  | ENSG00000158711 | 2005 | *ELK4* | 0.9116 |
|  | ENSG00000152223 | 57724 | *EPG5* | 0.9116 |
|  | ENSG00000174804 | 8322 | *FZD4* | 0.9116 |
|  | ENSG00000103342 | 2935 | *GSPT1* | 0.9116 |
|  | ENSG00000148634 | 26091 | *HERC4* | 0.9116 |
|  | ENSG00000090376 | 11213 | *IRAK3* | 0.9116 |
|  | ENSG00000173517 | 79834 | *NA* | 0.9116 |
|  | ENSG00000197121 | 80055 | *PGAP1* | 0.9116 |
|  | ENSG00000266198 | 5565 101060511 | *PRKAB2* | 0.9116 |
|  | ENSG00000131791 | 5565 101060511 | *PRKAB2* | 0.9116 |
|  | ENSG00000169435 | 166824 | *RASSF6* | 0.9116 |
|  | ENSG00000048649 | 51773 | *RSF1* | 0.9116 |
|  | ENSG00000145391 | 80854 | *SETD7* | 0.9116 |
|  | ENSG00000196715 | 154807 | *VKORC1L1* | 0.9116 |
|  | ENSG00000109685 | 7468 | *WHSC1* | 0.9116 |
|  | ENSG00000160062 | 653121 | *ZBTB8A* | 0.9116 |
|  | ENSG00000175048 | 79683 | *ZDHHC14* | 0.9116 |
|  | ENSG00000162433 | 205 100507855 | *AK4* | 0.9115 |
|  | ENSG00000078699 | 9139 | *CBFA2T2* | 0.9115 |
|  | ENSG00000170160 | 9720 | *CCDC144A* | 0.9115 |
|  | ENSG00000269240 | 1184 | *CLCN5* | 0.9115 |
|  | ENSG00000171365 | 1184 | *CLCN5* | 0.9115 |
|  | ENSG00000113712 | 1452 | *CSNK1A1* | 0.9115 |
|  | ENSG00000262102 | 92126 | *DSEL* | 0.9115 |
|  | ENSG00000171451 | 92126 | *DSEL* | 0.9115 |
|  | ENSG00000170522 | 79071 | *ELOVL6* | 0.9115 |
|  | ENSG00000147475 | 11160 | *ERLIN2* | 0.9115 |
|  | ENSG00000122591 | 84668 | *FAM126A* | 0.9115 |
|  | ENSG00000198780 | 26049 | *FAM169A* | 0.9115 |
|  | ENSG00000112234 | 26235 | *FBXL4* | 0.9115 |
|  | ENSG00000166225 | 10818 | *FRS2* | 0.9115 |
|  | ENSG00000109458 | 2549 | *GAB1* | 0.9115 |
|  | ENSG00000122641 | 3624 | *INHBA* | 0.9115 |
|  | ENSG00000205730 | 162073 | *ITPRIPL2* | 0.9115 |
|  | ENSG00000115548 | 55818 | *KDM3A* | 0.9115 |
|  | ENSG00000111052 | 8825 | *LIN7A* | 0.9115 |
|  | ENSG00000266302 | NA | *NA* | 0.9115 |
|  | ENSG00000069869 | 4734 | *NEDD4* | 0.9115 |
|  | ENSG00000139910 | 4857 | *NOVA1* | 0.9115 |
|  | ENSG00000170584 | 134492 | *NUDCD2* | 0.9115 |
|  | ENSG00000183943 | 5613 | *PRKX* | 0.9115 |
|  | ENSG00000181467 | 5912 | *RAP2B* | 0.9115 |
|  | ENSG00000146197 | 222663 | *SCUBE3* | 0.9115 |
|  | ENSG00000187231 | 91404 | *SESTD1* | 0.9115 |
|  | ENSG00000169291 | 126669 | *SHE* | 0.9115 |
|  | ENSG00000139514 | 6541 | *SLC7A1* | 0.9115 |
|  | ENSG00000113658 | 4090 | *SMAD5* | 0.9115 |
|  | ENSG00000110693 | 55553 | *SOX6* | 0.9115 |
|  | ENSG00000105967 | 22797 | *TFEC* | 0.9115 |
|  | ENSG00000106799 | 7046 | *TGFBR1* | 0.9115 |
|  | ENSG00000125827 | 56255 | *TMX4* | 0.9115 |
|  | ENSG00000188001 | 285386 101060141 | *TPRG1* | 0.9115 |
|  | ENSG00000154127 | 84959 | *UBASH3B* | 0.9115 |
|  | ENSG00000058056 | 8975 | *USP13* | 0.9115 |
|  | ENSG00000136866 | 7539 | *ZFP37* | 0.9115 |
|  | ENSG00000241978 | 11217 445815 | *AKAP2* | 0.9114 |
|  | ENSG00000132932 | 51761 | *ATP8A2* | 0.9114 |
|  | ENSG00000113742 | 80315 | *CPEB4* | 0.9114 |
|  | ENSG00000066044 | 1994 | *ELAVL1* | 0.9114 |
|  | ENSG00000054965 | 23201 | *FAM168A* | 0.9114 |
|  | ENSG00000128607 | 23008 | *KLHDC10* | 0.9114 |
|  | ENSG00000172578 | 89857 | *KLHL6* | 0.9114 |
|  | ENSG00000183833 | 89876 | *MAATS1* | 0.9114 |
|  | ENSG00000144893 | 116931 | *MED12L* | 0.9114 |
|  | ENSG00000128609 | 4698 | *NDUFA5* | 0.9114 |
|  | ENSG00000157654 | 11217 445815 | *PALM2­AKAP2* | 0.9114 |
|  | ENSG00000183206 | 388468 101060716 | *POTEC* | 0.9114 |
|  | ENSG00000180773 | 120103 | *SLC36A4* | 0.9114 |
|  | ENSG00000148942 | 159963 | *SLC5A12* | 0.9114 |
|  | ENSG00000134532 | 6660 | *SOX5* | 0.9114 |
|  | ENSG00000077147 | 56889 | *TM9SF3* | 0.9114 |
|  | ENSG00000108395 | 4591 | *TRIM37* | 0.9114 |
|  | ENSG00000137941 | 79739 | *TTLL7* | 0.9114 |
|  | ENSG00000103404 | 57478 | *USP31* | 0.9114 |
|  | ENSG00000175073 | 80124 | *VCPIP1* | 0.9114 |
|  | ENSG00000015153 | 10138 | *YAF2* | 0.9114 |
|  | ENSG00000140836 | 463 | *ZFHX3* | 0.9114 |
|  | ENSG00000171714 | 203859 | *ANO5* | 0.9113 |
|  | ENSG00000139971 | 145407 | *C14orf37* | 0.9113 |
|  | ENSG00000151893 | 143384 | *CACUL1* | 0.9113 |
|  | ENSG00000096060 | 2289 | *FKBP5* | 0.9113 |
|  | ENSG00000162769 | 28982 | *FLVCR1* | 0.9113 |
|  | ENSG00000183098 | 10082 | *GPC6* | 0.9113 |
|  | ENSG00000157423 | 54768 100288805101060699 | *HYDIN* | 0.9113 |
|  | ENSG00000006459 | 80853 | *JHDM1D* | 0.9113 |
|  | ENSG00000111262 | 3736 | *KCNA1* | 0.9113 |
|  | ENSG00000124743 | 401265 | *KLHL31* | 0.9113 |
|  | ENSG00000099810 | 4507 | *MTAP* | 0.9113 |
|  | ENSG00000136521 | 4711 | *NDUFB5* | 0.9113 |
|  | ENSG00000123119 | 64168 | *NECAB1* | 0.9113 |
|  | ENSG00000162599 | 4774 | *NFIA* | 0.9113 |
|  | ENSG00000079156 | 114880 | *OSBPL6* | 0.9113 |
|  | ENSG00000185619 | 10336 | *PCGF3* | 0.9113 |
|  | ENSG00000080815 | 5663 | *PSEN1* | 0.9113 |
|  | ENSG00000084453 | 6579 | *SLCO1A2* | 0.9113 |
|  | ENSG00000038295 | 7092 | *TLL1* | 0.9113 |
|  | ENSG00000106771 | 23731 | *TMEM245* | 0.9113 |
|  | ENSG00000104447 | 7227 | *TRPS1* | 0.9113 |
|  | ENSG00000128000 | 163131 | *ZNF780B* | 0.9113 |
|  | ENSG00000161533 | 51 | *ACOX1* | 0.9112 |
|  | ENSG00000018510 | 8540 | *AGPS* | 0.9112 |
|  | ENSG00000149089 | 51074 | *APIP* | 0.9112 |
|  | ENSG00000062725 | 10513 | *APPBP2* | 0.9112 |
|  | ENSG00000108381 | 443 | *ASPA* | 0.9112 |
|  | ENSG00000138696 | 658 | *BMPR1B* | 0.9112 |
|  | ENSG00000187699 | 84281 | *C2orf88* | 0.9112 |
|  | ENSG00000147419 | 55246 | *CCDC25* | 0.9112 |
|  | ENSG00000253276 | 168455 | *CCDC71L* | 0.9112 |
|  | ENSG00000168944 | 153241 | *CEP120* | 0.9112 |
|  | ENSG00000125246 | 171425 | *CLYBL* | 0.9112 |
|  | ENSG00000150672 | 1740 | *DLG2* | 0.9112 |
|  | ENSG00000197959 | 26052 | *DNM3* | 0.9112 |
|  | ENSG00000151151 | 253430 | *IPMK* | 0.9112 |
|  | ENSG00000176049 | 9832 | *JAKMIP2* | 0.9112 |
|  | ENSG00000144320 | 80856 | *KIAA1715* | 0.9112 |
|  | ENSG00000168675 | 753 | *LDLRAD4* | 0.9112 |
|  | ENSG00000107951 | 55149 | *MTPAP* | 0.9112 |
|  | ENSG00000128833 | 55930 | *MYO5C* | 0.9112 |
|  | ENSG00000137819 | 54852 | *PAQR5* | 0.9112 |
|  | ENSG00000151883 | 79668 | *PARP8* | 0.9112 |
|  | ENSG00000137872 | 80031 | *SEMA6D* | 0.9112 |
|  | ENSG00000110013 | 54414 | *SIAE* | 0.9112 |
|  | ENSG00000165970 | 9152 | *SLC6A5* | 0.9112 |
|  | ENSG00000141298 | 85464 | *SSH2* | 0.9112 |
|  | ENSG00000152291 | 10618 | *TGOLN2* | 0.9112 |
|  | ENSG00000109654 | 23321 | *TRIM2* | 0.9112 |
|  | ENSG00000173960 | 165324 | *UBXN2A* | 0.9112 |
|  | ENSG00000196526 | 60312 | *AFAP1* | 0.9111 |
|  | ENSG00000086848 | 79796 | *ALG9* | 0.9111 |
|  | ENSG00000262577 | 79796 | *ALG9* | 0.9111 |
|  | ENSG00000206560 | 23243 | *ANKRD28* | 0.9111 |
|  | ENSG00000213390 | 84986 | *ARHGAP19* | 0.9111 |
|  | ENSG00000165626 | 222389 | *BEND7* | 0.9111 |
|  | ENSG00000163171 | 10602 | *CDC42EP3* | 0.9111 |
|  | ENSG00000111860 | 387119 | *CEP85L* | 0.9111 |
|  | ENSG00000197872 | 81553 | *FAM49A* | 0.9111 |
|  | ENSG00000170802 | 3344 | *FOXN2* | 0.9111 |
|  | ENSG00000119125 | 9615 | *GDA* | 0.9111 |
|  | ENSG00000116983 | 51440 | *HPCAL4* | 0.9111 |
|  | ENSG00000161048 | 222236 | *NAPEPLD* | 0.9111 |
|  | ENSG00000147588 | 5375 | *PMP2* | 0.9111 |
|  | ENSG00000187627 | 400966 | *RGPD1* | 0.9111 |
|  | ENSG00000122545 | 989 | *SEPT7* | 0.9111 |
|  | ENSG00000137776 | 79811 | *SLTM* | 0.9111 |
|  | ENSG00000135913 | 57695 | *USP37* | 0.9111 |
|  | ENSG00000120008 | 55717 | *WDR11* | 0.9111 |
|  | ENSG00000188419 | 1121 | *CHM* | 0.911 |
|  | ENSG00000175595 | 2072 | *ERCC4* | 0.911 |
|  | ENSG00000138675 | 2250 | *FGF5* | 0.911 |
|  | ENSG00000146243 | 134728 | *IRAK1BP1* | 0.911 |
|  | ENSG00000146006 | 26045 | *LRRTM2* | 0.911 |
|  | ENSG00000141027 | 9611 | *NCOR1* | 0.911 |
|  | ENSG00000244462 | 10137 | *RBM12* | 0.911 |
|  | ENSG00000138593 | 9728 | *SECISBP2L* | 0.911 |
|  | ENSG00000101596 | 23347 | *SMCHD1* | 0.911 |
|  | ENSG00000135049 | 23287 | *AGTPBP1* | 0.9109 |
|  | ENSG00000113273 | 411 | *ARSB* | 0.9109 |
|  | ENSG00000112276 | 11149 | *BVES* | 0.9109 |
|  | ENSG00000154642 | 54149 | *C21orf91* | 0.9109 |
|  | ENSG00000074603 | 54878 | *DPP8* | 0.9109 |
|  | ENSG00000217128 | 96459 | *FNIP1* | 0.9109 |
|  | ENSG00000136542 | 11227 | *GALNT5* | 0.9109 |
|  | ENSG00000047932 | 57120 | *GOPC* | 0.9109 |
|  | ENSG00000082701 | 2932 | *GSK3B* | 0.9109 |
|  | ENSG00000152402 | 2977 | *GUCY1A2* | 0.9109 |
|  | ENSG00000233822 | 8341 | *HIST1H2BN* | 0.9109 |
|  | ENSG00000100281 | 10042 | *HMGXB4* | 0.9109 |
|  | ENSG00000215271 | 57594 | *HOMEZ* | 0.9109 |
|  | ENSG00000087253 | 54947 | *LPCAT2* | 0.9109 |
|  | ENSG00000001461 | 57185 | *NIPAL3* | 0.9109 |
|  | ENSG00000127329 | 5787 | *PTPRB* | 0.9109 |
|  | ENSG00000108443 | 6198 | *RPS6KB1* | 0.9109 |
|  | ENSG00000021574 | 6683 | *SPAST* | 0.9109 |
|  | ENSG00000179152 | 285343 | *TCAIM* | 0.9109 |
|  | ENSG00000100234 | 7078 | *TIMP3* | 0.9109 |
|  | ENSG00000214128 | 155006 | *TMEM213* | 0.9109 |
|  | ENSG00000085449 | 57590 | *WDFY1* | 0.9109 |
|  | ENSG00000183579 | 84133 | *ZNRF3* | 0.9109 |
|  | ENSG00000174600 | 1240 | *CMKLR1* | 0.9108 |
|  | ENSG00000268765 | 1536 | *CYBB* | 0.9108 |
|  | ENSG00000165168 | 1536 | *CYBB* | 0.9108 |
|  | ENSG00000115137 | 51277 | *DNAJC27* | 0.9108 |
|  | ENSG00000077458 | 143684 | *FAM76B* | 0.9108 |
|  | ENSG00000132849 | 10207 | *INADL* | 0.9108 |
|  | ENSG00000166006 | 3747 | *KCNC2* | 0.9108 |
|  | ENSG00000145794 | 84466 | *MEGF10* | 0.9108 |
|  | ENSG00000166928 | 84689 | *MS4A14* | 0.9108 |
|  | ENSG00000269514 | NA | *NA* | 0.9108 |
|  | ENSG00000153989 | 116150 | *NUS1* | 0.9108 |
|  | ENSG00000091039 | 114882 | *OSBPL8* | 0.9108 |
|  | ENSG00000180914 | 5021 | *OXTR* | 0.9108 |
|  | ENSG00000040199 | 23035 | *PHLPP2* | 0.9108 |
|  | ENSG00000145675 | 5295 | *PIK3R1* | 0.9108 |
|  | ENSG00000123739 | 81579 | *PLA2G12A* | 0.9108 |
|  | ENSG00000064933 | 5378 | *PMS1* | 0.9108 |
|  | ENSG00000255529 | 81488 | *POLR2M* | 0.9108 |
|  | ENSG00000235194 | 90673 | *PPP1R3E* | 0.9108 |
|  | ENSG00000132356 | 5562 | *PRKAA1* | 0.9108 |
|  | ENSG00000116132 | 5396 | *PRRX1* | 0.9108 |
|  | ENSG00000119042 | 23314 | *SATB2* | 0.9108 |
|  | ENSG00000119760 | 9913 | *SUPT7L* | 0.9108 |
|  | ENSG00000163762 | 116441 | *TM4SF18* | 0.9108 |
|  | ENSG00000189180 | 7581 | *ZNF33A* | 0.9108 |
|  | ENSG00000182983 | 389114 | *ZNF662* | 0.9108 |
|  | ENSG00000166734 | 113201 | *CASC4* | 0.9107 |
|  | ENSG00000143776 | 8476 | *CDC42BPA* | 0.9107 |
|  | ENSG00000104218 | 79848 | *CSPP1* | 0.9107 |
|  | ENSG00000213047 | 163486 | *DENND1B* | 0.9107 |
|  | ENSG00000253626 | 143244 | *EIF5AL1* | 0.9107 |
|  | ENSG00000052795 | 57600 | *FNIP2* | 0.9107 |
|  | ENSG00000163820 | 79443 | *FYCO1* | 0.9107 |
|  | ENSG00000169224 | 148823 | *GCSAML* | 0.9107 |
|  | ENSG00000163349 | 204851 | *HIPK1* | 0.9107 |
|  | ENSG00000110422 | 10114 | *HIPK3* | 0.9107 |
|  | ENSG00000185811 | 10320 | *IKZF1* | 0.9107 |
|  | ENSG00000115221 | 3694 100505984 | *ITGB6* | 0.9107 |
|  | ENSG00000263138 | 84465 | *MEGF11* | 0.9107 |
|  | ENSG00000197498 | 84154 | *RPF2* | 0.9107 |
|  | ENSG00000172164 | 6641 | *SNTB1* | 0.9107 |
|  | ENSG00000078269 | 8871 | *SYNJ2* | 0.9107 |
|  | ENSG00000170703 | 284076 | *TTLL6* | 0.9107 |
|  | ENSG00000182287 | 8905 | *AP1S2* | 0.9106 |
|  | ENSG00000107669 | 11101 | *ATE1* | 0.9106 |
|  | ENSG00000185344 | 23545 | *ATP6V0A2* | 0.9106 |
|  | ENSG00000185515 | 79184 | *BRCC3* | 0.9106 |
|  | ENSG00000269884 | 79184 | *BRCC3* | 0.9106 |
|  | ENSG00000185716 | 730094 101060634 | *C16orf52* | 0.9106 |
|  | ENSG00000157181 | 54953 | *C1orf27* | 0.9106 |
|  | ENSG00000175455 | 64770 | *CCDC14* | 0.9106 |
|  | ENSG00000121005 | 83690 | *CRISPLD1* | 0.9106 |
|  | ENSG00000024526 | 55635 | *DEPDC1* | 0.9106 |
|  | ENSG00000136160 | 1910 | *EDNRB* | 0.9106 |
|  | ENSG00000164307 | 51752 | *ERAP1* | 0.9106 |
|  | ENSG00000158161 | 2140 | *EYA3* | 0.9106 |
|  | ENSG00000206053 | 90861 | *HN1L* | 0.9106 |
|  | ENSG00000078747 | 83737 | *ITCH* | 0.9106 |
|  | ENSG00000138448 | 3685 | *ITGAV* | 0.9106 |
|  | ENSG00000084093 | 5978 | *REST* | 0.9106 |
|  | ENSG00000196935 | 57522 | *SRGAP1* | 0.9106 |
|  | ENSG00000116205 | 127428 | *TCEANC2* | 0.9106 |
|  | ENSG00000188227 | 390927 | *ZNF793* | 0.9106 |
|  | ENSG00000127152 | 64919 | *BCL11B* | 0.9105 |
|  | ENSG00000204446 | 401535 | *C9orf170* | 0.9105 |
|  | ENSG00000151882 | 56477 | *CCL28* | 0.9105 |
|  | ENSG00000175497 | 57628 | *DPP10* | 0.9105 |
|  | ENSG00000120533 | 56943 | *ENY2* | 0.9105 |
|  | ENSG00000086619 | 56605 | *ERO1LB* | 0.9105 |
|  | ENSG00000171262 | 283742 | *FAM98B* | 0.9105 |
|  | ENSG00000180998 | 283554 | *GPR137C* | 0.9105 |
|  | ENSG00000162929 | 84542 | *KIAA1841* | 0.9105 |
|  | ENSG00000179630 | 144811 | *LACC1* | 0.9105 |
|  | ENSG00000117598 | 163404 | *NA* | 0.9105 |
|  | ENSG00000213281 | 4893 | *NRAS* | 0.9105 |
|  | ENSG00000122008 | 51426 | *POLK* | 0.9105 |
|  | ENSG00000152192 | 5457 | *POU4F1* | 0.9105 |
|  | ENSG00000147606 | 115111 | *SLC26A7* | 0.9105 |
|  | ENSG00000143469 | 255928 | *SYT14* | 0.9105 |
|  | ENSG00000180694 | 169200 | *TMEM64* | 0.9105 |
|  | ENSG00000100815 | 9321 | *TRIP11* | 0.9105 |
|  | ENSG00000182986 | 162967 | *ZNF320* | 0.9105 |
|  | ENSG00000180376 | 285331 | *CCDC66* | 0.9104 |
|  | ENSG00000081377 | 8555 | *CDC14B* | 0.9104 |
|  | ENSG00000167258 | 51755 | *CDK12* | 0.9104 |
|  | ENSG00000164330 | 1879 | *EBF1* | 0.9104 |
|  | ENSG00000155511 | 2890 | *GRIA1* | 0.9104 |
|  | ENSG00000164116 | 2982 | *GUCY1A3* | 0.9104 |
|  | ENSG00000010704 | 3077 | *HFE* | 0.9104 |
|  | ENSG00000043462 | 3937 | *LCP2* | 0.9104 |
|  | ENSG00000072736 | 4775 | *NFATC3* | 0.9104 |
|  | ENSG00000130669 | 10298 | *PAK4* | 0.9104 |
|  | ENSG00000112701 | 26054 | *SENP6* | 0.9104 |
|  | ENSG00000147010 | 30011 | *SH3KBP1* | 0.9104 |
|  | ENSG00000091656 | 79776 | *ZFHX4* | 0.9104 |
|  | ENSG00000146463 | 9202 | *ZMYM4* | 0.9104 |
|  | ENSG00000120868 | 317 | *APAF1* | 0.9103 |
|  | ENSG00000164603 | 154743 | *C7orf60* | 0.9103 |
|  | ENSG00000185015 | 377677 100507258 | *CA13* | 0.9103 |
|  | ENSG00000163006 | 165055 | *CCDC138* | 0.9103 |
|  | ENSG00000011465 | 1634 | *DCN* | 0.9103 |
|  | ENSG00000124882 | 2069 | *EREG* | 0.9103 |
|  | ENSG00000135842 | 116496 | *FAM129A* | 0.9103 |
|  | ENSG00000198060 | 54708 | *MARCH5* | 0.9103 |
|  | ENSG00000151322 | 64067 | *NPAS3* | 0.9103 |
|  | ENSG00000139946 | 57161 | *PELI2* | 0.9103 |
|  | ENSG00000212993 | 5462 | *POU5F1B* | 0.9103 |
|  | ENSG00000121486 | 81627 | *TRMT1L* | 0.9103 |
|  | ENSG00000042781 | 7399 | *USH2A* | 0.9103 |
|  | ENSG00000127831 | 7429 | *VIL1* | 0.9103 |
|  | ENSG00000163512 | 64343 | *AZI2* | 0.9102 |
|  | ENSG00000141076 | 84916 | *CIRH1A* | 0.9102 |
|  | ENSG00000221818 | 64641 | *EBF2* | 0.9102 |
|  | ENSG00000113578 | 2246 | *FGF1* | 0.9102 |
|  | ENSG00000122068 | 84248 | *FYTTD1* | 0.9102 |
|  | ENSG00000121743 | 2700 | *GJA3* | 0.9102 |
|  | ENSG00000137807 | 9493 | *KIF23* | 0.9102 |
|  | ENSG00000156928 | 115416 | *MALSU1* | 0.9102 |
|  | ENSG00000188729 | 344901 | *OSTN* | 0.9102 |
|  | ENSG00000124171 | 84612 | *PARD6B* | 0.9102 |
|  | ENSG00000168300 | 115294 | *PCMTD1* | 0.9102 |
|  | ENSG00000107290 | 23064 | *SETX* | 0.9102 |
|  | ENSG00000125255 | 6555 | *SLC10A2* | 0.9102 |
|  | ENSG00000169762 | 202018 | *TAPT1* | 0.9102 |
|  | ENSG00000187824 | 388335 | *TMEM220* | 0.9102 |
|  | ENSG00000213186 | 286827 | *TRIM59* | 0.9102 |
|  | ENSG00000143494 | 79805 | *VASH2* | 0.9102 |
|  | ENSG00000151388 | 81792 | *ADAMTS12* | 0.9101 |
|  | ENSG00000136044 | 55198 | *APPL2* | 0.9101 |
|  | ENSG00000127249 | 84239 | *ATP13A4* | 0.9101 |
|  | ENSG00000133641 | 91298 | *C12orf29* | 0.9101 |
|  | ENSG00000188916 | 642938 | *FAM196A* | 0.9101 |
|  | ENSG00000169752 | 145957 | *NRG4* | 0.9101 |
|  | ENSG00000170381 | 9723 | *SEMA3E* | 0.9101 |
|  | ENSG00000172315 | 112858 | *TP53RK* | 0.9101 |
|  | ENSG00000211689 | 445347 | *TRGC1* | 0.9101 |
|  | ENSG00000164253 | 55255 | *WDR41* | 0.9101 |
|  | ENSG00000089234 | 8315 | *BRAP* | 0.91 |
|  | ENSG00000156535 | 135228 | *CD109* | 0.91 |
|  | ENSG00000150768 | 1737 | *DLAT* | 0.91 |
|  | ENSG00000171931 | 10517 100996906 | *FBXW10* | 0.91 |
|  | ENSG00000152580 | 285313 | *IGSF10* | 0.91 |
|  | ENSG00000177694 | 254827 | *NAALADL2* | 0.91 |
|  | ENSG00000105835 | 10135 | *NAMPT* | 0.91 |
|  | ENSG00000166405 | 79608 | *RIC3* | 0.91 |
|  | ENSG00000029363 | 9774 | *BCLAF1* | 0.9099 |
|  | ENSG00000178562 | 940 | *CD28* | 0.9099 |
|  | ENSG00000174428 | 389524 | *GTF2IRD2B* | 0.9099 |
|  | ENSG00000198121 | 1902 | *LPAR1* | 0.9099 |
|  | ENSG00000105976 | 4233 | *MET* | 0.9099 |
|  | ENSG00000145388 | 57721 | *METTL14* | 0.9099 |
|  | ENSG00000065243 | 5586 | *PKN2* | 0.9099 |
|  | ENSG00000144057 | 84620 | *ST6GAL2* | 0.9099 |
|  | ENSG00000111911 | 135114 | *HINT3* | 0.9098 |
|  | ENSG00000150687 | 11098 | *PRSS23* | 0.9098 |
|  | ENSG00000073756 | 5743 | *PTGS2* | 0.9098 |
|  | ENSG00000055917 | 23369 | *PUM2* | 0.9098 |
|  | ENSG00000102218 | 6102 | *RP2* | 0.9098 |
|  | ENSG00000271091 | 6102 | *RP2* | 0.9098 |
|  | ENSG00000013392 | 112611 | *RWDD2A* | 0.9098 |
|  | ENSG00000091137 | 5172 | *SLC26A4* | 0.9098 |
|  | ENSG00000147853 | 50808 | *AK3* | 0.9097 |
|  | ENSG00000198535 | 145741 | *C2CD4A* | 0.9097 |
|  | ENSG00000076716 | 2239 | *GPC4* | 0.9097 |
|  | ENSG00000107937 | 23560 | *GTPBP4* | 0.9097 |
|  | ENSG00000111912 | 135112 | *NCOA7* | 0.9097 |
|  | ENSG00000188219 | 445582 | *POTEE* | 0.9097 |
|  | ENSG00000069974 | 5873 | *RAB27A* | 0.9097 |
|  | ENSG00000171509 | 59350 | *RXFP1* | 0.9097 |
|  | ENSG00000157800 | 84255 | *SLC37A3* | 0.9097 |
|  | ENSG00000115616 | 6549 | *SLC9A2* | 0.9097 |
|  | ENSG00000137501 | 54843 | *SYTL2* | 0.9097 |
|  | ENSG00000188133 | 401498 | *TMEM215* | 0.9097 |
|  | ENSG00000166479 | 54495 | *TMX3* | 0.9097 |
|  | ENSG00000077097 | 7155 | *TOP2B* | 0.9097 |
|  | ENSG00000204104 | 26146 | *TRAF3IP1* | 0.9097 |
|  | ENSG00000077522 | 88 | *ACTN2* | 0.9096 |
|  | ENSG00000107518 | 26033 | *ATRNL1* | 0.9096 |
|  | ENSG00000166266 | 8065 | *CUL5* | 0.9096 |
|  | ENSG00000178700 | 200895 | *DHFRL1* | 0.9096 |
|  | ENSG00000139684 | 2098 | *ESD* | 0.9096 |
|  | ENSG00000119912 | 3416 | *IDE* | 0.9096 |
|  | ENSG00000102554 | 688 | *KLF5* | 0.9096 |
|  | ENSG00000198961 | 9867 | *PJA2* | 0.9096 |
|  | ENSG00000123091 | 26994 | *RNF11* | 0.9096 |
|  | ENSG00000262275 | 9120 | *SLC16A6* | 0.9096 |
|  | ENSG00000108932 | 9120 | *SLC16A6* | 0.9096 |
|  | ENSG00000064225 | 10402 | *ST3GAL6* | 0.9096 |
|  | ENSG00000174695 | 153339 | *TMEM167A* | 0.9096 |
|  | ENSG00000204186 | 57683 | *ZDBF2* | 0.9096 |
|  | ENSG00000154188 | 284 | *ANGPT1* | 0.9095 |
|  | ENSG00000176597 | 84002 | *B3GNT5* | 0.9095 |
|  | ENSG00000135535 | 8763 | *CD164* | 0.9095 |
|  | ENSG00000117151 | 1486 | *CTBS* | 0.9095 |
|  | ENSG00000163029 | 79677 | *SMC6* | 0.9095 |
|  | ENSG00000153914 | 140890 | *SREK1* | 0.9095 |
|  | ENSG00000215717 | 56900 | *TMEM167B* | 0.9095 |
|  | ENSG00000084652 | 200081 | *TXLNA* | 0.9095 |
|  | ENSG00000088035 | 29929 | *ALG6* | 0.9094 |
|  | ENSG00000001629 | 54467 | *ANKIB1* | 0.9094 |
|  | ENSG00000088854 | 25943 | *C20orf194* | 0.9094 |
|  | ENSG00000163539 | 23122 | *CLASP2* | 0.9094 |
|  | ENSG00000175874 | 200407 | *CREG2* | 0.9094 |
|  | ENSG00000001561 | 22875 | *ENPP4* | 0.9094 |
|  | ENSG00000166262 | 196951 | *FAM227B* | 0.9094 |
|  | ENSG00000143520 | 388698 | *FLG2* | 0.9094 |
|  | ENSG00000171596 | 10316 | *NMUR1* | 0.9094 |
|  | ENSG00000010319 | 56920 | *SEMA3G* | 0.9094 |
|  | ENSG00000163069 | 6443 | *SGCB* | 0.9094 |
|  | ENSG00000172296 | 55304 | *SPTLC3* | 0.9094 |
|  | ENSG00000141431 | 80816 | *ASXL3* | 0.9093 |
|  | ENSG00000196166 | 389649 | *C8orf86* | 0.9093 |
|  | ENSG00000162998 | 2487 | *FRZB* | 0.9093 |
|  | ENSG00000130164 | 3949 | *LDLR* | 0.9093 |
|  | ENSG00000163818 | 54585 | *LZTFL1* | 0.9093 |
|  | ENSG00000189184 | 54510 | *PCDH18* | 0.9093 |
|  | ENSG00000166128 | 51762 | *RAB8B* | 0.9093 |
|  | ENSG00000101166 | 51012 | *SLMO2* | 0.9093 |
|  | ENSG00000014123 | 23376 | *UFL1* | 0.9093 |
|  | ENSG00000204420 | 80739 | *C6orf25* | 0.9092 |
|  | ENSG00000145833 | 9879 | *DDX46* | 0.9092 |
|  | ENSG00000150051 | 283078 | *MKX* | 0.9092 |
|  | ENSG00000234631 | NA | *NA* | 0.9092 |
|  | ENSG00000106443 | 9678 | *PHF14* | 0.9092 |
|  | ENSG00000115963 | 390 | *RND3* | 0.9092 |
|  | ENSG00000165209 | 55342 | *STRBP* | 0.9092 |
|  | ENSG00000184012 | 7113 | *TMPRSS2* | 0.9092 |
|  | ENSG00000198554 | 11169 | *WDHD1* | 0.9092 |
|  | ENSG00000147576 | 137872 | *ADHFE1* | 0.9091 |
|  | ENSG00000165923 | 79841 | *AGBL2* | 0.9091 |
|  | ENSG00000145349 | 817 | *CAMK2D* | 0.9091 |
|  | ENSG00000132164 | 6538 | *SLC6A11* | 0.9091 |
|  | ENSG00000139668 | 115825 | *WDFY2* | 0.9091 |
|  | ENSG00000185480 | 55010 | *PARPBP* | 0.909 |
|  | ENSG00000143882 | 245973 | *ATP6V1C2* | 0.9089 |
|  | ENSG00000118276 | 9331 | *B4GALT6* | 0.9089 |
|  | ENSG00000180354 | 222166 | *C7orf41* | 0.9089 |
|  | ENSG00000150048 | 51267 | *CLEC1A* | 0.9089 |
|  | ENSG00000070190 | 27071 | *DAPP1* | 0.9089 |
|  | ENSG00000154359 | 91694 | *LONRF1* | 0.9089 |
|  | ENSG00000066382 | 744 | *MPPED2* | 0.9089 |
|  | ENSG00000127989 | 7978 | *MTERF* | 0.9089 |
|  | ENSG00000111897 | 57515 | *SERINC1* | 0.9089 |
|  | ENSG00000101463 | 79953 | *SYNDIG1* | 0.9089 |
|  | ENSG00000107897 | 91452 | *ACBD5* | 0.9088 |
|  | ENSG00000006831 | 79602 | *ADIPOR2* | 0.9088 |
|  | ENSG00000162688 | 178 | *AGL* | 0.9088 |
|  | ENSG00000108296 | 54883 | *CWC25* | 0.9088 |
|  | ENSG00000164506 | 134957 | *STXBP5* | 0.9088 |
|  | ENSG00000111731 | 9847 | *C2CD5* | 0.9087 |
|  | ENSG00000135045 | 55071 | *C9orf40* | 0.9087 |
|  | ENSG00000119471 | 84263 | *HSDL2* | 0.9087 |
|  | ENSG00000148057 | 414328 | *IDNK* | 0.9087 |
|  | ENSG00000175329 | 91464 | *ISX* | 0.9087 |
|  | ENSG00000132680 | 22889 | *KIAA0907* | 0.9087 |
|  | ENSG00000096717 | 23411 | *SIRT1* | 0.9087 |
|  | ENSG00000149313 | 60496 | *AASDHPPT* | 0.9086 |
|  | ENSG00000146410 | 113115 | *MTFR2* | 0.9086 |
|  | ENSG00000170448 | 152518 | *NFXL1* | 0.9086 |
|  | ENSG00000102893 | 5257 | *PHKB* | 0.9086 |
|  | ENSG00000187010 | 6007 | *RHD* | 0.9086 |
|  | ENSG00000150540 | 3176 | *HNMT* | 0.9085 |
|  | ENSG00000155970 | 286097 | *MICU3* | 0.9085 |
|  | ENSG00000158604 | 222068 | *TMED4* | 0.9085 |
|  | ENSG00000149260 | 726 | *CAPN5* | 0.9084 |
|  | ENSG00000119285 | 55127 | *HEATR1* | 0.9084 |
|  | ENSG00000165525 | 9147 | *NEMF* | 0.9084 |
|  | ENSG00000109118 | 57649 | *PHF12* | 0.9084 |
|  | ENSG00000138069 | 5861 | *RAB1A* | 0.9084 |
|  | ENSG00000100372 | 10478 | *SLC25A17* | 0.9084 |
|  | ENSG00000145757 | 83890 | *SPATA9* | 0.9084 |
|  | ENSG00000114541 | 23150 | *FRMD4B* | 0.9083 |
|  | ENSG00000127311 | 92797 | *HELB* | 0.9083 |
|  | ENSG00000258881 | NA | *NA* | 0.9083 |
|  | ENSG00000271961 | NA | *NA* | 0.9083 |
|  | ENSG00000004799 | 5166 | *PDK4* | 0.9083 |
|  | ENSG00000170325 | 56980 | *PRDM10* | 0.9083 |
|  | ENSG00000165512 | 7570 | *ZNF22* | 0.9083 |
|  | ENSG00000155011 | 27123 | *DKK2* | 0.9082 |
|  | ENSG00000147162 | 8473 | *OGT* | 0.9082 |
|  | ENSG00000112308 | 81688 | *C6orf62* | 0.9081 |
|  | ENSG00000006074 | 6362 101060271 | *CCL18* | 0.9081 |
|  | ENSG00000006837 | 51265 | *CDKL3* | 0.9081 |
|  | ENSG00000035141 | 84908 | *FAM136A* | 0.9081 |
|  | ENSG00000072042 | 51109 | *RDH11* | 0.9081 |
|  | ENSG00000101413 | 58490 | *RPRD1B* | 0.9081 |
|  | ENSG00000109436 | 23158 | *TBC1D9* | 0.9081 |
|  | ENSG00000047188 | 64848 | *YTHDC2* | 0.9081 |
|  | ENSG00000183643 | 145858 | *C15orf32* | 0.908 |
|  | ENSG00000163946 | 23272 | *FAM208A* | 0.908 |
|  | ENSG00000176018 | 116068 | *LYSMD3* | 0.908 |
|  | ENSG00000174891 | 51319 | *RSRC1* | 0.908 |
|  | ENSG00000102445 | 80183 | *KIAA0226L* | 0.9079 |
|  | ENSG00000267059 | 10975 | *NA* | 0.9079 |
|  | ENSG00000167332 | 81285 | *OR51E2* | 0.9079 |
|  | ENSG00000101901 | 79868 | *ALG13* | 0.9078 |
|  | ENSG00000127540 | 10975 | *UQCR11* | 0.9078 |
|  | ENSG00000050165 | 27122 | *DKK3* | 0.9077 |
|  | ENSG00000185610 | 440097 | *DBX2* | 0.9076 |
|  | ENSG00000164484 | 114801 | *TMEM200A* | 0.9076 |
|  | ENSG00000224982 | 387890 | *TMEM233* | 0.9076 |
|  | ENSG00000065883 | 8621 | *CDK13* | 0.9075 |
|  | ENSG00000183340 | 8690 | *JRKL* | 0.9075 |
|  | ENSG00000184434 | 64922 | *LRRC19* | 0.9074 |
|  | ENSG00000123505 | 262 | *AMD1* | 0.9073 |
|  | ENSG00000009307 | 7812 | *CSDE1* | 0.9073 |
|  | ENSG00000196604 | 728378 | *POTEF* | 0.9073 |
|  | ENSG00000131042 | 10288 | *LILRB2* | 0.9072 |
|  | ENSG00000271481 | 10288 | *LILRB2* | 0.9072 |
|  | ENSG00000118600 | 10329 | *TMEM5* | 0.9072 |
|  | ENSG00000075539 | 285527 | *FRYL* | 0.9071 |
|  | ENSG00000157045 | 123803 | *NTAN1* | 0.9071 |
|  | ENSG00000035928 | 5981 | *RFC1* | 0.907 |
|  | ENSG00000103546 | 6530 | *SLC6A2* | 0.9069 |
|  | ENSG00000054118 | 9967 | *THRAP3* | 0.9069 |
|  | ENSG00000213865 | 56260 | *C8orf44* | 0.9068 |
|  | ENSG00000122145 | 50945 | *TBX22* | 0.9068 |
|  | ENSG00000175311 | 257629 | *ANKS4B* | 0.9067 |
|  | ENSG00000133226 | 10250 | *SRRM1* | 0.9067 |
|  | ENSG00000054796 | 23626 | *SPO11* | 0.9059 |
